# Supplementary material for: Promotive and protective effects of community-related positive childhood experiences on adult health outcomes in the context of adverse childhood experiences: a nationwide cross-sectional survey in Japan
Source: BMJ Open. 2024 Jun 25;14(6):e082134. doi: 10.1136/bmjopen-2023-082134 (PMC11202639; doi:10.1136/bmjopen-2023-082134)
Supplement: Supplementary data [file bmjopen-2023-082134supp001.pdf]

Supplemental Material

Table S1 Prevalence of PCE single items among participants (N=28617)

	N	weighted %
FPCEs		
i) Felt able to talk to their family about feelings	9113	28.6
ii) Felt their family stood by them during difficult times	12738	40.0
iii) Felt safe and protected by an adult in their home	14700	45.8
CPCEs		
iv) Had at least 2 nonparent adults who took genuine interest in them	10732	32.8
v) Felt supported by friends	10563	32.1
vi) Felt a sense of belonging in middle and high school	12334	37.9
vii) Enjoyed participating in community traditions	7080	22.1

FPCEs, family positive childhood experiences; CPCEs, community positive childhood experiences

Table S2 Summary of results of psychometric analyses performed on FPCEs and CPCEs

	Internal Consistency (Cronbach's alpha)	Principle components factor analysis results	Factor loading across items
FPCEs	0.81	A single Eigen value greater than 1.0 (2.19) that explained 72.9% of variance	0.54 ("felt able to talk") to 0.61 ("felt safe and protected")
CPCEs	0.75	A single Eigen value greater than 1.0 (2.28) that explained 56.9% of variance	0.46 ("community traditions") to 0.52 ("felt supported by friends")

FPCEs, family positive childhood experiences; CPCEs, community positive childhood experiences

Table S3 Demographic characteristics and prevalence of health outcomes, PCEs by ACE exposure levels

	Prevalence of ACEs				
	0 ACEs	1 ACE	2–3 ACEs	≥4 ACEs	
	N=17889	N=5547	N=3725	N=1456	
	W %/M	W %/M	W %/M	W %/M	P value*
Health outcomes					
Diabetes	6.9	9.1	7.6	10.5	<0.001
Ischemic heart disease	2.7	2.8	3.0	6.8	<0.001
Stroke	1.2	1.4	2.3	5.6	<0.001
COPD	1.1	0.8	2.2	5.7	<0.001
Cancer	2.0	2.8	3.3	6.7	<0.001
Chronic pain	14.7	20.9	26.7	33.1	<0.001
Depression	2.8	3.9	8.6	17.7	<0.001
Suicidal ideation	10.3	13.3	25.7	35.7	<0.001
Severe psychological distress	7.0	8.9	19.0	30.6	<0.001
FPCEs (0–3)	1.3	1.1	0.7	0.3	<0.001
0 FPCE	39.9	47.7	65.0	80.2	<0.001
1 FPCEs	14.4	13.6	13.9	10.7	0.095
2 FPCEs	17.9	17.1	11.8	5.4	<0.001
3 FPCEs	27.8	21.7	9.4	3.7	<0.001
CPCEs (0–4)	1.4	1.2	0.9	0.7	<0.001
0 CPCE	40.4	43.9	52.8	61.8	<0.001
1 CPCEs	18.3	21.1	21.8	18.7	<0.001
2 CPCEs	14.9	15.7	13.0	12.6	0.002
3 CPCEs	13.3	10.4	8.0	4.4	<0.001
4 CPCEs	13.1	8.9	4.5	2.6	<0.001
Age (19–79)	47.4	51.3	47.2	45.2	<0.001
Sex					
Male	52.8	46.2	39.8	38.5	<0.001
Female	47.2	53.8	60.2	61.5	<0.001
Educational attainment					
Less than HS	2.9	7.5	5.0	9.0	<0.001
HS graduate	57.8	62.9	64.8	66.8	<0.001
Post-secondary and graduate	37.8	28.6	28.7	23.1	<0.001

*P value for significant differences by Pearson's χ^2 test or ANOVA.

W, weighted; ACEs, adverse childhood experiences; FPCEs, family positive childhood experiences; CPCEs, community positive childhood experiences; HS, high school

Table S4 Adjusted odds ratios of health outcomes by ACE exposure levels

	**Diabetes**		**Ischemic heart disease**		**Stroke**		**COPD**		**Cancer**		**Chronic pain**		**Depression**		**Suicidal ideation**		**Severe psychological distress**	
	**AOR**	**(95% CI)**	**AOR**	**(95% CI)**	**AOR**	**(95% CI)**	**AOR**	**(95% CI)**	**AOR**	**(95% CI)**	**AOR**	**(95% CI)**	**AOR**	**(95% CI)**	**AOR**	**(95% CI)**	**AOR**	**(95% CI)**
ACEs																		
0 ACEs [reference]	1.00		1.00		1.00		1.00		1.00		1.00		1.00		1.00		1.00	
1 ACE	1.16	(1.02–1.31)	1.08	(0.88–1.33)	1.24	(0.95–1.63)	0.98	(0.71–1.35)	1.06	(0.86–1.30)	1.41	(1.30–1.53)	1.75	(1.49–2.04)	1.65	(1.51–1.82)	1.65	(1.48–1.84)
2–3 ACEs	1.43	(1.24–1.65)	1.78	(1.44–2.21)	2.28	(1.76–2.95)	2.62	(2.01–3.41)	1.90	(1.55–2.34)	1.94	(1.78–2.11)	3.81	(3.30–4.40)	3.16	(2.88–3.47)	3.25	(2.93–3.61)
4 ACEs	1.98	(1.62–2.42)	3.52	(2.73–4.54)	5.21	(3.94–6.89)	5.66	(4.24–7.56)	3.71	(2.90–4.73)	2.93	(2.60–3.30)	8.12	(6.90–9.56)	4.29	(3.78–4.86)	5.94	(5.20–6.78)
Age	1.04	(1.04–1.05)	1.02	(1.01–1.02)	0.99	(0.99–1.00)	0.98	(0.97–0.99)	1.02	(1.02–1.03)	1.01	(1.01–1.01)	0.98	(0.98–0.98)	0.96	(0.96–0.97)	0.96	(0.96–0.96)
Sex																		
Male [reference]	1.00		1.00		1.00		1.00		1.00		1.00		1.00		1.00		1.00	
Female	0.31	(0.28–0.35)	0.34	(0.29–0.40)	0.38	(0.31–0.47)	0.32	(0.26–0.40)	0.73	(0.63–0.85)	1.13	(1.06–1.20)	0.62	(0.55–0.69)	1.02	(0.95–1.09)	0.88	(0.81–0.95)
Educational attainment																		
Less than HS	1.64	(1.22–2.20)	1.29	(0.80–2.07)	0.99	(0.52–1.90)	1.24	(0.74–2.45)	0.75	(0.43–1.32)	1.22	(0.98–1.51)	1.60	(1.17–2.19)	1.66	(1.32–2.09)	1.55	(1.20–1.99)
HS graduate	1.13	(1.02–1.25)	1.05	(0.90–1.24)	1.04	(0.85–1.28)	0.99	(0.79–1.23)	0.82	(0.70–0.97)	1.11	(1.04–1.18)	1.11	(0.99–1.25)	1.08	(1.00–1.16)	1.02	(0.93–1.11)
Post-secondary and graduate [reference]	1.00		1.00		1.00		1.00		1.00		1.00		1.00		1.00		1.00	

AOR, adjusted odds ratio; ACEs, adverse childhood experiences; HS, high school

Table S5 Adjusted odds ratios of health outcomes by ACEs, FPCEs, and CPCEs

	Diabetes		Ischemic heart disease		Stroke		COPD		Cancer		Chronic pain		Depression		Suicidal ideation		Severe psychological distress	
	**AOR**	**(95% CI)**	**AOR**	**(95% CI)**	**AOR**	**(95% CI)**	**AOR**	**(95% CI)**	**AOR**	**(95% CI)**	**AOR**	**(95% CI)**	**AOR**	**(95% CI)**	**AOR**	**(95% CI)**	**AOR**	**(95% CI)**
ACEs																		
0 ACEs [reference]	1.00		1.00		1.00		1.00		1.00		1.00		1.00		1.00		1.00	
≥1 ACEs	1.30	(1.18–1.44)	1.52	(1.30–1.78)	1.92	(1.58–2.33)	1.95	(1.58–2.41)	1.60	(1.37–1.87)	1.70	(1.60–1.82)	2.85	(2.54–3.20)	2.12	(1.97–2.28)	2.28	(2.10–2.48)
FPCEs																		
0–1 FPCEs [reference]	1.00		1.00		1.00		1.00		1.00		1.00		1.00		1.00		1.00	
2 FPCEs	0.99	(0.86–1.15)	0.99	(0.80–1.24)	0.77	(0.59–1.01)	0.77	(0.58–1.02)	0.86	(0.69–1.06)	1.00	(0.91–1.10)	0.88	(0.74–1.06)	0.83	(0.74–0.93)	0.81	(0.71–0.93)
3 FPCEs	0.81	(0.73–0.98)	0.70	(0.55–0.90)	0.50	(0.37–0.70)	0.40	(0.28–0.58)	0.68	(0.54–0.85)	0.95	(0.87–1.05)	0.69	(0.56–0.84)	0.62	(0.55–0.70)	0.64	(0.56–0.74)
CPCEs																		
0–2 CPCEs [reference]	1.00		1.00		1.00		1.00		1.00		1.00		1.00		1.00		1.00	
3 CPCEs	0.96	(0.83–1.12)	0.80	(0.62–1.03)	0.63	(0.44–0.89)	0.76	(0.54–1.07)	0.82	(0.84–1.05)	0.82	(0.74–0.91)	0.62	(0.50–0.78)	0.56	(0.49–0.64)	0.52	(0.45–0.62)
4 CPCEs	0.79	(0.66–0.95)	0.65	(0.47–0.89)	0.52	(0.34–0.82)	0.38	(0.22–0.66)	0.80	(0.61–1.06)	0.75	(0.67–0.84)	0.55	(0.42–0.72)	0.45	(0.38–0.53)	0.40	(0.32–0.49)
Age	1.04	(1.04–1.04)	1.02	(1.01–1.02)	0.99	(0.98–1.00)	0.98	(0.97–0.98)	1.02	(1.01–1.02)	1.01	(1.01–1.01)	0.98	(0.98–0.98)	0.96	(0.96–0.96)	0.96	(0.95–0.96)
Sex																		
Male [reference]	1.00		1.00		1.00		1.00		1.00		1.00		1.00		1.00		1.00	
Female	0.32	(0.29–0.35)	0.36	(0.30–0.42)	0.41	(0.34–0.51)	0.35	(0.28–0.44)	0.75	(0.64–0.87)	1.17	(1.10–1.24)	0.70	(0.63–0.78)	1.17	(1.09–1.26)	1.02	(0.94–1.11)
Educational attainment																		
Less than HS	1.70	(1.27–2.28)	1.45	(0.90–2.33)	1.17	(0.62–2.23)	1.69	(0.93–3.06)	0.90	(0.51–1.57)	1.27	(1.03–1.58)	1.67	(1.23–2.28)	1.57	(1.25–1.97)	1.49	(1.16–1.91)
HS graduate	1.14	(1.03–1.26)	1.08	(0.92–1.27)	1.07	(0.87–1.31)	1.03	(0.82–1.28)	0.85	(0.73–1.00)	1.11	(1.05–1.19)	1.10	(0.98–1.23)	1.03	(0.95–1.11)	0.97	(0.89–1.06)
Post-secondary and graduate [reference]	1.00		1.00		1.00		1.00		1.00		1.00		1.00		1.00		1.00	

AOR, adjusted odds ratio; ACEs, adverse childhood experiences; FPCEs, family positive childhood experiences; CPCEs, community positive childhood; HS, high school

Table S6 Adjusted odds ratios of health outcomes by ACEs, CPCEs, and these interaction terms

	Diabetes		Ischemic heart disease		Stroke		COPD		Cancer		Chronic pain		Depression		Suicidal ideation		Severe psychological distress	
	**AOR**	**(95% CI)**	**AOR**	**(95% CI)**	**AOR**	**(95% CI)**	**AOR**	**(95% CI)**	**AOR**	**(95% CI)**	**AOR**	**(95% CI)**	**AOR**	**(95% CI)**	**AOR**	**(95% CI)**	**AOR**	**(95% CI)**
ACEs																		
0 ACEs [reference]	1.00		1.00		1.00		1.00		1.00		1.00		1.00		1.00		1.00	
≥1 ACEs	1.34	(1.20–1.49)	1.68	(1.42–2.00)	2.02	(1.63–2.49)	2.24	(1.78–2.81)	1.77	(1.49–2.11)	1.78	(1.65–1.91)	2.99	(2.63–3.38)	2.20	(2.03–2.37)	2.35	(2.15–2.56)
CPCEs																		
0–2 CPCEs [reference]	1.00		1.00		1.00		1.00		1.00		1.00		1.00		1.00		1.00	
≥3 CPCEs	0.93	(0.79–1.08)	0.55	(0.70–1.16)	0.68	(0.47–0.98)	0.88	(0.61–1.27)	0.98	(0.77–1.25)	0.85	(0.77–0.94)	0.67	(0.52–0.85)	0.55	(0.47–0.63)	0.48	(0.41–0.57)
Interaction																		
≥1 ACEs × ≥3 CPCEs	0.89	(0.70–1.13)	0.50	(0.36–0.85)	0.70	(0.39–1.23)	0.37	(0.19–0.74)	0.61	(0.41–0.90)	0.82	(0.70–0.96)	0.71	(0.51–1.00)	0.79	(0.64–0.97)	0.80	(0.62–1.04)
Age	1.04	(1.04–1.05)	1.02	(1.01–1.02)	0.99	(0.98–1.00)	0.98	(0.97–0.99)	1.02	(1.01–1.02)	1.01	(1.01–1.01)	0.98	(0.98–0.98)	0.96	(0.96–0.96)	0.96	(0.95–0.96)
Sex																		
Male [reference]	1.00		1.00		1.00		1.00		1.00		1.00		1.00		1.00		1.00	
Female	0.32	(0.29–0.35)	0.35	(0.30–0.42)	0.41	(0.33–0.51)	0.35	(0.27–0.43)	0.74	(0.64–0.87)	1.17	(1.09–1.24)	0.69	(0.62–0.77)	1.15	(1.07–1.24)	1.01	(0.93–1.09)
Educational attainment																		
Less than HS	1.70	(1.27–2.27)	1.43	(0.89–2.30)	1.16	(0.61–2.22)	1.66	(0.91–3.01)	0.88	(0.50–1.55)	1.27	(1.02–1.57)	1.71	(1.26–2.33)	1.60	(1.28–2.01)	1.54	(1.20–1.98)
HS graduate	1.14	(1.03–1.26)	1.08	(0.92–1.27)	1.07	(0.87–1.31)	1.02	(0.82–1.27)	0.86	(0.73–1.00)	1.12	(1.05–1.19)	1.11	(0.99–1.24)	1.03	(0.96–1.12)	0.98	(0.90–1.07)
Post-secondary and graduate [reference]	1.00		1.00		1.00		1.00		1.00		1.00		1.00		1.00		1.00	
FPCEs																		
0–1 FPCEs [reference]	1.00		1.00		1.00		1.00		1.00		1.00		1.00		1.00		1.00	
2 FPCEs	0.99	(0.86–1.15)	0.99	(0.80–1.24)	0.77	(0.58–1.01)	0.76	(0.57–1.01)	0.86	(0.69–1.06)	1.00	(0.91–1.10)	0.79	(0.67–0.93)	0.75	(0.67–0.83)	0.68	(0.60–0.77)
3 FPCEs	0.83	(0.72–0.96)	0.68	(0.54–0.87)	0.49	(0.36–0.68)	0.36	(0.25–0.52)	0.67	(0.53–0.83)	0.94	(0.86–1.03)	0.61	(0.50–0.73)	0.55	(0.49–0.61)	0.53	(0.46–0.60)

AOR, adjusted odds ratio ACEs, adverse childhood experiences; CPCEs, community positive childhood; HS, high school; FPCEs, family positive childhood experiences

Table S7 Marginal effects with test of interaction effects

	**Marginal effects (95% CI)**		**Differences of two marginal effects (95% CI)**	P value*
	0–2 CPCEs	≥3 CPCEs		
Diabetes	0.018<br>(0.011 to 0.025)	0.010<br>(−0.003 to 0.022)	0.008<br>(−0.023 to 0.006)	0.248
Ischemic heart disease	0.013<br>(0.009 to 0.018)	−0.001<br>(−0.008 to 0.006)	0.015<br>(0.006 to 0.023)	0.001
Stroke	0.012<br>(0.008 to 0.016)	0.003<br>(−0.002 to 0.009)	0.009<br>(0.006 to 0.023)	0.011
COPD	0.012<br>(0.008 to 0.016)	−0.002<br>(−0.006 to 0.003)	0.014<br>(0.008 to 0.020)	0.000
Cancer	0.015<br>(0.010 to 0.020)	0.001<br>(−0.006 to 0.009)	0.013<br>(0.005 to 0.022)	0.002
Chronic pain	0.088<br>(0.077 to 0.099)	0.049<br>(0.030 to 0.068)	0.039<br>(0.017 to 0.061)	0.000
Depression	0.055<br>(0.049 to 0.062)	0.023<br>(0.012 to 0.033)	0.033<br>(0.020 to 0.046)	0.000
Suicidal ideation	0.098<br>(0.088 to 0.108)	0.041<br>(0.025 to 0.057)	0.057<br>(0.038 to 0.076)	0.000
Severe psychological distress	0.084<br>(0.075 to 0.092)	0.033<br>(0.019 to 0.046)	0.051<br>(0.035 to 0.067)	0.000

*P value for significant differences by test of the equality of marginal effects (Stata: margins, contrast).
