# Supplementary material for: Promotive and protective effects of community-related positive childhood experiences on adult health outcomes in the context of adverse childhood experiences: a nationwide cross-sectional survey in Japan
Source: BMJ Open. 2024 Jun 25;14(6):e082134. doi: 10.1136/bmjopen-2023-082134 (PMC11202639; doi:10.1136/bmjopen-2023-082134)
Supplement: Author's manuscript [file bmjopen-2023-082134.draft_revisions.pdf]

BMJ Open

BMJ Open is committed to open peer review. As part of this commitment we make the peer review history of every article we publish publicly available.

When an article is published we post the peer reviewers' comments and the authors' responses online. We also post the versions of the paper that were used during peer review. These are the versions that the peer review comments apply to.

The versions of the paper that follow are the versions that were submitted during the peer review process. They are not the versions of record or the final published versions. They should not be cited or distributed as the published version of this manuscript.

BMJ Open is an open access journal and the full, final, typeset and author-corrected version of record of the manuscript is available on our site with no access controls, subscription charges or pay-per-view fees (http://bmjopen.bmj.com).

If you have any questions on BMJ Open's open peer review process please email info.bmjopen@bmj.com

Adverse childhood experiences and adult diseases: role of positive childhood experiences in the community as a promotive and protective factor

Journal:	*BMJ Open*
Manuscript ID	bmjopen-2023-082134
Article Type:	Original research
Date Submitted by the Author:	15-Nov-2023

Complete List of Authors:	Mitani, Haruyo; Ryukoku University<br>Kondo, Naoki; Kyoto University, Social epidemiology<br>Amemiya, Airi; Kyoto University, Social epidemiology (former affiliation)<br>Tabuchi, Takahiro; Osaka International Cancer Institute Cancer Control Center

Keywords:	Chronic Disease, EPIDEMIOLOGY, MENTAL HEALTH, Social Interaction, Stress, Psychological

I, the Submitting Author has the right to grant and does grant on behalf of all authors of the Work (as defined in the below author licence), an exclusive licence and/or a non-exclusive licence for contributions from authors who are: i) UK Crown employees; ii) where BMJ has agreed a CC-BY licence shall apply, and/or iii) in accordance with the terms applicable for US Federal Government officers or employees acting as part of their official duties; on a worldwide, perpetual, irrevocable, royalty-free basis to BMJ Publishing Group Ltd ("BMJ") its licensees and where the relevant Journal is co-owned by BMJ to the co-owners of the Journal, to publish the Work in this journal and any other BMJ products and to exploit all rights, as set out in our licence.

The Submitting Author accepts and understands that any supply made under these terms is made by BMJ to the Submitting Author unless you are acting as an employee on behalf of your employer or a postgraduate student of an affiliated institution which is paying any applicable article publishing charge ("APC") for Open Access articles. Where the Submitting Author wishes to make the Work available on an Open Access basis (and intends to pay the relevant APC), the terms of reuse of such Open Access shall be governed by a Creative Commons licence – details of these licences and which Creative Commons licence will apply to this Work are set out in our licence referred to above.

Other than as permitted in any relevant BMJ Author's Self Archiving Policies, I confirm this Work has not been accepted for publication elsewhere, is not being considered for publication elsewhere and does not duplicate material already published. I confirm all authors consent to publication of this Work and authorise the granting of this licence.

For peer review only

Title

Adverse childhood experiences and adult diseases: role of positive childhood experiences in the community as a promotive and protective factor

Authors

Haruyo Mitani[1], Naoki Kondo[2], Airi Amemiya, Takahiro Tabuchi[3]

Author Affiliations

[1]Faculty of Sociology, Ryukoku University, Kyoto, Japan

[2]Department of Social Epidemiology, Graduate School of Medicine, School of Public Health, Kyoto University, Kyoto, Japan

[3]Cancer Control Center, Osaka International Cancer Institute, Osaka, Japan

Correspondence to

Haruyo Mitani, Faculty of Sociology, Ryukoku University, Kyoto, Japan

haruyo.mitani@gmail.com

For peer review only

ABSTRACT

Objective: Although adverse childhood experiences (ACEs) are associated with poor health in adulthood, positive childhood experiences (PCEs) can reduce the risk of negative health outcomes. This study aimed to investigate whether PCEs in the community (CPCEs; i.e., trusted adults other than parents, supportive friends, belongingness to school, or community traditions) reduce the risk of acquiring diseases during adulthood.

Design and setting: A nationwide, cross-sectional Internet survey was conducted in Japan in 2022. This study included 28,254 Japanese participants aged 18–79 years (51.1% female; mean age = 47.6 years). The associations among self-reported ACEs, CPCEs before the age of 18 years, and current chronic diseases (e.g., cancer and depression) were investigated using multivariable logistic regression models. This study examined whether CPCEs would have an independent effect on better health outcomes and moderate the association between ACEs and adult illnesses.

Results: CPCEs were associated with lower odds of adult diseases (such as stroke, chronic obstructive pulmonary disease [COPD], chronic pain, depression, suicidal ideation, and severe psychological distress) after adjusting for ACEs. More CPCEs weakened the association between ACEs and adult diseases. Specifically, among those with ACEs, ≥3 CPCEs (vs. 0–2 CPCEs) reduced the adjusted prevalence by ≥50% for ischemic heart disease (3.4→1.6%), COPD (2.3→0.7%), depression (8.7→4.4%), and severe psychological distress (16.5→7.5%).

Conclusion: CPCEs could reduce ACE-related risk of poor physical and mental health in later life. Early-life interventions that enhance PCEs in schools and/or neighborhoods are recommended.

For peer review only - http://bmjopen.bmj.com/site/about/guidelines.xhtml

Strengths and limitations of this study

- This study used a large and representative sample from a nationwide survey in Japan.
- This study focused on the role of CPCEs for countering the negative lifetime effects of ACEs.
- Multivariable logistic regression models were used to adjust for possible confounders and to evaluate the associations among ACEs, CPCEs, and adult diseases.
- Due to the self-reported and retrospective nature of the survey data, the possibility of recall bias and reverse causation cannot be eliminated.
- The CPCEs measure used in this study did not include all CPCEs.

For peer review only

For peer review only - http://bmjopen.bmj.com/site/about/guidelines.xhtml

INTRODUCTION

Over the last quarter-century, a growing body of research has demonstrated the harmful effects of adverse childhood experiences (ACEs), such as maltreatment and exposure to household dysfunction, on long-term physical and mental health[1–6]. Studies have emphasized a dose-response relationship between an individual's ACE score and their risk of having poor health. Trauma-related extreme stress impairs the stress response and self-regulation capacity, disrupting typical brain development[7,8].

Meanwhile, recent evidence shows that positive childhood experiences (PCEs) can reduce the risk of negative outcomes due to ACEs[9–15]. For example, continual early assistance from a reliable adult[9], an adult who felt safe and protected as a child[12], or PCEs inside and outside the home (cumulative scale consisting of seven items such as "able to talk to family about feelings" and "felt supported by friends")[10], moderates the association between ACEs and negative consequences, represented by poor mental health.

Little is understood about the potential of PCEs in the community (CPCEs) for countering the negative lifetime effects of ACEs. Resilience refers to the latent or apparent capacity to successfully respond to situations that threaten an individual's functioning, survival, or development[16]. Based on the Resilience Theory[16], the socio-ecological system suggests that multiple systems (e.g., individuals, families, and communities) complementarily influence youth development. Moreover, factors promoting resilient outcomes can be divided into individual (e.g., cognition, coping style, and self-esteem), family (e.g., a safe and supportive relationship with a caregiver), and community factors (e.g., positive relationships with friends and neighbors)[16–19]. Notably, the concept of PCEs includes both family and community factors.

The present study focused on the role of CPCEs (PCEs in the community outside the home).

For peer review only - http://bmjopen.bmj.com/site/about/guidelines.xhtml

Children exposed to ACEs often have ACE in their relationship with their caregivers, not PCE. Hence, continuous family intervention by professionals to change how caregivers relate to children is desirable to enrich family PCEs (FPCEs)[20]. However, many countries lack the infrastructure (staff, funds, and legal structure) to make such professional intervention possible, and Japan is no exception[21]. However, community interventions in schools and neighborhoods can easily and inexpensively positively impact the development of children exposed to ACEs. Clarifying the role of CPCEs is essential for understanding the effectiveness of community interventions.

Therefore, this study explored the effect of CPCEs on the negative consequences of ACEs, focusing on four items of the PCEs scale[10], referenced in many studies, that are considered particularly present in the community (i.e., trusted adults other than parents, supportive friends, belongingness to school, and community traditions). Similar to the original ACE study[1], this study used a broad range of physical and mental illnesses as primary outcomes. In Japan, although ACEs affect physical illnesses in old age[22] and mental disorders in adults[23], the interaction between PCEs and ACEs on health has not been examined.

This study aimed to explore the associations among ACEs, CPCEs, and adult diseases. First, the relationship between ACEs and adult diseases was ascertained. Then, the Resilience Theory's two main models (the Compensatory Model and the Protective Factors Model[16,24,25]) were examined. The Compensatory (Promotive Factors) Model assumes that promotive/compensatory factors directly affect developmental outcomes and neutralize the harm of risk factors. The Protective Factors Model assumes that protective factors mitigate the relationship between risk factors and bad outcomes. Thus, this study tested two hypotheses: CPCEs directly affect adult health (H1), and the association between ACEs and adult diseases is weaker among people with more CPCEs (H2).

For peer review only - http://bmjopen.bmj.com/site/about/guidelines.xhtml

METHODS

Sample

The Japan COVID-19 and Society Internet Survey (JACSIS)[26] was conducted in 2022 by a major Internet research agency (Rakuten Insight, Inc.) with around 2.2 million registered qualified panelists. Of the panelists, 224,389 males and females aged 15–79 were invited to participate in the survey based on a random sample stratified by sex, age, and all 47 prefectures. Participants were rewarded with "E-Points" for online shopping. Before the survey, all participants provided informed consent. The survey began on September 12, 2022 and ended on October 19, 2022. The survey was terminated after the target of 32,000 participants was reached.

This study was authorized by the Research Ethics Committee of the Osaka International Cancer Institute (approved on June 14, 2022; approval No. 20084-8) and followed the Helsinki guidelines.

Participants who provided contradictory answers to the screening questions were excluded from the study. These included the following criteria: (i) invalid answers to the screening question (participants were asked to choose the second-last option, but they chose other options), (ii) positive responses to all items on drug use, (iii) positive answers for having all 20 chronic diseases, and (iv) a total household size of over 15 individuals. There were 3,370 responders with inconsistent answers, leaving 28,630 individuals. Those aged <18 or >79 years were also excluded. Finally, 28,254 adults were included.

For peer review only - http://bmjopen.bmj.com/site/about/guidelines.xhtml

Patient and public involvement

Neither patients nor the public were involved in this study.

Measurements

ACEs

Questions on ACEs were derived from the ACEs scale for the Japanese context[27]. Although this study was based on the original ACE study[1], ACE items included parental death, except the incarceration of household members, to fit the Japanese context. Respondents were questioned whether any of the following adversities had occurred in their households by the age of 18 years: (i) death of either parent; (ii) parental divorce or separation; (iii) mental illness of either parent; (iv) substance abuse in the household (e.g., parental addiction to alcohol and gambling); (v) mother treated violently (the father was violent with the mother); (vi) emotional abuse (my parent said hurtful things or insulted me); (vii) physical abuse (my parent beat and hurt me); (viii) sexual abuse (an adult sexually touched me); (ix) emotional neglect (I always felt suffocated because my parent did not respect my opinion); and (x) physical neglect (I did not receive necessary care, such as eating and dressing). The response categories "yes" and "no" were coded as dummy variables, and these 10 items were summed into ACE scores (0–10). This score was classified into two categories (0 vs. ≥1) because of their effectiveness in determining poor health, following those of previous studies[22,28].

PCEs

PCEs were assessed using seven items adapted from the PCEs scale[10]. Participants answered

For peer review only - http://bmjopen.bmj.com/site/about/guidelines.xhtml

whether they had experienced any of the following by the time they were 18 years old: (i) felt able to talk to their family about feelings; (ii) felt their family stood by them during difficult times; (iii) felt safe and protected by an adult in their home; (iv) had at least two non-parental adults who took genuine interest in them; (v) felt supported by friends; (vi) felt a sense of belonging in middle and high school; and (vii) enjoyed participating in community traditions. Although these questions were asked in Japanese, and some were modified from the original questions (the addition of "middle" in item 6), these seven items can be considered almost identical to the PCEs items used by Bethell et al.[10] Of these items, the first three were positioned as components of FPCEs and the others as CPCEs (Supplementary Table S1). The applicable items were summed to obtain the FPCE (0–3) and CPCE (0–4) scores. Like the PCEs scale[10], principal component analyses revealed a single-component structure for each FPCEs and CPCEs scale. Cronbach's alpha was 0.81 and 0.75 for FPCEs and CPCEs, respectively (Supplementary Table S2).

Adult health outcomes

Participants answered whether they currently had the following diseases using a dichotomous response variable (yes/no): (i) diabetes, (ii) ischemic heart disease (angina pectoris or myocardial infarction), (iii) stroke (cerebral infarction or hemorrhage), (iv) chronic obstructive pulmonary disease (COPD), (v) cancer, (vi) chronic pain (e.g., back pain and headache lasting ≥3 months), and (vii) depression. Additionally, (viii) suicidal ideation was determined as a "yes" response to the question, "Have you had times when you have wanted to die in the last year?" Psychological distress was measured with the Japanese version[29] of the Kessler 6-item psychological distress scale (K6)[30], assessing depressed mood and anxiety. The total score ranged from 0 to 24 on a five-point scale (0–4). A score of ≥13 was considered indicative of

For peer review only - http://bmjopen.bmj.com/site/about/guidelines.xhtml

(ix) serious psychological distress[31].

Covariates

The following background variables were included as covariates, in line with previous studies[1,13,32]: age, sex, and educational attainment.

For peer review only

Statistical analysis

Demographic characteristics and prevalence rates of all health outcomes in the sample were examined. For H1, multivariable logistic regression analyses were conducted to consider the direct effect of CPCEs on adult diseases after adjusting for covariates, ACEs, and FPCEs. For H2, multiple logistic regression analyses, stratified by CPCE exposure levels, were performed to examine whether CPCEs moderated the link between ACEs and adult diseases, adjusting for covariates and FPCEs. The extent to which CPCEs reduced the risk of each disease was also estimated.

The survey data were weighted to represent the Japanese population. Considering the possibility of multicollinearity, the variance inflation factor for each explanatory variable was checked to ensure that it did not exceed 4.0[33]. Stata 17.0 MP (StataCorp LLC, Texas, USA) was used for all analyses.

RESULTS

Participant characteristics

For peer review only - http://bmjopen.bmj.com/site/about/guidelines.xhtml

Table 1 shows that chronic pain and COPD were the most (18.5%) and least (1.4%) prevalent of the nine health outcomes, respectively. Approximately 39.9% of the participants had experienced ACEs. Furthermore, 52.6% and 55.8% of participants had experienced one or more FPCEs and CPCEs, respectively. When ACEs were divided into four exposure levels[9][10], as ACE exposure increased, the prevalence of adult diseases increased, the exposure rates to the two types of PCEs decreased, and the proportion of females and those with lower education levels increased (Supplementary Table S3). Finally, dose-response relationships between ACE exposure levels and all adult diseases were observed (Supplementary Table S4).

Table 1 Demographic characteristics and prevalence of health outcomes, ACEs, and PCEs among participants (N = 28254)

	**N**	**W %/M (SD)**
Health outcomes		
Diabetes	1872	7.3
Ischemic heart disease	661	2.9
Stroke	428	1.7
COPD	377	1.4
Cancer	683	2.6
Chronic pain	4967	18.5
Depression	1377	4.7
Suicidal ideation	3901	14.6
Severe psychological distress	2876	10.5
ACEs (0–11)	28254	0.82 (1.4)
0 ACEs	17662	60.1
≥1 ACEs	10592	49.9
FPCEs (0–3)	28254	1.13 (1.2)
0 FPCE	11914	47.4
1 FPCEs	4081	30.1
2 FPCEs	4943	22.5
3 FPCEs	7316	25.9
CPCEs (0–4)	28254	1.25 (1.4)

For peer review only - http://bmjopen.bmj.com/site/about/guidelines.xhtml

0 CPCE	10951	44.2
1 CPCEs	5467	19.4
2 CPCEs	4436	14.7
3 CPCEs	3860	11.4
4 CPCEs	3540	10.4
Age (19–79 years)	28254	47.6 (16.9)
Sex		
Male	13793	48.9
Female	14461	51.1
Educational attainment		
Less than HS	477	4.3
HS graduate	10344	60.3
Post-secondary and graduate	17223	34.0

W, weighted; SD, standard deviation; ACEs, adverse childhood experiences; CPCEs, community positive childhood experiences; FPCEs, family positive childhood experiences; HS, high school

Health outcomes according to ACE, FPCE, and CPCE exposure

Table 2 shows the adjusted odds ratios (AORs) for the poor health associated with CPCEs. After adjusting for covariates, ACEs, and FPCEs, CPCEs had significant negative direct effects on six adult diseases: stroke, COPD, chronic pain, depression, suicidal ideation, and severe psychological distress, supporting H1. For example, each additional CPCE decreased the adjusted odds of stroke by 16%. The same trend was found when CPCEs were treated as categorical variables (Supplementary Table S5). Overall, CPCEs may reduce the risk of mental illness.

Table 2 Adjusted odds ratios of health outcomes by ACEs (≥1 vs. 0), FPCEs, and CPCEs

	ACEs		FPCEs		CPCEs	
	**AOR***	**(95% CI)**	**AOR†**	**(95% CI)**	**AOR‡**	**(95% CI)**
Diabetes	**1.32**	(1.19–1.44)	**0.93**	(0.88–0.98)	0.98	(0.93–1.03)

For peer review only - http://bmjopen.bmj.com/site/about/guidelines.xhtml

Ischemic heart disease	**1.61**	(1.37–1.88)	**0.88**	(0.81–0.96)	0.93	(0.86–1.01)
Stroke	**2.00**	(1.64–2.44)	**0.83**	(0.74–0.92)	**0.84**	(0.77–0.93)
COPD	**2.05**	(1.66–2.54)	**0.77**	(0.69–0.86)	**0.88**	(0.80–0.98)
Cancer	**1.63**	(1.40–1.91)	**0.89**	(0.82–0.96)	0.94	(0.88–1.01)
Chronic pain	**1.73**	(1.62–1.84)	0.98	(0.95–1.01)	**0.92**	(0.89–0.95)
Depression	**2.86**	(2.55–3.22)	**0.89**	(0.83–0.95)	**0.81**	(0.76–0.86)
Suicidal ideation	**2.12**	(1.97–2.28)	**0.85**	(0.81–0.88)	**0.76**	(0.74–0.79)
Severe psychological distress	**2.28**	(2.10–2.48)	**0.85**	(0.81–0.90)	**0.72**	(0.69–0.75)

N=28254; Significance level of data in bold font <0.05.

*Adjusted for age, sex, educational attainment, FPCEs, and CPCEs.

†Adjusted for age, sex, educational attainment, ACEs, and CPCEs.

‡Adjusted for age, sex, educational attainment, ACEs, and FPCEs.

ACEs, adverse childhood experiences; FPCEs, family positive childhood experiences; CPCEs, community positive childhood experiences; AOR, adjusted odds ratio; CI, confidence interval

Health outcomes of ACEs across CPCE exposure levels

As shown in Figure 1, the AORs declined as PCE exposure levels increased; the cells become greener to the right. Particularly, the AORs tended to be smaller when the CPCE scores were ≥3 than when they were 0–2. Therefore, the interaction effects of ACEs and CPCEs on all nine outcomes were examined (Supplementary Table S6). The rightmost column of Figure 1 shows that the interaction effects between any ACE and ≥3 CPCEs were significant at the 10% level for seven diseases: ischemic heart disease, COPD, cancer, chronic pain, depression, suicidal ideation, and severe psychological distress.

Figure 2 shows the predicted values for each disease obtained from the regression equations. In support of H2, ≥3 CPCEs moderated the association between ACEs and adult diseases. Specifically, the predicted prevalence of ischemic heart disease did not differ according to CPCE exposure among those without ACEs; however, among those with ACEs, it decreased from 3.4% in those with 0–2 CPCEs to 1.6% in those with ≥3 CPCEs (relative risk: 47.2%).

For peer review only - http://bmjopen.bmj.com/site/about/guidelines.xhtml

Of the seven diseases in the previous paragraph, the adjusted prevalence was reduced by 50% or more for ischemic heart disease (52.8%), COPD (67.3%), depression (50.0%), and severe psychological distress (54.7%).

DISCUSSION

This study examined the associations among ACEs, CPCEs, and adult diseases using a population-based sample from Japan. Specifically, ACEs were associated with adult diseases, while CPCEs directly affected adult illnesses as a promotive factor and mitigated the adverse health effects of ACEs as a protective factor. The protective effect of CPCEs was particularly pronounced in ischemic heart disease, COPD, depression, and severe psychological distress, with relative risk reductions of 50% or more by three or more CPCEs in those with ACEs.

Consistent with earlier research[1,4,6], the results showed a dose-response connection between ACEs and all adult diseases evaluated. Although an association between ACEs and diseases during old age among Japanese individuals was previously reported[22], this appeared robust across a wide age range (18–79 years).

H1, based on the Compensatory Model, was supported for many of the diseases assessed in this study. CPCEs, independent of ACEs and FPCEs, directly affected adult-onset illnesses (i.e., stroke, COPD, chronic pain, depression, suicidal ideation, and severe psychological distress). While previous studies have found direct effects of PCEs primarily on mental health[10,11,13], the present study adds a novel finding that CPCEs independently function as promotive factors for physical and mental health.

More importantly, this study found analytical support for H2 based on the Protective Model.

For peer review only - http://bmjopen.bmj.com/site/about/guidelines.xhtml

CPCEs moderated the relationships between ACEs and adult diseases (i.e., ischemic heart disease, COPD, cancer, chronic pain, depression, suicidal ideation, and severe psychological distress), even after adjusting for FPCEs. Although the possible protective function of PCEs has been noted[9,10,12], the buffering effect may be due to family factors. Notably, the present study observed that CPCEs can independently play a protective role. Furthermore, extending research focusing on community assets moderating the association between ACEs and poor child health[15], this study suggests that the moderating effects of CPCEs may spread to adulthood, indicating that the protective effect may be stronger in cumulative situations where three or more CPCEs are aligned. The relative risk reduction of physical illness (i.e., ischemic heart disease and COPD) and mental illness (i.e., depression and severe psychological distress) in ACE survivors by more than half due to three or more CPCEs would be a non-negligible value. Whether this cutoff point is appropriate requires follow-up testing; however, the harm caused by ACEs may be mitigated in multiple relationships. Moreover, the analysis yielded the surprising finding that, for several diseases (i.e., ischemic heart disease and COPD), those with ACEs had lower prevalence rates than those without ACEs in situations of CPCE abundance (Figure 2). Perhaps, as the Challenge Model[24] of the Resilience Theory implies, moderate ACEs promote health when sufficient protective factors exist; that is, they act as immunization. Future studies should examine the generalizability of this phenomenon.

Why might CPCEs contribute to developing children and youth into healthier adults? Prior research argues that supportive adults other than parents may provide opportunities for children to elude life stressors, establish positive experiences in social connections, and feel a sense of having a place and social association[34]. Additionally, supportive close friendships promote resilience by developing constructive coping styles, encouraging efforts, providing supportive peer networks, and reducing inappropriate coping[35]. In interactions with non-family members at school and in the neighborhood, children exposed to ACEs may gain physical

For peer review only - http://bmjopen.bmj.com/site/about/guidelines.xhtml

refuge from toxic stress and develop psychological and instrumental coping strategies. Future studies need to explore the mechanisms linking CPCEs to resilience.

Strengths and limitations

To the best of our knowledge, this is the first study to prove that CPCEs could be promotive and protective factors of health system resilience. A strength of this study is the use of a large and representative sample. Moreover, various indicators elucidated the complex relationships among ACEs, PCEs, and adult diseases.

However, this study had some limitations. First, the data were self-reported and retrospective, which could have been influenced by recall bias. Individuals with a disease may be more likely to recall ACEs than healthy individuals. However, asking participants to reflect on their growing environment from a balanced perspective by asking about PCEs may have reduced this recall bias. Second, because of the study's cross-sectional nature, the possibility of reverse causation between ACEs/PCEs and adult illnesses cannot be eliminated. Third, the CPCEs measure used in this study did not include all CPCEs. Other types of CPCEs, such as participation in non-traditional activities and self-realization experiences, should be included in developing the CPCEs scale. Fourth, the extent of the positive effects of the CPCEs differed for each health outcome. Particularly, the role of CPCEs in mental illness was found to be robust; however, the validity of these effects should be carefully investigated by including other health outcomes.

Policy, practice, and research implications

For peer review only - http://bmjopen.bmj.com/site/about/guidelines.xhtml

The findings suggest that community-based interventions can be effective in helping children who have experienced adversity become resilient adults. Public health, social welfare, and educational practices that enforce CPCEs should be promoted. Additionally, the content of specific community-level approaches depends on social and local contexts. Such interventions should incorporate the main components of the Healthy Outcomes from Positive Experiences framework: (i) supportive and nurturing relationships, (ii) safe and stable environments, (iii) opportunities for constructive social involvement and connection, and (iv) social and emotional capacities[36]. For example, The National Resource Center at Tufts Medical Center[37] supports the above framework, providing tools and training to help healthcare providers, social service providers, educators, and members of community-based organizations acquire essential knowledge and skills to integrate these elements into their work.

In Japan, school counselors[38] and school social workers[39] in public elementary and junior high schools are expected to become resilience resources under compulsory education. Additionally, teachers need training in PCEs and improved working environments to build trusting relationships with their students. In local communities, traditional events such as festivals, nature experiences, multigenerational exchange programs, and the recently popularized “*Kodomo Shokudo*” (cafeterias for children)[40] could also contribute to the children’s resilience. Further research and practice should explore effective approaches to promoting CPCEs, including historical and new engagements.

Conclusion

This study showed that CPCEs positively impacted adult health in the general population regardless of ACE exposure and mitigated the adverse consequences. The study demonstrates

For peer review only - http://bmjopen.bmj.com/site/about/guidelines.xhtml

that CPCEs may contribute to the lifelong health status of ACE survivors. Therefore, further research is needed to investigate the impact mechanisms of CPCEs and improve the scale's accuracy, as well as interventions to strengthen supportive communities.

Contributors: HM and AA conceptualized the study. HM conducted the analysis and drafted the first manuscript. NK and AA contributed intellectual content to the study design and interpretation of the findings. TT collected data, managed the project, and obtained funding. All authors critically reviewed the manuscript and approved the final manuscript. HM is the guarantor for this study.

Funding: This study (JACSIS2022) was supported by the Japan Society for the Promotion of Science (JSPS) KAKENHI Grants (21H04856, 20K10467, 20K19633, 20K13721), the JST Grant (JPMJPF2017), the Health Labor Sciences Research Grant (21HA2016), the grant for 2021-2022 Strategic Research Promotion (SK202116) of Yokohama City University, and the research program on "Using Health Metrics to Monitor and Evaluate the Impact of Health Policies," conducted at the Tokyo Foundation for Policy Research. HM was also supported by the JSPS KAKENHI Grant (21K13454) and the Ministry of Education, Culture, Sports, Science and Technology (MEXT) KAKENHI Grant (20H05805).

Competing interests: None declared.

Data availability statement: All de-identified data from participants reported in this study are available to interested researchers who submit data-sharing requests with a summary of the secondary analysis plan to the last author.

For peer review only

For peer review only - http://bmjopen.bmj.com/site/about/guidelines.xhtml

ORCID iDs:

Haruyo Mitatani https://orcid.org/0000-0001-8882-5098

Naoki Kondo https://orcid.org/0000-0002-6425-6844

Airi Amemiya https://orcid.org/0000-0001-5319-9541

Takahiro Tabuchi http://orcid.org/0000-0002-1050-3125

REFERENCES

1 Felitti VJ, Anda RF, Nordenberg D, *et al.* Relationship of childhood abuse and household dysfunction to many of the leading causes of death in adults. The Adverse Childhood Experiences (ACE) Study. *Am J Prev Med* 1998;**14**:245–58. doi:10.1016/s0749-3797(98)00017-8

2 Anda RF, Felitti VJ, Bremner JD, *et al.* The enduring effects of abuse and related adverse experiences in childhood: a convergence of evidence from neurobiology and epidemiology. *Eur Arch Psychiatry Clin Neurosci* 2006;**256**:174–86. doi:10.1007/s00406-005-0624-4

3 Bellis MA, Hughes K, Ford K, *et al.* Life course health consequences and associated annual costs of adverse childhood experiences across Europe and North America: a systematic review and meta-analysis. *Lancet Public Heal* 2019;**4**:e517–28. doi:10.1016/S2468-2667(19)30145-8

4 Hughes K, Bellis MA, Hardcastle, A K, *et al.* The effect of multiple adverse childhood experiences on health: a systematic review and meta-analysis. *Lancet Public Heal* 2017;**2**:e356–66.https://doi.org/10.1016/S2468-2667(17)30118-4.

5 Norman RE, Byambaa M, De R, *et al.* The long-term health consequences of child physical abuse, emotional abuse, and neglect: a systematic review and meta-analysis. *Plos Med* 2012;**9**:1–31. doi:10.1371/journal.pmed.1001349

6 Petruccelli K, Davis J, Berman T. Adverse childhood experiences and associated health outcomes: a systematic review and meta-analysis. *Child Abus Negl* 2019;**97**:104127. doi:10.1016/j.chiabu.2019.104127

7 Shonkoff JP, Garner AS, Siegel BS, *et al.* The lifelong effects of early childhood adversity and toxic stress. *Pediatrics* 2012;**129**:e232–46. doi:10.1542/PEDS.2011-

For peer review only

For peer review only - http://bmjopen.bmj.com/site/about/guidelines.xhtml

2663

8 Oral R, Ramirez M, Coohey C, *et al.* Adverse childhood experiences and trauma informed care: the future of health care. *Pediatr Res* 2016;**79**:227–33. doi:10.1038/pr.2015.197

9 Bellis MA, Hardcastle K, Ford K, *et al.* Does continuous trusted adult support in childhood impart life-course resilience against adverse childhood experiences - a retrospective study on adult health-harming behaviours and mental well-being. *BMC Psychiatry* 2017;**17**:110. doi:10.1186/S12888-017-1260-Z/TABLES/3

10 Bethell C, Jones J, Gombojav N, *et al.* Positive childhood experiences and adult mental and relational health in a statewide sample: associations across adverse childhood experiences levels. *JAMA Pediatr* 2019;**173**:e193007. doi:10.1001/jamapediatrics.2019.3007

11 Crandall AA, Miller JR, Cheung A, *et al.* ACEs and counter-ACEs: how positive and negative childhood experiences influence adult health. *Child Abuse Negl* 2019;**96**:104089. doi:10.1016/J.CHIABU.2019.104089

12 Crouch E, Radcliff E, Strompolis M, *et al.* Safe, stable, and nurtured: protective factors against poor physical and mental health outcomes following exposure to adverse childhood experiences (ACEs). *J Child Adolesc Trauma* 2019;**12**:165–73. doi:10.1007/S40653-018-0217-9/METRICS

13 Kuhar M, Zager Kocjan G. Associations of adverse and positive childhood experiences with adult physical and mental health and risk behaviours in Slovenia. *Eur J Psychotraumatol* 2021;**12**:1924953. doi:10.1080/20008198.2021.1924953

14 Narayan AJ, Rivera LM, Bernstein RE, *et al.* Positive childhood experiences predict less psychopathology and stress in pregnant women with childhood adversity: a pilot study of the benevolent childhood experiences (BCEs) scale. *Child Abuse Negl* 2018;**78**:19–30. doi:10.1016/J.CHIABU.2017.09.022

15 Bellis MA, Hughes K, Ford K, *et al.* Adverse childhood experiences and sources of childhood resilience: a retrospective study of their combined relationships with child health and educational attendance. *BMC Public Health* 2018;**18**:1–12. doi:10.1186/S12889-018-5699-8/FIGURES/1

16 Masten AS, Cicchetti D. Resilience in development: progress and transformation. In: D. Cicchetti, ed. *Developmental Psychopathology, Risk, Resilience, and Intervention*.

For peer review only - http://bmjopen.bmj.com/site/about/guidelines.xhtml

John Wiley & Sons, Inc. 2016. 271–333. doi:https://doi.org/10.1002/9781119125556.devpsy406

17 Gartland D, Riggs E, Muyeen S, *et al.* What factors are associated with resilient outcomes in children exposed to social adversity? a systematic review. *BMJ Open* 2019;**9**:e024870. doi:10.1136/BMJOPEN-2018-024870

18 Henson M, Sabo S, Trujillo A, *et al.* Identifying protective factors to promote health in american indian and alaska native adolescents: a literature review. *J Prim Prev* 2017;**38**:5–26. doi:10.1007/S10935-016-0455-2/METRICS

19 Okwori G. Role of individual, family, and community resilience in moderating effects of adverse childhood experiences on mental health among children. *J Dev Behav Pediatr* 2022;**43**:E452–62. doi:10.1097/DBP.0000000000001076

20 Molloy C, Beatson R, Harrop C, *et al.* Systematic review: effects of sustained nurse home visiting programs for disadvantaged mothers and children. *J Adv Nurs* 2021;**77**:147–61. doi:10.1111/JAN.14576

21 Isumi A, Doi S, Ochi M, *et al.* School- and community-level protective factors for resilience among chronically maltreated children in Japan. *Soc Psychiatry Psychiatr Epidemiol* 2023;**58**:477–88. doi:10.1007/S00127-022-02322-X/TABLES/5

22 Amemiya A, Fujiwara T, Shirai K, *et al.* Association between adverse childhood experiences and adult diseases in older adults: a comparative cross-sectional study in Japan and Finland. *BMJ Open* 2019;**9**:e024609. doi:10.1136/bmjopen-2018-024609

23 Fujiwara T, Kawakami N. Association of childhood adversities with the first onset of mental disorders in Japan: results from the World Mental Health Japan, 2002-2004. *J Psychiatr Res* 2011;**45**:481–7. doi:10.1016/j.jpsychires.2010.08.002

24 Zimmerman MA. Resiliency theory: a strengths-based approach to research and practice for adolescent health. *Heal Educ Behav* 2013;**40**:381–3. doi:10.1177/1090198113493782

25 Oshri A, Duprey EK, Liu S, *et al.* ACEs and resilience: methodological and conceptual issues. In: Asmundson GJG, Afifi TO, eds. *Adverse Childhood Experiences: Using Evidence to Advance Research, Practice, Policy, and Prevention*. Academic Press 2020. 287–306.

26 JACSIS study. The Japan COVID-19 and Society Internet Survey. 2021.https://jacsis-

For peer review only

For peer review only - http://bmjopen.bmj.com/site/about/guidelines.xhtml

study.jp/

27 Fujiwara T. Impact of adverse childhood experience on physical and mental health: a life-course epidemiology perspective. *Psychiatry Clin Neurosci* 2022;**76**:544–51. doi:10.1111/PCN.13464

28 Cheong E Von, Sinnott C, Dahly D, *et al.* Adverse childhood experiences (ACEs) and later-life depression: perceived social support as a potential protective factor. *BMJ Open* 2017;**7**:e013228. doi:10.1136/BMJOPEN-2016-013228

29 Furukawa TA, Kawakami N, Saitoh M, *et al.* The performance of the Japanese version of the K6 and K10 in the World Mental Health Survey Japan. *Int J Methods Psychiatr Res* 2008;**17**:152–8. doi:10.1002/mpr.257

30 Kessler RC, Andrews G, Colpe LJ, *et al.* Short screening scales to monitor population prevalences and trends in non-specific psychological distress. *Psychol Med* 2002;**32**:959–76. doi:10.1017/S0033291702006074

31 Kessler RC, Green JG, Gruber MJ, *et al.* Screening for serious mental illness in the general population with the K6 screening scale: results from the WHO World Mental Health (WMH) survey initiative. *Int J Methods Psychiatr Res* 2010;**19**:4–22. doi:10.1002/mpr.310

32 Metzler M, Merrick MT, Klevens J, *et al.* Adverse childhood experiences and life opportunities: shifting the narrative. *Child Youth Serv Rev* 2017;**72**:141–9. doi:10.1016/j.childyouth.2016.10.021

33 Hair JF, Black B, Babin BJ, *et al. Multivariate Data Analysis: Global Edition*. 7th ed. Pearson Education 2009.

34 Rhodes JE, Spencer R, Keller TE, *et al.* A model for the influence of mentoring relationships on youth development. *J Community Psychol* 2006;**34**:691–707. doi:10.1002/JCOP.20124

35 Graber R, Turner R, Madill A. Best friends and better coping: facilitating psychological resilience through boys' and girls' closest friendships. *Br J Psychol* 2016;**107**:338–58. doi:10.1111/BJOP.12135

36 Sege RD, Harper Browne C. Responding to ACEs with HOPE: Health Outcomes from Positive Experiences. *Acad Pediatr* 2017;**17**:S79–85. doi:10.1016/J.ACAP.2017.03.007

For peer review only

For peer review only - http://bmjopen.bmj.com/site/about/guidelines.xhtml

37 The National Resource Center at Tufts Medical Center. Tufts HOPE: Healthy Outcomes from Positive Experiences. 2023.https://positiveexperience.org/

38 Nishio A, Kakimoto M, Horita R, *et al.* Compulsory educational mental health support system in Japan. *Pediatr Int* 2020;**62**:529–34. doi:10.1111/PED.14205

39 Kanazawa M. Current issues of school social work in Japan : from the historical relations between school, social work, and counseling. *Japanese J Soc Serv* 2009;**5**:159–71. doi:10.24469/JJSWE.5.0_159

40 Murayama H, Kurotani K, Tabuchi T. Social capital and the spread of children's cafeterias (kodomo shokudo) in Japan: an ecological analysis. *Asia-Pacific J public Heal* 2022;**34**:817–20. doi:10.1177/10105395221119416

Figure legends

Figure 1

A heatmap of the adjusted odds ratios with 95% CIs for the health outcomes of ACEs (≥1 vs. 0) across CPCE exposure levels. All odds ratios are adjusted for age, sex, educational attainment, and FPCEs. The significance level of data in bold font is <0.05. *P value for the interaction effect of ACEs (≥1 vs. 0) × CPCEs (cumulative variable). †P value for interaction effect of ACEs (≥1 vs. 0) × CPCEs (≥3+ vs. 0–2).

Figure 2

Adjusted predictions with 95% CIs for the health outcomes of ACEs (≥1 vs. 0) × CPCEs (≥3 vs. 0–2). Substitutions were performed on the multiple logistic regression models presented in Supplementary Table S6.

For peer review only - http://bmjopen.bmj.com/site/about/guidelines.xhtml

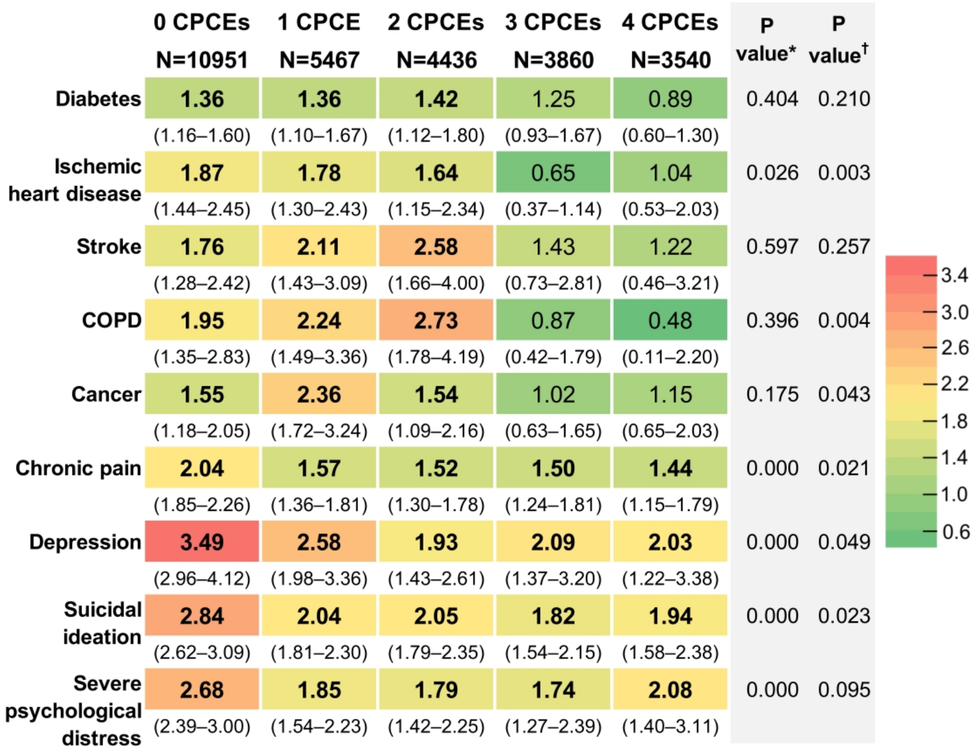

Figure 1

A heatmap of the adjusted odds ratios with 95% CIs for the health outcomes of ACEs (≥1 vs. 0) across CPCE exposure levels. All odds ratios are adjusted for age, sex, educational attainment, and FPCEs. The significance level of data in bold font is <0.05. *P value for the interaction effect of ACEs (≥1 vs. 0) × CPCEs (cumulative variable). †P value for interaction effect of ACEs (≥1 vs. 0) × CPCEs (≥3+ vs. 0–2).

130x102mm (1200 x 1200 DPI)

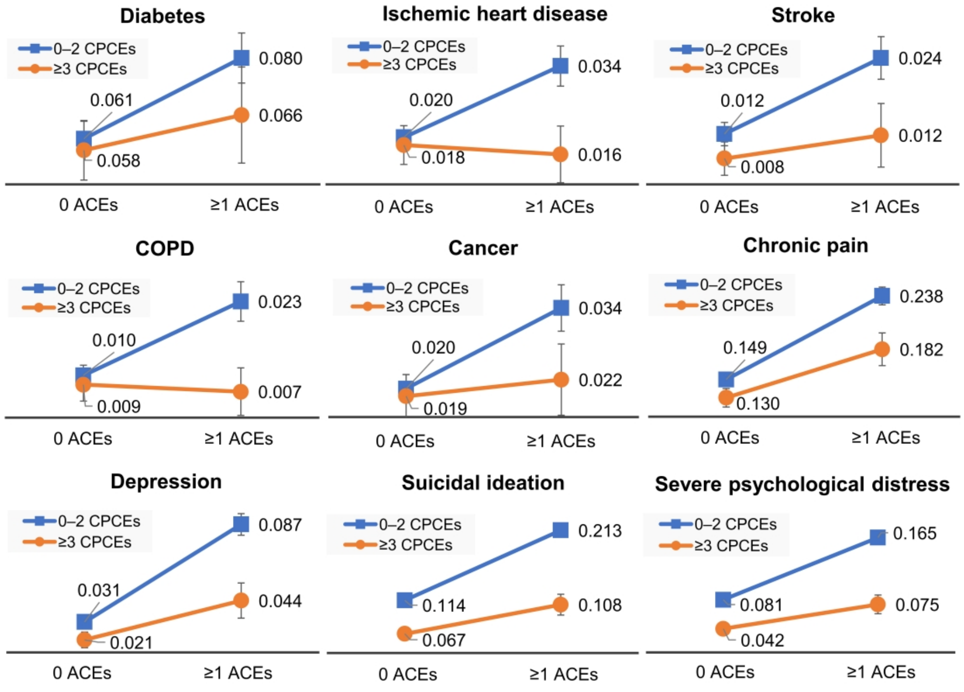

Figure 2

Adjusted predictions with 95% CIs for the health outcomes of ACEs (≥1 vs. 0) × CPCEs (≥3 vs. 0–2). Substitutions were performed on the multiple logistic regression models presented in Supplementary Table S6.

130x92mm (1200 x 1200 DPI)

Supplemental Material

Table S1 Prevalence of PCE single items among participants (N=28254)

	N	weighted %
FPCEs		
i) Felt able to talk to their family about feelings	8957	28.4
ii) Felt their family stood by them during difficult times	12508	39.7
iii) Felt safe and protected by an adult in their home	14450	45.5
CPCEs		
iv) Had at least 2 nonparent adults who took genuine interest in them	10558	32.7
v) Felt supported by friends	10429	32.1
vi) Felt a sense of belonging in middle and high school	12128	37.7
vii) Enjoyed participating in community traditions	6964	22.0

FPCEs, family positive childhood experiences; CPCEs, community positive childhood experiences

Table S2 Summary of results of psychometric analyses performed on FPCEs and CPCEs

	Internal Consistency (Cronbach's alpha)	Principle components factor analysis results	Factor loading across items
FPCEs	0.81	A single Eigen value greater than 1.0 (2.19) that explained 72.9% of variance	0.54 ("felt able to talk") to 0.61 ("felt safe and protected")
CPCEs	0.75	A single Eigen value greater than 1.0 (2.28) that explained 56.9% of variance	0.46 ("community traditions") to 0.52 ("felt supported by friends")

FPCEs, family positive childhood experiences; CPCEs, community positive childhood experiences

For peer review only

Table S3 Demographic characteristics and prevalence of health outcomes, PCEs by ACE exposure levels

	Prevalence of ACEs				
	0 ACEs	1 ACE	2–3 ACEs	≥4 ACEs	
	N=17662	N=5443	N=3701	N=1448	
	W %/M	W %/M	W %/M	W %/M	P value*
Health outcomes					
Diabetes	6.5	8.7	7.6	10.5	<0.001
Ischemic heart disease	2.5	2.7	3.0	6.8	<0.001
Stroke	1.2	1.4	2.3	5.6	<0.001
COPD	1.0	0.8	2.2	5.7	<0.001
Cancer	2.0	2.7	3.3	6.8	<0.001
Chronic pain	14.5	20.8	26.6	33.2	<0.001
Depression	2.8	4.0	8.6	17.7	<0.001
Suicidal ideation	10.5	13.3	25.8	35.7	<0.001
Severe psychological distress	7.1	9.1	19.0	30.6	<0.001
FPCEs (0–3)	1.3	1.1	0.7	0.3	<0.001
0 FPCE	40.1	48.0	65.1	80.2	<0.001
1 FPCEs	14.5	13.7	14.0	10.7	0.095
2 FPCEs	17.8	17.1	11.7	5.4	<0.001
3 FPCEs	27.7	21.3	9.3	3.7	<0.001
CPCEs (0–4)	1.4	1.2	0.9	0.7	<0.001
0 CPCE	40.6	44.0	52.9	61.8	<0.001
1 CPCEs	18.4	21.0	21.8	18.6	<0.001
2 CPCEs	14.9	15.7	12.9	12.7	0.003
3 CPCEs	13.1	10.5	7.9	4.4	<0.001
4 CPCEs	13.1	8.8	4.5	2.6	<0.001
Age (19–79)	46.9	50.7	47.0	45.2	<0.001
Sex					
Male	52.9	46.2	39.8	38.5	<0.001
Female	47.1	53.8	60.2	61.5	<0.001
Educational attainment					
Less than HS	2.7	7.3	5.0	9.1	<0.001
HS graduate	57.8	62.7	64.9	66.7	<0.001
Post-secondary and graduate	37.9	28.9	28.6	23.1	<0.001

*P value for significant differences by Pearson's χ^2 test or ANOVA.

W, weighted; ACEs, adverse childhood experiences; FPCEs, family positive childhood experiences; CPCEs, community positive childhood experiences; HS, high school

For peer review only

Table S4 Adjusted odds ratios of health outcomes by ACE exposure levels

	Diabetes		Ischemic heart disease		Stroke		COPD		Cancer		Chronic pain		Depression		Suicidal ideation		Severe psychological distress	
	**AOR**	**(95% CI)**	**AOR**	**(95% CI)**	**AOR**	**(95% CI)**	**AOR**	**(95% CI)**	**AOR**	**(95% CI)**	**AOR**	**(95% CI)**	**AOR**	**(95% CI)**	**AOR**	**(95% CI)**	**AOR**	**(95% CI)**
ACEs																		
0 ACEs [reference]	1.00		1.00		1.00		1.00		1.00		1.00		1.00		1.00		1.00	
1 ACE	1.16	(1.02–1.31)	1.11	(0.90–1.38)	1.28	(0.97–1.68)	1.01	(0.73–1.39)	1.03	(0.83–1.27)	1.43	(1.32–1.55)	1.75	(1.50–2.05)	1.65	(1.50–1.81)	1.66	(1.49–1.85)
2–3 ACEs	1.46	(1.26–1.68)	1.88	(1.51–2.33)	2.32	(1.79–3.01)	2.68	(2.05–3.49)	1.95	(1.58–2.39)	1.95	(1.78–2.12)	3.80	(3.29–4.38)	3.15	(2.87–3.46)	3.22	(2.90–3.58)
≥4 ACEs	2.03	(1.66–2.48)	3.70	(2.87–4.77)	5.29	(4.00–7.00)	5.79	(4.33–7.74)	3.77	(2.95–4.82)	2.97	(2.63–3.34)	8.11	(6.89–9.55)	4.28	(3.78–4.86)	5.93	(5.20–6.77)
Age	1.04	(1.04–1.05)	1.02	(1.01–1.02)	0.99	(0.99–1.00)	0.98	(0.97–0.99)	1.02	(1.02–1.03)	1.01	(1.01–1.01)	0.98	(0.98–0.98)	0.96	(0.96–0.97)	0.96	(0.96–0.96)
Sex																		
Male [reference]	1.00		1.00		1.00		1.00		1.00		1.00		1.00		1.00		1.00	
Female	0.31	(0.27–0.34)	0.33	(0.28–0.39)	0.39	(0.32–0.48)	0.32	(0.26–0.40)	0.73	(0.63–0.85)	1.13	(1.06–1.21)	0.62	(0.55–0.69)	1.01	(0.94–1.09)	0.87	(0.81–0.95)
Educational attainment																		
Less than HS	1.63	(1.21–2.21)	1.18	(0.72–1.96)	1.01	(0.53–1.93)	1.24	(0.66–2.31)	0.79	(0.45–1.39)	1.24	(0.99–1.54)	1.62	(1.18–2.22)	1.68	(1.34–2.11)	1.56	(1.21–2.01)
HS graduate	1.14	(1.03–1.26)	1.03	(0.87–1.21)	1.04	(0.85–1.28)	0.99	(0.79–1.23)	0.82	(0.70–0.97)	1.11	(1.04–1.19)	1.12	(0.99–1.25)	1.08	(1.00–1.16)	1.02	(0.93–1.11)
Post-secondary and graduate [reference]	1.00		1.00		1.00		1.00		1.00		1.00		1.00		1.00		1.00	

AOR, adjusted odds ratio; ACEs, adverse childhood experiences; HS, high school

For peer review only

Table S5 Adjusted odds ratios of health outcomes by ACEs, FPCEs, and CPCEs

	Diabetes		Ischemic heart disease		Stroke		COPD		Cancer		Chronic pain		Depression		Suicidal ideation		Severe psychological distress	
	**AOR**	**(95% CI)**	**AOR**	**(95% CI)**	**AOR**	**(95% CI)**	**AOR**	**(95% CI)**	**AOR**	**(95% CI)**	**AOR**	**(95% CI)**	**AOR**	**(95% CI)**	**AOR**	**(95% CI)**	**AOR**	**(95% CI)**
ACEs																		
0 ACEs [reference]	1.00		1.00		1.00		1.00		1.00		1.00		1.00		1.00		1.00	
≥1 ACEs	1.32	(1.19–1.46)	1.60	(1.36–1.87)	1.96	(1.61–2.39)	2.01	(1.63–2.49)	1.62	(1.38–1.89)	1.73	(1.62–1.84)	2.86	(2.55–3.22)	2.13	(1.98–2.29)	2.29	(2.10–2.49)
FPCEs																		
0–1 FPCEs [reference]	1.00		1.00		1.00		1.00		1.00		1.00		1.00		1.00		1.00	
2 FPCEs	0.96	(0.83–1.11)	0.97	(0.78–1.21)	0.73	(0.55–0.97)	0.72	(0.55–0.96)	0.84	(0.67–1.04)	0.99	(0.90–1.09)	0.88	(0.73–1.05)	0.84	(0.75–0.94)	0.83	(0.73–0.95)
3 FPCEs	0.81	(0.69–0.94)	0.64	(0.50–0.82)	0.45	(0.33–0.63)	0.36	(0.25–0.51)	0.63	(0.50–0.79)	0.93	(0.85–1.03)	0.67	(0.55–0.82)	0.62	(0.55–0.70)	0.66	(0.57–0.76)
CPCEs																		
0–1 CPCEs [reference]	1.00		1.00		1.00		1.00		1.00		1.00		1.00		1.00		1.00	
2–3 CPCEs	1.06	(0.93–1.20)	1.03	(0.85–1.26)	0.93	(0.73–1.19)	1.19	(0.94–1.52)	1.04	(0.86–1.26)	0.89	(0.82–0.97)	0.74	(0.63–0.87)	0.66	(0.60–0.73)	0.56	(0.50–0.63)
4 CPCEs	0.83	(0.68–1.01)	0.70	(0.50–0.99)	0.53	(0.33–0.86)	0.46	(0.27–0.81)	0.84	(0.62–1.13)	0.75	(0.66–0.85)	0.54	(0.41–0.71)	0.41	(0.35–0.49)	0.34	(0.28–0.42)
Age	1.04	(1.04–1.04)	1.02	(1.01–1.02)	0.99	(0.98–1.00)	0.98	(0.97–0.98)	1.02	(1.01–1.02)	1.01	(1.01–1.01)	0.98	(0.98–0.98)	0.96	(0.96–0.96)	0.96	(0.95–0.96)
Sex																		
Male [reference]	1.00		1.00		1.00		1.00		1.00		1.00		1.00		1.00		1.00	
Female	0.31	(0.28–0.35)	0.35	(0.29–0.41)	0.42	(0.34–0.51)	0.34	(0.27–0.43)	0.75	(0.64–0.88)	1.17	(1.10–1.25)	0.70	(0.63–0.79)	1.17	(1.09–1.26)	1.03	(0.95–1.12)
Educational attainment																		
Less than HS	1.72	(1.27–2.33)	1.37	(0.83–2.26)	1.21	(0.64–2.31)	1.61	(0.87–3.00)	0.97	(0.55–1.71)	1.30	(1.05–1.62)	1.70	(1.25–2.31)	1.59	(1.27–2.00)	1.49	(1.16–1.91)
HS graduate	1.14	(1.03–1.26)	1.05	(0.89–1.24)	1.07	(0.88–1.32)	1.04	(0.84–1.29)	0.86	(0.73–1.01)	1.12	(1.05–1.20)	1.11	(0.98–1.24)	1.03	(0.95–1.11)	0.97	(0.89–1.06)
Post-secondary and graduate [reference]	1.00		1.00		1.00		1.00		1.00		1.00		1.00		1.00		1.00	

AOR, adjusted odds ratio; ACEs, adverse childhood experiences; FPCEs, family positive childhood experiences; CPCEs, community positive childhood; HS, high school

For peer review only

Table S6 Adjusted odds ratios of health outcomes by ACEs, CPCEs, and these interaction terms

	Diabetes		Ischemic heart disease		Stroke		COPD		Cancer		Chronic pain		Depression		Suicidal ideation		Severe psychological distress	
	**AOR**	**(95% CI)**	**AOR**	**(95% CI)**	**AOR**	**(95% CI)**	**AOR**	**(95% CI)**	**AOR**	**(95% CI)**	**AOR**	**(95% CI)**	**AOR**	**(95% CI)**	**AOR**	**(95% CI)**	**AOR**	**(95% CI)**
ACEs																		
0 ACEs [reference]	1.00		1.00		1.00		1.00		1.00		1.00		1.00		1.00		1.00	
≥1 ACEs	1.43	(1.29–1.58)	1.78	(1.49–2.12)	2.05	(1.66–2.53)	2.28	(1.82–2.86)	1.75	(1.46–2.08)	1.79	(1.67–1.92)	2.98	(2.63–3.38)	2.19	(2.03–2.37)	2.34	(2.14–2.56)
CPCEs																		
0–2 CPCEs [reference]	1.00		1.00		1.00		1.00		1.00		1.00		1.00		1.00		1.00	
≥3 CPCEs	0.92	(0.80–1.07)	0.92	(0.70–1.20)	0.67	(0.46–0.98)	0.84	(0.58–1.23)	0.93	(0.72–1.20)	0.85	(0.77–0.94)	0.66	(0.52–0.85)	0.54	(0.47–0.63)	0.48	(0.41–0.57)
Interaction																		
≥1 ACEs × ≥3 CPCEs	0.82	(0.65–1.03)	0.50	(0.32–0.79)	0.72	(0.40–1.27)	0.38	(0.19–0.74)	0.66	(0.45–0.99)	0.83	(0.71–0.97)	0.71	(0.51–1.00)	0.78	(0.64–0.97)	0.81	(0.63–1.04)
Age	1.04	(1.03–1.04)	1.02	(1.01–1.02)	0.99	(0.98–1.00)	0.98	(0.97–0.98)	1.02	(1.01–1.02)	1.01	(1.01–1.01)	0.98	(0.98–0.98)	0.96	(0.96–0.96)	0.96	(0.95–0.96)
Sex																		
Male [reference]	1.00		1.00		1.00		1.00		1.00		1.00		1.00		1.00		1.00	
Female	0.31	(0.28–0.35)	0.34	(0.29–0.41)	0.42	(0.34–0.51)	0.34	(0.27–0.43)	0.75	(0.64–0.88)	1.17	(1.10–1.24)	0.70	(0.62–0.78)	1.15	(1.07–1.24)	1.01	(0.93–1.09)
Educational attainment																		
Less than HS	1.50	(1.13–1.97)	1.33	(0.81–2.20)	1.18	(0.62–2.26)	1.53	(0.82–2.86)	0.95	(0.54–1.67)	1.29	(1.04–1.61)	1.73	(1.27–2.36)	1.62	(1.29–2.04)	1.55	(1.21–2.00)
HS graduate	1.09	(0.99–1.19)	1.05	(0.89–1.24)	1.07	(0.87–1.31)	1.03	(0.82–1.28)	0.86	(0.73–1.01)	1.12	(1.05–1.19)	1.11	(0.99–1.25)	1.04	(0.96–1.12)	0.98	(0.90–1.07)
Post-secondary and graduate [reference]	1.00		1.00		1.00		1.00		1.00		1.00		1.00		1.00		1.00	
FPCEs																		
0–1 FPCEs [reference]	1.00		1.00		1.00		1.00		1.00		1.00		1.00		1.00		1.00	
2 FPCEs	0.95	(0.83–1.08)	1.01	(0.81–1.25)	0.76	(0.57–1.00)	0.76	(0.57–1.01)	0.86	(0.70–1.07)	1.00	(0.91–1.10)	0.79	(0.67–0.93)	0.75	(0.68–0.83)	0.68	(0.60–0.76)
3 FPCEs	0.77	(0.67–0.88)	0.66	(0.52–0.85)	0.47	(0.34–0.65)	0.36	(0.25–0.53)	0.65	(0.52–0.82)	0.93	(0.85–1.03)	0.60	(0.50–0.73)	0.55	(0.49–0.61)	0.53	(0.46–0.60)

For peer review only

STROBE Statement—Checklist of items that should be included in reports of ***cross-sectional studies***

	Item No	Recommendation	Page No
**Title and abstract**	1	(*a*) Indicate the study's design with a commonly used term in the title or the abstract	1-2
		(*b*) Provide in the abstract an informative and balanced summary of what was done and what was found	2
**Introduction**			
Background/rationale	2	Explain the scientific background and rationale for the investigation being reported	4
Objectives	3	State specific objectives, including any prespecified hypotheses	5
**Methods**			
Study design	4	Present key elements of study design early in the paper	4-5
Setting	5	Describe the setting, locations, and relevant dates, including periods of recruitment, exposure, follow-up, and data collection	6
Participants	6	(*a*) Give the eligibility criteria, and the sources and methods of selection of participants	6
Variables	7	Clearly define all outcomes, exposures, predictors, potential confounders, and effect modifiers. Give diagnostic criteria, if applicable	7-9
Data sources/ measurement	8*	For each variable of interest, give sources of data and details of methods of assessment (measurement). Describe comparability of assessment methods if there is more than one group	*7-9*
Bias	9	Describe any efforts to address potential sources of bias	6
Study size	10	Explain how the study size was arrived at	6
Quantitative variables	11	Explain how quantitative variables were handled in the analyses. If applicable, describe which groupings were chosen and why	7-9
Statistical methods	12	(*a*) Describe all statistical methods, including those used to control for confounding	9
		(*b*) Describe any methods used to examine subgroups and interactions	9
		(*c*) Explain how missing data were addressed	6
		(*d*) If applicable, describe analytical methods taking account of sampling strategy	9
		(*e*) Describe any sensitivity analyses	9
**Results**			
Participants	13*	(a) Report numbers of individuals at each stage of study—eg numbers potentially eligible, examined for eligibility, confirmed eligible, included in the study, completing follow-up, and analysed	6
		(b) Give reasons for non-participation at each stage	6
		(c) Consider use of a flow diagram	6
Descriptive data	14*	(a) Give characteristics of study participants (eg demographic, clinical, social) and information on exposures and potential confounders	9-11
		(b) Indicate number of participants with missing data for each variable of interest	9-11
Outcome data	15*	Report numbers of outcome events or summary measures	10
Main results	16	(*a*) Give unadjusted estimates and, if applicable, confounder-adjusted estimates and their precision (eg, 95% confidence interval). Make clear which confounders were adjusted for and why they were included	11-13

For peer review only

For peer review only - http://bmjopen.bmj.com/site/about/guidelines.xhtml

		(*b*) Report category boundaries when continuous variables were categorized	10-12
		(*c*) If relevant, consider translating estimates of relative risk into absolute risk for a meaningful time period	11-13
Other analyses	17	Report other analyses done—eg analyses of subgroups and interactions, and sensitivity analyses	12-13
**Discussion**			
Key results	18	Summarise key results with reference to study objectives	13
Limitations	19	Discuss limitations of the study, taking into account sources of potential bias or imprecision. Discuss both direction and magnitude of any potential bias	15
Interpretation	20	Give a cautious overall interpretation of results considering objectives, limitations, multiplicity of analyses, results from similar studies, and other relevant evidence	13-15
Generalisability	21	Discuss the generalisability (external validity) of the study results	13-15
**Other information**			
Funding	22	Give the source of funding and the role of the funders for the present study and, if applicable, for the original study on which the present article is based	17

*Give information separately for exposed and unexposed groups.

Note: An Explanation and Elaboration article discusses each checklist item and gives methodological background and published examples of transparent reporting. The STROBE checklist is best used in conjunction with this article (freely available on the Web sites of PLoS Medicine at http://www.plosmedicine.org/, Annals of Internal Medicine at http://www.annals.org/, and Epidemiology at http://www.epidem.com/). Information on the STROBE Initiative is available at www.strobe-statement.org.

For peer review only

For peer review only - http://bmjopen.bmj.com/site/about/guidelines.xhtml

BMJ Open

Promotive and protective effects of community-related positive childhood experiences on adult health outcomes in the context of adverse childhood experiences: a nationwide cross-sectional survey in Japan

Journal:	*BMJ Open*
Manuscript ID	bmjopen-2023-082134.R1
Article Type:	Original research
Date Submitted by the Author:	09-Feb-2024
Complete List of Authors:	Mitani, Haruyo; Osaka University, Graduate School of Human Sciences<br>Kondo, Naoki; Kyoto University, Graduate School of Medicine and School of Public Health, Department of Social Epidemiology<br>Amemiya, Airi; Kyoto University, Graduate School of Medicine and School of Public Health, Department of Social Epidemiology (former affiliation)<br>Tabuchi, Takahiro; Osaka International Cancer Institute, Cancer Control Center
**Primary Subject Heading**:	Epidemiology
Secondary Subject Heading:	Communication, Epidemiology, Mental health, Sociology
Keywords:	Chronic Disease, EPIDEMIOLOGY, MENTAL HEALTH, Social Interaction, Stress, Psychological

SCHOLARONE™
Manuscripts

I, the Submitting Author has the right to grant and does grant on behalf of all authors of the Work (as defined in the below author licence), an exclusive licence and/or a non-exclusive licence for contributions from authors who are: i) UK Crown employees; ii) where BMJ has agreed a CC-BY licence shall apply, and/or iii) in accordance with the terms applicable for US Federal Government officers or employees acting as part of their official duties; on a worldwide, perpetual, irrevocable, royalty-free basis to BMJ Publishing Group Ltd ("BMJ") its licensees and where the relevant Journal is co-owned by BMJ to the co-owners of the Journal, to publish the Work in this journal and any other BMJ products and to exploit all rights, as set out in our licence.

The Submitting Author accepts and understands that any supply made under these terms is made by BMJ to the Submitting Author unless you are acting as an employee on behalf of your employer or a postgraduate student of an affiliated institution which is paying any applicable article publishing charge ("APC") for Open Access articles. Where the Submitting Author wishes to make the Work available on an Open Access basis (and intends to pay the relevant APC), the terms of reuse of such Open Access shall be governed by a Creative Commons licence – details of these licences and which Creative Commons licence will apply to this Work are set out in our licence referred to above.

Other than as permitted in any relevant BMJ Author's Self Archiving Policies, I confirm this Work has not been accepted for publication elsewhere, is not being considered for publication elsewhere and does not duplicate material already published. I confirm all authors consent to publication of this Work and authorise the granting of this licence.

For peer review only

Title

Promotive and protective effects of community-related positive childhood experiences on adult health outcomes in the context of adverse childhood experiences: a nationwide cross-sectional survey in Japan

Authors

Haruyo Mitani[1], Naoki Kondo[2], Airi Amemiya, Takahiro Tabuchi[3]

Author Affiliations

[1]Graduate School of Human Sciences, Osaka University, Osaka, Japan

[2]Department of Social Epidemiology, Graduate School of Medicine and School of Public Health, Kyoto University, Kyoto, Japan

[3]Cancer Control Center, Osaka International Cancer Institute, Osaka, Japan

Correspondence to

Haruyo Mitani, Graduate School of Human Sciences, Osaka University, Osaka, Japan

haruyo.mitani@gmail.com

For peer review only - http://bmjopen.bmj.com/site/about/guidelines.xhtml

ABSTRACT

Objective: Although adverse childhood experiences (ACEs) are associated with poor health in adulthood, positive childhood experiences (PCEs) can reduce the risk of negative health outcomes. This study aimed to investigate whether PCEs in the community (CPCEs, i.e., trusted adults other than parents, supportive friends, belongingness to school, or community traditions) would have an independent effect on better health outcomes and moderate the association between ACEs and adult illnesses.

Design: Cross-sectional survey

Setting: Data were gathered from a nationwide, cross-sectional Internet survey conducted in Japan in 2022.

Participants: This study included 28,254 Japanese adults aged 18–82 years (51.1% female; mean age = 47.6 years).

Primary and secondary outcome measures: The associations among self-reported ACEs, CPCEs before the age of 18 years, and current chronic diseases (e.g., cancer and depression) were investigated using multivariable logistic regression models.

Results: CPCEs were associated with lower odds of adult diseases (such as stroke, chronic obstructive pulmonary disease [COPD], chronic pain, depression, suicidal ideation, and severe psychological distress) after adjusting for ACEs. More CPCEs weakened the association between ACEs and adult diseases. Specifically, among those with ACEs, ≥3 CPCEs (vs. 0–2 CPCEs) reduced the adjusted prevalence by ≥50% for stroke (2.4→1.2%), COPD (2.2→0.7%), and severe psychological distress (16.4→7.4%).

Conclusion: CPCEs could reduce ACE-related risk of poor physical and mental health in later

For peer review only - http://bmjopen.bmj.com/site/about/guidelines.xhtml

life. Early-life interventions that enhance PCEs in schools and/or neighborhoods are recommended.

Strengths and limitations of this study

- This study used a large and representative sample from a nationwide survey in Japan.
- Multivariable logistic regression models were used to adjust for possible confounders and to evaluate the associations among ACEs, CPCEs, and adult diseases.
- Due to the self-reported and retrospective nature of the survey data, the possibility of recall bias cannot be eliminated.
- A cross-sectional design limited our ability to determine causality.
- The CPCEs measure used in this study did not include all CPCEs.

For peer review only

For peer review only - http://bmjopen.bmj.com/site/about/guidelines.xhtml

INTRODUCTION

Over the last quarter-century, a growing body of research has demonstrated the harmful effects of adverse childhood experiences (ACEs), such as maltreatment and exposure to household dysfunction, on long-term physical and mental health[1–6]. Studies have emphasized a dose-response relationship between an individual's ACE score and their risk of having poor health; that is the greater the exposure to ACEs, the greater the risk of physical and mental illness in later life. Trauma-related extreme stress impairs the stress response and self-regulation capacity, disrupting typical brain development[7,8].

Meanwhile, recent evidence shows that positive childhood experiences (PCEs) have a moderating effect as a protective factor; that is, the harmful effects of ACEs can be smaller among PCEs-rich individuals than among PCEs-poor individuals[9–15]. For example, continual early assistance from a reliable adult[9], an adult who felt safe and protected as a child[12], or PCEs inside and outside the home (cumulative scale consisting of seven items such as "able to talk to family about feelings" and "felt supported by friends")[10], moderates the association between ACEs and negative consequences, represented by poor mental health.

Little is understood about the potential of PCEs in the community (CPCEs) for countering the negative lifetime effects of ACEs. Resilience refers to the latent or apparent capacity to successfully respond to situations that threaten an individual's functioning, survival, or development[16]. Based on the Resilience Theory[16], the socio-ecological system suggests that multiple systems (e.g., individuals, families, and communities) complementarily influence youth development. Moreover, factors promoting resilient outcomes can be divided into individual (e.g., cognition, coping style, and self-esteem), family (e.g., a safe and supportive relationship with a caregiver), and community factors (e.g., positive relationships with friends and neighbors)[16–19]. Notably, the concept of PCEs includes both family and community

For peer review only - http://bmjopen.bmj.com/site/about/guidelines.xhtml

factors.

The present study focused on the role of CPCEs (PCEs in the community outside the home). Children exposed to ACEs were more likely to have been unable to talk to their parents about their feelings or feel safe. In other words, they are less likely to have experienced PCEs in the home. Hence, continuous family intervention by professionals to change how caregivers relate to children is desirable to enrich family PCEs (FPCEs)[20]. However, many countries lack the infrastructure (staff, funds, and legal structure) to make such professional intervention possible, and Japan is no exception[21]. However, community interventions in schools and neighborhoods can easily and inexpensively positively impact the development of children exposed to ACEs. Clarifying the role of CPCEs is essential for understanding the effectiveness of community interventions.

Therefore, this study explored the effect of CPCEs on the negative consequences of ACEs, focusing on four items of the PCEs scale[10], referenced in many studies, that are considered particularly present in the community (i.e., trusted adults other than parents, supportive friends, belongingness to school, and community traditions). Similar to the original ACE study[1], this study used a broad range of physical and mental illnesses as primary outcomes. In Japan, although ACEs affect physical illnesses in old age[22] and mental disorders in adults[23], the interaction between PCEs and ACEs on health has not been examined. Stress is one of the causes associated with adult diseases[24]. ACEs would be positioned as stressors within the childhood home and CPCEs as factors outside the home that soften the impact of those stressors.

This study explored the associations among ACEs, CPCEs, and adult diseases. First, the relationship between ACEs and adult diseases was ascertained. Then, the Resilience Theory's three main models (the Compensatory Model, the Protective Factors Model, and the Challenge Model [16,25–27]) were examined.

For peer review only - http://bmjopen.bmj.com/site/about/guidelines.xhtml

The Compensatory (Promotive Factors) Model assumes that promotive/compensatory factors directly impact developmental outcomes independent of risk factors. While ACEs could still have a significant effect on the outcome, CPCEs themselves are thought to enhance health in adulthood.

The Protective Factors Model assumes that protective factors mitigate the relationship between risk factors and bad outcomes. Protective factors do not act independently but can interact with risk factors. In other words, especially in situations where CPCEs are abundant, the negative impact of ACEs on adult health is likely to be smaller.

The Challenge Model assumes that the modest levels of risk factors protect against additional exposures that could render people prone to unfavorable consequences. Moderate levels of ACEs, likely especially in situations with abundant protective factors, may promote better adult health, as if inoculation[11]. It is possible to speculate about whether ACEs can lower the likelihood of adult illness among rich-CPCEs individuals.

Thus, this study tested three hypotheses: CPCEs directly affect adult health (H1), the association between ACEs and adult diseases is weaker among people with more CPCEs (H2) and ACEs are negatively associated with adult disease among people with more CPCEs (H3).

METHODS

Sample

The Japan COVID-19 and Society Internet Survey (JACSIS)[28] was conducted in 2022 by a major Internet research agency (Rakuten Insight, Inc.) with around 2.2 million registered qualified panelists. Of the panelists, 224,389 males and females aged 15–79 were invited to

For peer review only - http://bmjopen.bmj.com/site/about/guidelines.xhtml

participate in the survey based on a random sample stratified by sex, age, and all 47 prefectures. Participants were rewarded with "E-Points" for online shopping. Informed consent was obtained from all participants before the survey. The survey began on September 12, 2022 and ended on October 19, 2022. The survey was terminated after the target of 32,000 participants was reached.

This study was authorized by the Research Ethics Committee of the Osaka International Cancer Institute (approved on June 14, 2022; approval No. 20084-8) and followed the Helsinki guidelines.

Participants who provided contradictory answers to the screening questions were excluded from the study. These included the following criteria: (i) invalid answers to the screening question (participants were asked to choose the second-last option, but they chose other options), (ii) positive responses to all items on drug use, (iii) positive answers for having all 20 chronic diseases, and (iv) a total household size of over 15 individuals. There were 3,370 respondents who gave inconsistent answers, leaving 28,630 individuals. Those aged <18 were also excluded since ACEs and PCEs are answered by recalling experiences up to age 18. Finally, 28,617 adults were included. Due to the nature of the online survey, there were no missing values.

Patient and public involvement

Neither patients nor the public were involved in this study.

For peer review only - http://bmjopen.bmj.com/site/about/guidelines.xhtml

Measurements

ACEs

Questions on ACEs were derived from the ACEs scale for the Japanese context[29]. Although this study was based on the original ACE study[1], ACE items included parental death, except the incarceration of household members, to fit the Japanese context. Respondents were questioned whether any of the following adversities had occurred in their households by age 18: (i) death of either parent; (ii) parental divorce or separation; (iii) mental illness of either parent; (iv) substance abuse in the household (e.g., parental addiction to alcohol and gambling); (v) mother treated violently (the father was violent with the mother); (vi) emotional abuse (my parent said hurtful things or insulted me); (vii) physical abuse (my parent beat and hurt me); (viii) sexual abuse (an adult sexually touched me); (ix) emotional neglect (I always felt suffocated because my parent did not respect my opinion); and (x) physical neglect (I did not receive necessary care, such as eating and dressing). The response categories “yes” and “no” were coded as dummy variables, and these 10 items were summed into ACE scores (0–10). This score was classified into two categories (0 vs. ≥1) because of their effectiveness in determining poor health, following those of previous studies[22,30] and the clarity of the semantic difference between "without ACEs" and "with ACEs."

PCEs

PCEs were assessed using seven items adapted from the PCEs scale[10]. Participants answered whether they had experienced any of the following by the time they were 18 years old: (i) felt able to talk to their family about feelings; (ii) felt their family stood by them during difficult times; (iii) felt safe and protected by an adult in their home; (iv) had at least two non-parental

For peer review only - http://bmjopen.bmj.com/site/about/guidelines.xhtml

adults who took genuine interest in them; (v) felt supported by friends; (vi) felt a sense of belonging in middle and high school; and (vii) enjoyed participating in community traditions. Although these questions were asked in Japanese, and some were modified from the original questions (the addition of “middle” in item 6), these seven items can be considered almost identical to the PCEs items used by Bethell et al.[10] Of these items, the first three were positioned as components of FPCEs and the others as CPCEs (Supplementary Table S1). The applicable items were summed to obtain the FPCE (0–3) and CPCE (0–4) scores. Like the PCEs scale[10], principal component analyses revealed a single-component structure for each FPCE and CPCE scale. Cronbach's alpha was 0.81 and 0.75 for FPCEs and CPCEs, respectively (Supplementary Table S2).

Adult health outcomes

Participants answered whether they currently had the following diseases using a dichotomous response variable (yes/no): (i) diabetes, (ii) ischemic heart disease (angina pectoris or myocardial infarction), (iii) stroke (cerebral infarction or hemorrhage), (iv) chronic obstructive pulmonary disease (COPD), (v) cancer, (vi) chronic pain (e.g., back pain and headache lasting ≥3 months), and (vii) depression. Additionally, (viii) suicidal ideation was determined as a “yes” response to the question, “Have you had times when you have wanted to die in the last year?” Psychological distress was measured with the Japanese version[31] of the Kessler 6-item psychological distress scale (K6)[32], assessing depressed mood and anxiety. The total score ranged from 0 to 24 on a five-point scale (0–4). A score of ≥13 was considered indicative of (ix) serious psychological distress[33].

For peer review only - http://bmjopen.bmj.com/site/about/guidelines.xhtml

Covariates

The following background variables reported by participants were included as covariates, in line with previous studies[1,13,34]: age (continuous by year), sex (0 “male” or 1 “female”), and educational attainment (three dummy variables for “less than high school,” “high school,” and “post-secondary and graduate”).

Statistical analysis

Demographic characteristics and prevalence rates of all health outcomes in the sample were examined. For H1, multivariable logistic regression analyses were conducted to consider the direct effect of CPCEs on adult diseases after adjusting for covariates, ACEs, and FPCEs. For H2 and H3, multiple logistic regression analyses, stratified by CPCE exposure levels, were performed to examine whether CPCEs moderated the link between ACEs and adult diseases, adjusting for covariates and FPCEs. Interactive effects and marginal predicted probabilities were estimated to assess the statistical significance of the moderating effects of CPCEs on the association between ACEs and adult diseases[35]. The extent to which CPCEs reduced the risk of each disease was also estimated.

The survey data were weighted to represent the Japanese population. Considering the possibility of multicollinearity, the variance inflation factor (VIF) for each explanatory variable was checked to ensure that it did not exceed 4.0[36]. Stata 17.0 MP (StataCorp LLC, Texas, USA) was used for all analyses.

For peer review only - http://bmjopen.bmj.com/site/about/guidelines.xhtml

RESULTS

Participant characteristics

Table 1 shows that chronic pain and COPD were the most prevalent (18.6%) and least prevalent (1.4%) of the nine health outcomes, respectively. Approximately 39.8% of the participants had experienced ACEs. Furthermore, 52.8% and 56.0% of participants had experienced one or more FPCEs and CPCEs, respectively. When ACEs were divided into four exposure levels[9,10], the more exposure to ACEs, the higher the prevalence of adult diseases, the less exposure rates to the two types of PCEs, and the higher the proportion of females and those with lower education levels (Supplementary Table S3). Finally, dose-response relationships between ACE exposure levels and all adult diseases were observed (Supplementary Table S4).

Table 1 Demographic characteristics and prevalence of health outcomes, ACEs, and PCEs among participants (N = 28617)

	N	W %/M (SD)
Health outcomes		
Diabetes	1945	7.7
Ischemic heart disease	686	3.0
Stroke	433	1.7
COPD	381	1.4
Cancer	705	2.6
Chronic pain	5062	18.6
Depression	1381	4.7
Suicidal ideation	3909	14.4
Severe psychological distress	2881	10.4
ACEs (0–11)	28617	0.81 (1.4)
0 ACEs	17889	60.2
≥1 ACEs	10728	39.8
FPCEs (0–3)	28617	1.14 (1.2)
0 FPCE	12003	47.2
1 FPCEs	4133	14.0

For peer review only - http://bmjopen.bmj.com/site/about/guidelines.xhtml

2 FPCEs	5025	16.2
3 FPCEs	7456	22.7
CPCEs (0–4)	28617	1.25 (1.4)
0 CPCE	11051	44.0
1 CPCEs	5543	19.4
2 CPCEs	4502	14.7
3 CPCEs	3922	11.5
4 CPCEs	3599	10.5
Age (18–82 years)	28617	48.1 (17.2)
Sex		
Male	13993	48.9
Female	14624	51.1
Educational attainment		
Less than HS	493	4.4
HS graduate	10516	60.3
Post-secondary and graduate	17397	33.8

W, weighted; SD, standard deviation; ACEs, adverse childhood experiences; CPCEs, community positive childhood experiences; FPCEs, family positive childhood experiences; HS, high school

For peer review only

Health outcomes according to ACE, FPCE, and CPCE exposure

Table 2 shows the adjusted odds ratios (AORs) for the poor health associated with CPCEs. In all models, VIF values did not exceed the criterion value (4.0). After adjusting for covariates, ACEs, and FPCEs, CPCEs had significant negative direct effects on six adult diseases: stroke, COPD, chronic pain, depression, suicidal ideation, and severe psychological distress, supporting H1. For example, each additional CPCE decreased the adjusted odds of stroke by 15%. The same trend was found when CPCEs were treated as categorical variables (Supplementary Table S5). Overall, CPCEs were particularly associated with lower odds of mental illness.

For peer review only - http://bmjopen.bmj.com/site/about/guidelines.xhtml

Table 2 Adjusted odds ratios of health outcomes by ACEs (≥1 vs. 0), FPCEs, and CPCEs

	ACEs		FPCEs		CPCEs	
	**AOR***	**(95% CI)**	**AOR†**	**(95% CI)**	**AOR‡**	**(95% CI)**
Diabetes	**1.31**	(1.18–1.44)	**0.93**	(0.89–0.99)	0.97	(0.93–1.02)
Ischemic heart disease	**1.53**	(1.31–1.80)	**0.89**	(0.82–0.97)	0.93	(0.87–1.01)
Stroke	**1.96**	(1.61–2.39)	**0.83**	(0.75–0.93)	**0.85**	(0.77–0.93)
COPD	**2.00**	(1.62–2.47)	**0.77**	(0.69–0.86)	**0.89**	(0.81–0.98)
Cancer	**1.62**	(1.39–1.90)	**0.89**	(0.82–0.97)	0.95	(0.89–1.02)
Chronic pain	**1.71**	(1.60–1.82)	0.98	(0.95–1.02)	**0.91**	(0.89–0.94)
Depression	**2.86**	(2.55–3.22)	**0.89**	(0.83–0.95)	**0.81**	(0.76–0.86)
Suicidal ideation	**2.12**	(1.97–2.28)	**0.84**	(0.81–0.88)	**0.77**	(0.74–0.80)
Severe psychological distress	**2.29**	(2.10–2.48)	**0.86**	(0.81–0.90)	**0.72**	(0.69–0.75)

N=28617; Significance level of data in bold font <0.05.

*Adjusted for age, sex, educational attainment, FPCEs, and CPCEs.

†Adjusted for age, sex, educational attainment, ACEs, and CPCEs.

‡Adjusted for age, sex, educational attainment, ACEs, and FPCEs.

ACEs, adverse childhood experiences; FPCEs, family positive childhood experiences; CPCEs, community positive childhood experiences; AOR, adjusted odds ratio; CI, confidence interval.

Health outcomes of ACEs across CPCE exposure levels

As shown in Figure 1, the AORs declined as PCE exposure levels rose; the cells become greener to the right. Particularly, the AORs tended to be smaller when the CPCE scores were ≥3 than when they were 0–2. This value of 3 CPCEs is also consistent with the Aiken and West[37] method, which recommends that the cut-off value is close to the mean plus one standard deviation (mean1.25+sd1.4 = 2.65). Therefore, the interaction effects of ACEs and CPCEs (≥3 vs. 0–2) on all nine outcomes were examined (Supplementary Table S6). Although the rightmost column of Figure 1 shows that the regression coefficients on the interaction terms between any ACE and ≥3 CPCEs were significant at the 10% level for seven diseases, it is a

For peer review only

For peer review only - http://bmjopen.bmj.com/site/about/guidelines.xhtml

better way to examine the predicted probability themselves and perform the test of the equality of marginal effects to determine whether significant interaction effects exist[35].

Figure 2 shows the predicted values for each disease obtained from the regression equations. The results of the marginal effect difference test (the red values of Figure 2 and Supplementary Table S7) showed significant differences in the marginal effects (the amount of change in predicted probability when ACEs exceed ≥1 from 0) between those with 0–2 CPCEs and those with ≥3 CPCEs for eight diseases except diabetes. In support of H2, CPCEs moderated the association between ACEs and eight adult diseases. Specifically, in ischemic heart disease, the difference between the marginal effect of 0–2 CPCEs (0.034−0.21 = 0.013) and one of ≥3 CPCEs (0.017−0.019 = −0.001) was 0.015, significant at the 1% level. According to each marginal effect (Supplementary Table S7), the marginal effect for CPCEs ≥ 3 was negative for ischemic heart disease (−0.001, 95% CI −0.008 to 0.006) and COPD (−0. 002, 95% CI −0.006 to 0.003). A negative association between ACEs and adult disease in the context of an abundance of CPCEs was suggested, but H3 was not statistically supported.

From Figure 2, the differences in predicted morbidity by CPCEs among those with ACEs can also be captured as relative risk reduction. For example, the predicted prevalence of ischemic heart disease did not differ according to CPCE exposure among those without ACEs; however, among those with ACEs, it differed from 3.4% in those with 0–2 CPCEs to 1.7% in those with ≥3 CPCEs (relative risk: 1.725/3.400*100 = 50.794%, relative risk reduction: 100.0 −50.8 = 49.2%). The adjusted prevalence was reduced by almost ≥50% for ischemic heart disease (49.2%), stroke (51.9%), COPD (67.1%), depression (49.1%), and severe psychological distress (54.9%).

For peer review only - http://bmjopen.bmj.com/site/about/guidelines.xhtml

DISCUSSION

This study examined the associations among ACEs, CPCEs, and adult diseases using a population-based sample from Japan. Specifically, ACEs were associated with increased odds of adult diseases, while CPCEs directly affected adult illnesses as a promotive factor and mitigated the adverse health effects of ACEs as a protective factor. The protective effect of CPCEs was particularly pronounced in stroke, COPD, and severe psychological distress, with relative risk reductions of 50% or more by three or more CPCEs in those with ACEs.

Consistent with earlier research[1,4,6], the results showed a dose-response connection between ACEs and all adult diseases evaluated. Although an association between ACEs and diseases during old age among Japanese individuals was previously reported[22], this appeared robust across a wide age range (18–82 years). The ACE items are based on the original ACE study[1]. Although this measure does not include the variety of traumatic experiences that participants may have, it is noteworthy that a dose-response relationship with adult health was found, as in many studies.

H1, based on the Compensatory Model, was supported for many of the diseases assessed in this study. CPCEs, independent of ACEs and FPCEs, were directly associated with lower odds of adult-onset illnesses (i.e., stroke, COPD, chronic pain, depression, suicidal ideation, and severe psychological distress). While previous studies have found direct effects of PCEs primarily on mental health[10,11,13], the present study adds a novel finding that CPCEs independently function as promotive factors for physical and mental health.

More importantly, this study found analytical support for H2 based on the Protective Model. CPCEs moderated the relationships between ACEs and adult diseases (i.e., ischemic heart disease, stroke, COPD, cancer, chronic pain, depression, suicidal ideation, and severe

For peer review only - http://bmjopen.bmj.com/site/about/guidelines.xhtml

psychological distress), even after adjusting for FPCEs. Although the possible protective function of PCEs has been noted[9,10,12], the buffering effect may be due to family factors. Notably, the present study observed that CPCEs can independently play a protective role. Furthermore, extending research focusing on community assets moderating the association between ACEs and poor child health[15], this study suggests that the moderating effects of CPCEs may spread to adulthood, indicating that the protective effect may be stronger in cumulative situations where three or more CPCEs are aligned. The relative risk reduction of physical illness (i.e., stroke and COPD) and mental illness (i.e., depression and severe psychological distress) in ACE survivors by almost more than half due to ≥3 CPCEs would be a non-negligible value. Whether this cutoff point is appropriate requires follow-up testing; however, the harm caused by ACEs may be mitigated in multiple relationships.

Moreover, the analysis yielded the finding that, for several diseases (i.e., ischemic heart disease and COPD), those with ACEs had lower prevalence rates than those without ACEs in situations of CPCE abundance (Figure 2). Although the negative marginal effects of ACEs on these illnesses were not statistically significant, as the Challenge Model[26] of the Resilience Theory implies, moderate ACEs promote health when sufficient protective factors exist; that is, they act as immunization. Future studies should examine the generalizability of this phenomenon.

Why might CPCEs contribute to developing children and youth into healthier adults? Prior research argues that supportive adults other than parents may provide opportunities for children to elude life stressors, establish positive experiences in social connections, and feel a sense of having a place and social association[38]. Additionally, supportive close friendships promote resilience by developing constructive coping styles, encouraging efforts, providing supportive peer networks, and reducing inappropriate coping[39]. In interactions with non-family

For peer review only - http://bmjopen.bmj.com/site/about/guidelines.xhtml

members at school and in the neighborhood, children exposed to ACEs may gain physical refuge from toxic stress and develop psychological and instrumental coping strategies. The role of CPCEs as a promoting and protective factor found in this study may have been discovered only in the context of Japan, where many local communities have self-governing organizations and traditional events, and violence among residents and students is relatively rare. Future studies should examine whether the findings are generalizable to other countries besides Japan.

Strengths and limitations

To the best of our knowledge, this is the first study to prove that CPCEs could be promotive and protective factors of health system resilience. A strength of this study is the use of a large and representative sample. Moreover, various indicators elucidated the complex relationships among ACEs, PCEs, and adult diseases.

However, this study had some limitations. First, the data were self-reported and retrospective, which could have been influenced by recall bias. Individuals with a disease may be more likely to recall ACEs than healthy individuals. However, asking participants to reflect on their growing environment from a balanced perspective by asking about PCEs may have reduced this recall bias. Second, because of the study's cross-sectional nature, the possibility of reverse causation between ACEs/PCEs and adult illnesses cannot be eliminated. This study could not allow for the determination of causality. Third, the CPCEs measure used in this study did not include all CPCEs. Other types of CPCEs, such as participation in non-traditional activities and self-realization experiences, should be included in developing the CPCEs scale. Fourth, confounding variables (including psychological traits like optimism and hometown regional features, which may be linked to adult diseases, ACEs, and CPCEs) might have gone unnoticed.

For peer review only

For peer review only - http://bmjopen.bmj.com/site/about/guidelines.xhtml

Fifth, the extent of the positive effects of the CPCEs differed for each health outcome. Particularly, the role of CPCEs in mental illness was found to be robust; however, the validity of these effects should be carefully investigated by including other health outcomes. Sixth, this study could not address a variety of traumatic experiences (e.g., homelessness, bullying victimization, childhood illness, etc.) other than typical ACEs.

Policy, practice, and research implications

The findings suggest that community-based interventions can be effective in helping children who have experienced adversity become resilient adults. Public health, social welfare, and educational practices that enforce CPCEs might as well be promoted. Additionally, the content of specific community-level approaches depends on social and local contexts. Such interventions are desirable to incorporate the main components of the Healthy Outcomes from Positive Experiences framework: (i) supportive and nurturing relationships, (ii) safe and stable environments, (iii) opportunities for constructive social involvement and connection, and (iv) social and emotional capacities[40]. For example, The National Resource Center at Tufts Medical Center[41] supports the above framework, providing tools and training to help healthcare providers, social service providers, educators, and members of community-based organizations acquire essential knowledge and skills to integrate these elements into their work.

In Japan, school counselors[42] and school social workers[43] in public elementary and junior high schools are expected to become resilience resources under compulsory education. Additionally, teachers might be a good way to get training in PCEs and improved working environments to build trusting relationships with their students. In local communities, traditional events such as festivals, nature experiences, multigenerational exchange programs,

For peer review only

For peer review only - http://bmjopen.bmj.com/site/about/guidelines.xhtml

and the recently popularized "*Kodomo Shokudo*" (cafeterias for children)[44] could also contribute to the children's resilience. Further research and practice are expected to explore effective approaches to promoting CPCEs, including historical and new engagements.

Conclusion

This study showed that CPCEs positively impacted adult health in the general population regardless of ACE exposure and mitigated the adverse consequences. The study demonstrates that CPCEs may contribute to the lifelong health status of ACE survivors. Therefore, further research is needed to investigate the impact mechanisms of CPCEs and improve the scale's accuracy while capturing diverse childhood adversities, as well as interventions to strengthen supportive communities.

Contributors: HM and AA conceptualized the study. HM conducted the analysis and drafted the first manuscript. NK and AA contributed intellectual content to the study design and interpretation of the findings. TT collected data, managed the project, and obtained funding. All authors critically reviewed the manuscript and approved the final manuscript. HM is the guarantor for this study.

Funding: This study (JACSIS2022) was supported by the Japan Society for the Promotion of Science (JSPS) KAKENHI Grants (21H04856, 20K10467, 20K19633, 20K13721), the JST Grant (JPMJPF2017), the Health Labor Sciences Research Grant (21HA2016), the grant for 2021-2022 Strategic Research Promotion (SK202116) of Yokohama City University, and the

For peer review only - http://bmjopen.bmj.com/site/about/guidelines.xhtml

research program on "Using Health Metrics to Monitor and Evaluate the Impact of Health Policies," conducted at the Tokyo Foundation for Policy Research. HM was also supported by the JSPS KAKENHI Grant (21K13454) and the Ministry of Education, Culture, Sports, Science and Technology (MEXT) KAKENHI Grant (20H05805).

Competing interests: None declared.

Data availability statement: All de-identified data from participants reported in this study are available to interested researchers who submit data-sharing requests to the last author with a summary of the secondary analysis plan.

ORCID iDs:

Haruyo Mitani https://orcid.org/0000-0001-8882-5098

Naoki Kondo https://orcid.org/0000-0002-6425-6844

Airi Amemiya https://orcid.org/0000-0001-5319-9541

Takahiro Tabuchi http://orcid.org/0000-0002-1050-3125

REFERENCES

1 Felitti VJ, Anda RF, Nordenberg D, *et al.* Relationship of childhood abuse and household dysfunction to many of the leading causes of death in adults. The Adverse Childhood Experiences (ACE) Study. *Am J Prev Med* 1998;**14**:245–58. doi:10.1016/s0749-3797(98)00017-8

2 Anda RF, Felitti VJ, Bremner JD, *et al.* The enduring effects of abuse and related adverse experiences in childhood: a convergence of evidence from neurobiology and epidemiology. *Eur Arch Psychiatry Clin Neurosci* 2006;**256**:174–86. doi:10.1007/s00406-005-0624-4

3 Bellis MA, Hughes K, Ford K, *et al.* Life course health consequences and associated annual costs of adverse childhood experiences across Europe and North America: a systematic review and meta-analysis. *Lancet Public Heal* 2019;**4**:e517–28. doi:10.1016/S2468-2667(19)30145-8

For peer review only - http://bmjopen.bmj.com/site/about/guidelines.xhtml

4 Hughes K, Bellis MA, Hardcastle, A K, *et al.* The effect of multiple adverse childhood experiences on health: a systematic review and meta-analysis. *Lancet Public Heal* 2017;**2**:e356–66.https://doi.org/10.1016/S2468-2667(17)30118-4.

5 Norman RE, Byambaa M, De R, *et al.* The long-term health consequences of child physical abuse, emotional abuse, and neglect: a systematic review and meta-analysis. *Plos Med* 2012;**9**:1–31. doi:10.1371/journal.pmed.1001349

6 Petruccelli K, Davis J, Berman T. Adverse childhood experiences and associated health outcomes: a systematic review and meta-analysis. *Child Abus Negl* 2019;**97**:104127. doi:10.1016/j.chiabu.2019.104127

7 Shonkoff JP, Garner AS, Siegel BS, *et al.* The lifelong effects of early childhood adversity and toxic stress. *Pediatrics* 2012;**129**:e232–46. doi:10.1542/PEDS.2011-2663

8 Oral R, Ramirez M, Coohey C, *et al.* Adverse childhood experiences and trauma informed care: the future of health care. *Pediatr Res* 2016;**79**:227–33. doi:10.1038/pr.2015.197

9 Bellis MA, Hardcastle K, Ford K, *et al.* Does continuous trusted adult support in childhood impart life-course resilience against adverse childhood experiences - a retrospective study on adult health-harming behaviours and mental well-being. *BMC Psychiatry* 2017;**17**:110. doi:10.1186/S12888-017-1260-Z/TABLES/3

10 Bethell C, Jones J, Gombojav N, *et al.* Positive childhood experiences and adult mental and relational health in a statewide sample: associations across adverse childhood experiences levels. *JAMA Pediatr* 2019;**173**:e193007. doi:10.1001/jamapediatrics.2019.3007

11 Crandall AA, Miller JR, Cheung A, *et al.* ACEs and counter-ACEs: how positive and negative childhood experiences influence adult health. *Child Abuse Negl* 2019;**96**:104089. doi:10.1016/J.CHIABU.2019.104089

12 Crouch E, Radcliff E, Strompolis M, *et al.* Safe, stable, and nurtured: protective factors against poor physical and mental health outcomes following exposure to adverse childhood experiences (ACEs). *J Child Adolesc Trauma* 2019;**12**:165–73. doi:10.1007/S40653-018-0217-9/METRICS

13 Kuhar M, Zager Kocjan G. Associations of adverse and positive childhood experiences with adult physical and mental health and risk behaviours in Slovenia. *Eur J Psychotraumatol* 2021;**12**:1924953. doi:10.1080/20008198.2021.1924953

For peer review only - http://bmjopen.bmj.com/site/about/guidelines.xhtml

14 Narayan AJ, Rivera LM, Bernstein RE, *et al.* Positive childhood experiences predict less psychopathology and stress in pregnant women with childhood adversity: a pilot study of the benevolent childhood experiences (BCEs) scale. *Child Abuse Negl* 2018;**78**:19–30. doi:10.1016/J.CHIABU.2017.09.022

15 Bellis MA, Hughes K, Ford K, *et al.* Adverse childhood experiences and sources of childhood resilience: a retrospective study of their combined relationships with child health and educational attendance. *BMC Public Health* 2018;**18**:1–12. doi:10.1186/S12889-018-5699-8/FIGURES/1

16 Masten AS, Cicchetti D. Resilience in development: progress and transformation. In: D. Cicchetti, ed. *Developmental Psychopathology, Risk, Resilience, and Intervention*. John Wiley & Sons, Inc. 2016. 271–333. doi:https://doi.org/10.1002/9781119125556.devpsy406

17 Gartland D, Riggs E, Muyeen S, *et al.* What factors are associated with resilient outcomes in children exposed to social adversity? a systematic review. *BMJ Open* 2019;**9**:e024870. doi:10.1136/BMJOPEN-2018-024870

18 Henson M, Sabo S, Trujillo A, *et al.* Identifying protective factors to promote health in american indian and alaska native adolescents: a literature review. *J Prim Prev* 2017;**38**:5–26. doi:10.1007/S10935-016-0455-2/METRICS

19 Okwori G. Role of individual, family, and community resilience in moderating effects of adverse childhood experiences on mental health among children. *J Dev Behav Pediatr* 2022;**43**:E452–62. doi:10.1097/DBP.0000000000001076

20 Molloy C, Beatson R, Harrop C, *et al.* Systematic review: effects of sustained nurse home visiting programs for disadvantaged mothers and children. *J Adv Nurs* 2021;**77**:147–61. doi:10.1111/JAN.14576

21 Isumi A, Doi S, Ochi M, *et al.* School- and community-level protective factors for resilience among chronically maltreated children in Japan. *Soc Psychiatry Psychiatr Epidemiol* 2023;**58**:477–88. doi:10.1007/S00127-022-02322-X/TABLES/5

22 Amemiya A, Fujiwara T, Shirai K, *et al.* Association between adverse childhood experiences and adult diseases in older adults: a comparative cross-sectional study in Japan and Finland. *BMJ Open* 2019;**9**:e024609. doi:10.1136/bmjopen-2018-024609

23 Fujiwara T, Kawakami N. Association of childhood adversities with the first onset of mental disorders in Japan: results from the World Mental Health Japan, 2002-2004. *J*

For peer review only - http://bmjopen.bmj.com/site/about/guidelines.xhtml

Psychiatr Res 2011;**45**:481–7. doi:10.1016/j.jpsychires.2010.08.002

24 Cohen S, Gianaros PJ, Manuck SB. A stage model of stress and disease. *Perspect Psychol Sci* 2016;**11**:456–63. doi:10.1177/1745691616646305

25 Mueller KC, Carey MT. How positive and negative childhood experiences interact with resiliency theory and the general theory of crime in juvenile probationers. *Youth Violence Juv Justice* 2022;**21**:130–48. doi:10.1177/15412040221131278

26 Zimmerman MA. Resiliency theory: a strengths-based approach to research and practice for adolescent health. *Heal Educ Behav* 2013;**40**:381–3. doi:10.1177/1090198113493782

27 Oshri A, Duprey EK, Liu S, *et al.* ACEs and resilience: methodological and conceptual issues. In: Asmundson GJG, Afifi TO, eds. *Adverse Childhood Experiences: Using Evidence to Advance Research, Practice, Policy, and Prevention*. Academic Press 2020. 287–306.

28 JACSIS study. The Japan COVID-19 and Society Internet Survey. 2021.https://jacsis-study.jp/

29 Fujiwara T. Impact of adverse childhood experience on physical and mental health: a life-course epidemiology perspective. *Psychiatry Clin Neurosci* 2022;**76**:544–51. doi:10.1111/PCN.13464

30 Cheong E Von, Sinnott C, Dahly D, *et al.* Adverse childhood experiences (ACEs) and later-life depression: perceived social support as a potential protective factor. *BMJ Open* 2017;**7**:e013228. doi:10.1136/BMJOPEN-2016-013228

31 Furukawa TA, Kawakami N, Saitoh M, *et al.* The performance of the Japanese version of the K6 and K10 in the World Mental Health Survey Japan. *Int J Methods Psychiatr Res* 2008;**17**:152–8. doi:10.1002/mpr.257

32 Kessler RC, Andrews G, Colpe LJ, *et al.* Short screening scales to monitor population prevalences and trends in non-specific psychological distress. *Psychol Med* 2002;**32**:959–76. doi:10.1017/S0033291702006074

33 Kessler RC, Green JG, Gruber MJ, *et al.* Screening for serious mental illness in the general population with the K6 screening scale: results from the WHO World Mental Health (WMH) survey initiative. *Int J Methods Psychiatr Res* 2010;**19**:4–22. doi:10.1002/mpr.310

For peer review only

For peer review only - http://bmjopen.bmj.com/site/about/guidelines.xhtml

34 Metzler M, Merrick MT, Klevens J, *et al.* Adverse childhood experiences and life opportunities: shifting the narrative. *Child Youth Serv Rev* 2017;**72**:141–9. doi:10.1016/j.childyouth.2016.10.021

35 Mize TD. Best practices for estimating, interpreting, and presenting nonlinear interaction effects. *Sociol Sci* 2019;**6**:81–117. doi:10.15195/V6.A4

36 Hair JF, Black B, Babin BJ, *et al. Multivariate Data Analysis: Global Edition*. 7th ed. Pearson Education 2009.

37 Aiken LS, West SG. *Multiple Regression: Testing and Interpreting Interactions*. Newbury Park, CA: Sage Publications 1991.

38 Rhodes JE, Spencer R, Keller TE, *et al.* A model for the influence of mentoring relationships on youth development. *J Community Psychol* 2006;**34**:691–707. doi:10.1002/JCOP.20124

39 Graber R, Turner R, Madill A. Best friends and better coping: facilitating psychological resilience through boys' and girls' closest friendships. *Br J Psychol* 2016;**107**:338–58. doi:10.1111/BJOP.12135

40 Sege RD, Harper Browne C. Responding to ACEs with HOPE: Health Outcomes from Positive Experiences. *Acad Pediatr* 2017;**17**:S79–85. doi:10.1016/J.ACAP.2017.03.007

41 The National Resource Center at Tufts Medical Center. Tufts HOPE: Healthy Outcomes from Positive Experiences. 2023.https://positiveexperience.org/

42 Nishio A, Kakimoto M, Horita R, *et al.* Compulsory educational mental health support system in Japan. *Pediatr Int* 2020;**62**:529–34. doi:10.1111/PED.14205

43 Kanazawa M. Current issues of school social work in Japan : from the historical relations between school, social work, and counseling. *Japanese J Soc Serv* 2009;**5**:159–71. doi:10.24469/JJSWE.5.0_159

44 Murayama H, Kurotani K, Tabuchi T. Social capital and the spread of children's cafeterias (kodomo shokudo) in Japan: an ecological analysis. *Asia-Pacific J public Heal* 2022;**34**:817–20. doi:10.1177/10105395221119416

For peer review only - http://bmjopen.bmj.com/site/about/guidelines.xhtml

Figure legends

Figure 1

A heatmap of the adjusted odds ratios with 95% CIs for the health outcomes of ACEs (≥1 vs. 0) across CPCE exposure levels. All odds ratios are adjusted for age, sex, educational attainment, and FPCEs. The significance level of data in bold font is <0.05. *P value for the interaction effect of ACEs (≥1 vs. 0) × CPCEs (cumulative variable). †P value for interaction effect of ACEs (≥1 vs. 0) × CPCEs (≥3+ vs. 0–2).

Figure 2

Adjusted predictions with 95% CIs for the health outcomes of ACEs (≥1 vs. 0) × CPCEs (≥3 vs. 0–2). Substitutions were performed on the multiple logistic regression models presented in Supplementary Table S6. *P value for significant differences of marginal effects between 0–2 and ≥3 CPCEs.

For peer review only

For peer review only - http://bmjopen.bmj.com/site/about/guidelines.xhtml

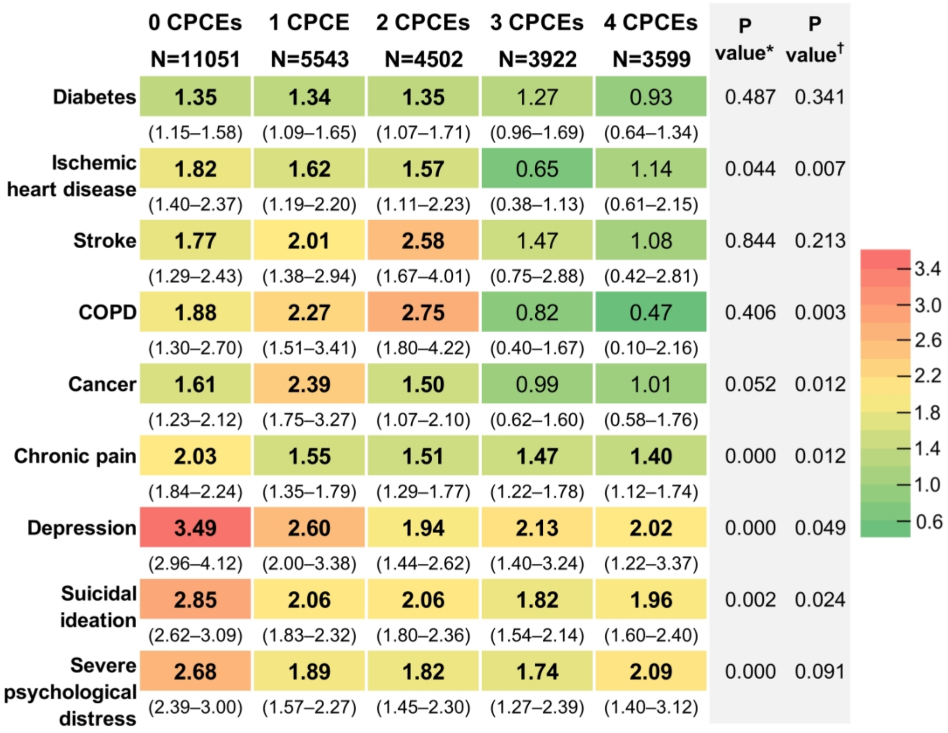

	0 CPCEs N=11051	1 CPCE N=5543	2 CPCEs N=4502	3 CPCEs N=3922	4 CPCEs N=3599	P value*	P value†
Diabetes	**1.35** (1.15–1.58)	**1.34** (1.09–1.65)	**1.35** (1.07–1.71)	1.27 (0.96–1.69)	0.93 (0.64–1.34)	0.487	0.341
Ischemic heart disease	**1.82** (1.40–2.37)	**1.62** (1.19–2.20)	**1.57** (1.11–2.23)	0.65 (0.38–1.13)	1.14 (0.61–2.15)	0.044	0.007
Stroke	**1.77** (1.29–2.43)	**2.01** (1.38–2.94)	**2.58** (1.67–4.01)	1.47 (0.75–2.88)	1.08 (0.42–2.81)	0.844	0.213
COPD	**1.88** (1.30–2.70)	**2.27** (1.51–3.41)	**2.75** (1.80–4.22)	0.82 (0.40–1.67)	0.47 (0.10–2.16)	0.406	0.003
Cancer	**1.61** (1.23–2.12)	**2.39** (1.75–3.27)	**1.50** (1.07–2.10)	0.99 (0.62–1.60)	1.01 (0.58–1.76)	0.052	0.012
Chronic pain	**2.03** (1.84–2.24)	**1.55** (1.35–1.79)	**1.51** (1.29–1.77)	**1.47** (1.22–1.78)	**1.40** (1.12–1.74)	0.000	0.012
Depression	**3.49** (2.96–4.12)	**2.60** (2.00–3.38)	**1.94** (1.44–2.62)	**2.13** (1.40–3.24)	**2.02** (1.22–3.37)	0.000	0.049
Suicidal ideation	**2.85** (2.62–3.09)	**2.06** (1.83–2.32)	**2.06** (1.80–2.36)	**1.82** (1.54–2.14)	**1.96** (1.60–2.40)	0.002	0.024
Severe psychological distress	**2.68** (2.39–3.00)	**1.89** (1.57–2.27)	**1.82** (1.45–2.30)	**1.74** (1.27–2.39)	**2.09** (1.40–3.12)	0.000	0.091

Figure 1A heatmap of the adjusted odds ratios with 95% CIs for the health outcomes of ACEs (≥1 vs. 0) across CPCE exposure levels. All odds ratios are adjusted for age, sex, educational attainment, and FPCEs. The significance level of data in bold font is <0.05. *P value for the interaction effect of ACEs (≥1 vs. 0) × CPCEs (cumulative variable). †P value for interaction effect of ACEs (≥1 vs. 0) × CPCEs (≥3+ vs. 0–2).

130x97mm (600 x 600 DPI)

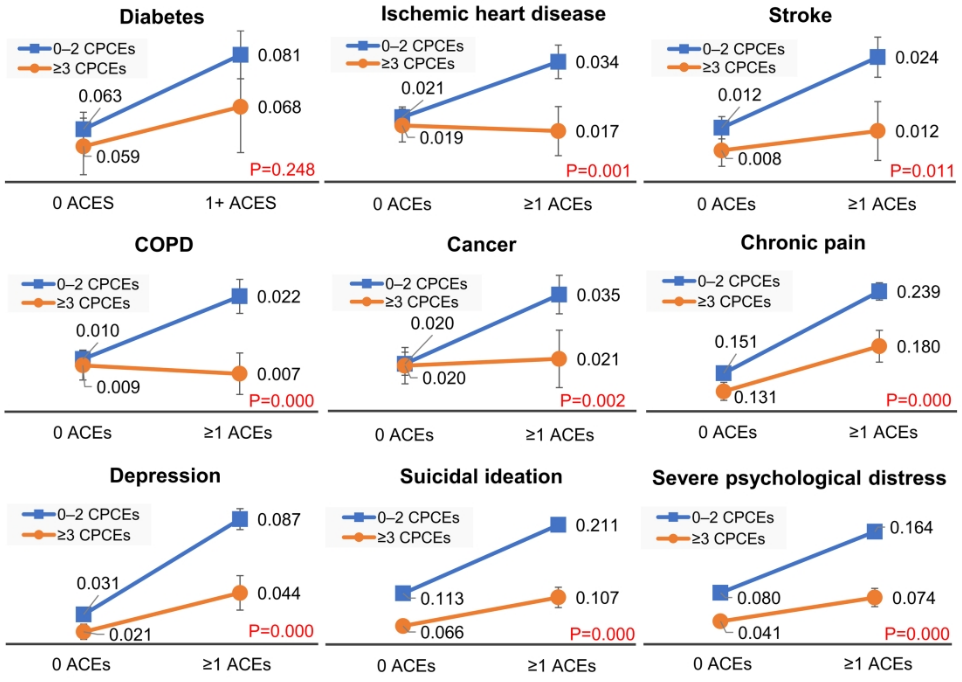

Figure 2 Adjusted predictions with 95% CIs for the health outcomes of ACEs (≥1 vs. 0) × CPCEs (≥3 vs. 0–2). Substitutions were performed on the multiple logistic regression models presented in Supplementary Table S6. *P value for significant differences of marginal effects between 0–2 and ≥3 CPCEs.

130x92mm (600 x 600 DPI)

Supplemental Material

Table S1 Prevalence of PCE single items among participants (N=28617)

	N	weighted %
FPCEs		
i) Felt able to talk to their family about feelings	9113	28.6
ii) Felt their family stood by them during difficult times	12738	40.0
iii) Felt safe and protected by an adult in their home	14700	45.8
CPCEs		
iv) Had at least 2 nonparent adults who took genuine interest in them	10732	32.8
v) Felt supported by friends	10563	32.1
vi) Felt a sense of belonging in middle and high school	12334	37.9
vii) Enjoyed participating in community traditions	7080	22.1

FPCEs, family positive childhood experiences; CPCEs, community positive childhood experiences

Table S2 Summary of results of psychometric analyses performed on FPCEs and CPCEs

	Internal Consistency (Cronbach's alpha)	Principle components factor analysis results	Factor loading across items
FPCEs	0.81	A single Eigen value greater than 1.0 (2.19) that explained 72.9% of variance	0.54 ("felt able to talk") to 0.61 ("felt safe and protected")
CPCEs	0.75	A single Eigen value greater than 1.0 (2.28) that explained 56.9% of variance	0.46 ("community traditions") to 0.52 ("felt supported by friends")

FPCEs, family positive childhood experiences; CPCEs, community positive childhood experiences

For peer review only

Table S3 Demographic characteristics and prevalence of health outcomes, PCEs by ACE exposure levels

	Prevalence of ACEs				
	0 ACEs	1 ACE	2–3 ACEs	≥4 ACEs	
	N=17889	N=5547	N=3725	N=1456	
	W %/M	W %/M	W %/M	W %/M	P value*
Health outcomes					
Diabetes	6.9	9.1	7.6	10.5	<0.001
Ischemic heart disease	2.7	2.8	3.0	6.8	<0.001
Stroke	1.2	1.4	2.3	5.6	<0.001
COPD	1.1	0.8	2.2	5.7	<0.001
Cancer	2.0	2.8	3.3	6.7	<0.001
Chronic pain	14.7	20.9	26.7	33.1	<0.001
Depression	2.8	3.9	8.6	17.7	<0.001
Suicidal ideation	10.3	13.3	25.7	35.7	<0.001
Severe psychological distress	7.0	8.9	19.0	30.6	<0.001
FPCEs (0–3)	1.3	1.1	0.7	0.3	<0.001
0 FPCE	39.9	47.7	65.0	80.2	<0.001
1 FPCEs	14.4	13.6	13.9	10.7	0.095
2 FPCEs	17.9	17.1	11.8	5.4	<0.001
3 FPCEs	27.8	21.7	9.4	3.7	<0.001
CPCEs (0–4)	1.4	1.2	0.9	0.7	<0.001
0 CPCE	40.4	43.9	52.8	61.8	<0.001
1 CPCEs	18.3	21.1	21.8	18.7	<0.001
2 CPCEs	14.9	15.7	13.0	12.6	0.002
3 CPCEs	13.3	10.4	8.0	4.4	<0.001
4 CPCEs	13.1	8.9	4.5	2.6	<0.001
Age (19–79)	47.4	51.3	47.2	45.2	<0.001
Sex					
Male	52.8	46.2	39.8	38.5	<0.001
Female	47.2	53.8	60.2	61.5	<0.001
Educational attainment					
Less than HS	2.9	7.5	5.0	9.0	<0.001
HS graduate	57.8	62.9	64.8	66.8	<0.001
Post-secondary and graduate	37.8	28.6	28.7	23.1	<0.001

*P value for significant differences by Pearson's χ^2 test or ANOVA.

W, weighted; ACEs, adverse childhood experiences; FPCEs, family positive childhood experiences; CPCEs, community positive childhood experiences; HS, high school

For peer review only

Table S4 Adjusted odds ratios of health outcomes by ACE exposure levels

	Diabetes		Ischemic heart disease		Stroke		COPD		Cancer		Chronic pain		Depression		Suicidal ideation		Severe psychological distress	
	**AOR**	**(95% CI)**	**AOR**	**(95% CI)**	**AOR**	**(95% CI)**	**AOR**	**(95% CI)**	**AOR**	**(95% CI)**	**AOR**	**(95% CI)**	**AOR**	**(95% CI)**	**AOR**	**(95% CI)**	**AOR**	**(95% CI)**
ACEs																		
0 ACEs [reference]	1.00		1.00		1.00		1.00		1.00		1.00		1.00		1.00		1.00	
1 ACE	1.16	(1.02–1.31)	1.08	(0.88–1.33)	1.24	(0.95–1.63)	0.98	(0.71–1.35)	1.06	(0.86–1.30)	1.41	(1.30–1.53)	1.75	(1.49–2.04)	1.65	(1.51–1.82)	1.65	(1.48–1.84)
2–3 ACEs	1.43	(1.24–1.65)	1.78	(1.44–2.21)	2.28	(1.76–2.95)	2.62	(2.01–3.41)	1.90	(1.55–2.34)	1.94	(1.78–2.11)	3.81	(3.30–4.40)	3.16	(2.88–3.47)	3.25	(2.93–3.61)
4 ACEs	1.98	(1.62–2.42)	3.52	(2.73–4.54)	5.21	(3.94–6.89)	5.66	(4.24–7.56)	3.71	(2.90–4.73)	2.93	(2.60–3.30)	8.12	(6.90–9.56)	4.29	(3.78–4.86)	5.94	(5.20–6.78)
Age	1.04	(1.04–1.05)	1.02	(1.01–1.02)	0.99	(0.99–1.00)	0.98	(0.97–0.99)	1.02	(1.02–1.03)	1.01	(1.01–1.01)	0.98	(0.98–0.98)	0.96	(0.96–0.97)	0.96	(0.96–0.96)
Sex																		
Male [reference]	1.00		1.00		1.00		1.00		1.00		1.00		1.00		1.00		1.00	
Female	0.31	(0.28–0.35)	0.34	(0.29–0.40)	0.38	(0.31–0.47)	0.32	(0.26–0.40)	0.73	(0.63–0.85)	1.13	(1.06–1.20)	0.62	(0.55–0.69)	1.02	(0.95–1.09)	0.88	(0.81–0.95)
Educational attainment																		
Less than HS	1.64	(1.22–2.20)	1.29	(0.80–2.07)	0.99	(0.52–1.90)	1.24	(0.74–2.45)	0.75	(0.43–1.32)	1.22	(0.98–1.51)	1.60	(1.17–2.19)	1.66	(1.32–2.09)	1.55	(1.20–1.99)
HS graduate	1.13	(1.02–1.25)	1.05	(0.90–1.24)	1.04	(0.85–1.28)	0.99	(0.79–1.23)	0.82	(0.70–0.97)	1.11	(1.04–1.18)	1.11	(0.99–1.25)	1.08	(1.00–1.16)	1.02	(0.93–1.11)
Post-secondary and graduate [reference]	1.00		1.00		1.00		1.00		1.00		1.00		1.00		1.00		1.00	

AOR, adjusted odds ratio; ACEs, adverse childhood experiences; HS, high school

For peer review only

Table S5 Adjusted odds ratios of health outcomes by ACEs, FPCEs, and CPCEs

	**Diabetes**		**Ischemic heart disease**		**Stroke**		**COPD**		**Cancer**		**Chronic pain**		**Depression**		**Suicidal ideation**		**Severe psychological distress**	
	**AOR**	**(95% CI)**	**AOR**	**(95% CI)**	**AOR**	**(95% CI)**	**AOR**	**(95% CI)**	**AOR**	**(95% CI)**	**AOR**	**(95% CI)**	**AOR**	**(95% CI)**	**AOR**	**(95% CI)**	**AOR**	**(95% CI)**
ACEs																		
0 ACEs [reference]	1.00		1.00		1.00		1.00		1.00		1.00		1.00		1.00		1.00	
≥1 ACEs	1.30	(1.18–1.44)	1.52	(1.30–1.78)	1.92	(1.58–2.33)	1.95	(1.58–2.41)	1.60	(1.37–1.87)	1.70	(1.60–1.82)	2.85	(2.54–3.20)	2.12	(1.97–2.28)	2.28	(2.10–2.48)
FPCEs																		
0–1 FPCEs [reference]	1.00		1.00		1.00		1.00		1.00		1.00		1.00		1.00		1.00	
2 FPCEs	0.99	(0.86–1.15)	0.99	(0.80–1.24)	0.77	(0.59–1.01)	0.77	(0.58–1.02)	0.86	(0.69–1.06)	1.00	(0.91–1.10)	0.88	(0.74–1.06)	0.83	(0.74–0.93)	0.81	(0.71–0.93)
3 FPCEs	0.81	(0.73–0.98)	0.70	(0.55–0.90)	0.50	(0.37–0.70)	0.40	(0.28–0.58)	0.68	(0.54–0.85)	0.95	(0.87–1.05)	0.69	(0.56–0.84)	0.62	(0.55–0.70)	0.64	(0.56–0.74)
CPCEs																		
0–2 CPCEs [reference]	1.00		1.00		1.00		1.00		1.00		1.00		1.00		1.00		1.00	
3 CPCEs	0.96	(0.83–1.12)	0.80	(0.62–1.03)	0.63	(0.44–0.89)	0.76	(0.54–1.07)	0.82	(0.84–1.05)	0.82	(0.74–0.91)	0.62	(0.50–0.78)	0.56	(0.49–0.64)	0.52	(0.45–0.62)
4 CPCEs	0.79	(0.66–0.95)	0.65	(0.47–0.89)	0.52	(0.34–0.82)	0.38	(0.22–0.66)	0.80	(0.61–1.06)	0.75	(0.67–0.84)	0.55	(0.42–0.72)	0.45	(0.38–0.53)	0.40	(0.32–0.49)
Age	1.04	(1.04–1.04)	1.02	(1.01–1.02)	0.99	(0.98–1.00)	0.98	(0.97–0.98)	1.02	(1.01–1.02)	1.01	(1.01–1.01)	0.98	(0.98–0.98)	0.96	(0.96–0.96)	0.96	(0.95–0.96)
Sex																		
Male [reference]	1.00		1.00		1.00		1.00		1.00		1.00		1.00		1.00		1.00	
Female	0.32	(0.29–0.35)	0.36	(0.30–0.42)	0.41	(0.34–0.51)	0.35	(0.28–0.44)	0.75	(0.64–0.87)	1.17	(1.10–1.24)	0.70	(0.63–0.78)	1.17	(1.09–1.26)	1.02	(0.94–1.11)
Educational attainment																		
Less than HS	1.70	(1.27–2.28)	1.45	(0.90–2.33)	1.17	(0.62–2.23)	1.69	(0.93–3.06)	0.90	(0.51–1.57)	1.27	(1.03–1.58)	1.67	(1.23–2.28)	1.57	(1.25–1.97)	1.49	(1.16–1.91)
HS graduate	1.14	(1.03–1.26)	1.08	(0.92–1.27)	1.07	(0.87–1.31)	1.03	(0.82–1.28)	0.85	(0.73–1.00)	1.11	(1.05–1.19)	1.10	(0.98–1.23)	1.03	(0.95–1.11)	0.97	(0.89–1.06)
Post-secondary and graduate [reference]	1.00		1.00		1.00		1.00		1.00		1.00		1.00		1.00		1.00	

AOR, adjusted odds ratio; ACEs, adverse childhood experiences; FPCEs, family positive childhood experiences; CPCEs, community positive childhood; HS, high school

For peer review only

Table S6 Adjusted odds ratios of health outcomes by ACEs, CPCEs, and these interaction terms

	Diabetes		Ischemic heart disease		Stroke		COPD		Cancer		Chronic pain		Depression		Suicidal ideation		Severe psychological distress	
	**AOR**	**(95% CI)**	**AOR**	**(95% CI)**	**AOR**	**(95% CI)**	**AOR**	**(95% CI)**	**AOR**	**(95% CI)**	**AOR**	**(95% CI)**	**AOR**	**(95% CI)**	**AOR**	**(95% CI)**	**AOR**	**(95% CI)**
ACEs																		
0 ACEs [reference]	1.00		1.00		1.00		1.00		1.00		1.00		1.00		1.00		1.00	
≥1 ACEs	1.34	(1.20–1.49)	1.68	(1.42–2.00)	2.02	(1.63–2.49)	2.24	(1.78–2.81)	1.77	(1.49–2.11)	1.78	(1.65–1.91)	2.99	(2.63–3.38)	2.20	(2.03–2.37)	2.35	(2.15–2.56)
CPCEs																		
0–2 CPCEs [reference]	1.00		1.00		1.00		1.00		1.00		1.00		1.00		1.00		1.00	
≥3 CPCEs	0.93	(0.79–1.08)	0.55	(0.70–1.16)	0.68	(0.47–0.98)	0.88	(0.61–1.27)	0.98	(0.77–1.25)	0.85	(0.77–0.94)	0.67	(0.52–0.85)	0.55	(0.47–0.63)	0.48	(0.41–0.57)
Interaction																		
≥1 ACEs × ≥3 CPCEs	0.89	(0.70–1.13)	0.50	(0.36–0.85)	0.70	(0.39–1.23)	0.37	(0.19–0.74)	0.61	(0.41–0.90)	0.82	(0.70–0.96)	0.71	(0.51–1.00)	0.79	(0.64–0.97)	0.80	(0.62–1.04)
Age	1.04	(1.04–1.05)	1.02	(1.01–1.02)	0.99	(0.98–1.00)	0.98	(0.97–0.99)	1.02	(1.01–1.02)	1.01	(1.01–1.01)	0.98	(0.98–0.98)	0.96	(0.96–0.96)	0.96	(0.95–0.96)
Sex																		
Male [reference]	1.00		1.00		1.00		1.00		1.00		1.00		1.00		1.00		1.00	
Female	0.32	(0.29–0.35)	0.35	(0.30–0.42)	0.41	(0.33–0.51)	0.35	(0.27–0.43)	0.74	(0.64–0.87)	1.17	(1.09–1.24)	0.69	(0.62–0.77)	1.15	(1.07–1.24)	1.01	(0.93–1.09)
Educational attainment																		
Less than HS	1.70	(1.27–2.27)	1.43	(0.89–2.30)	1.16	(0.61–2.22)	1.66	(0.91–3.01)	0.88	(0.50–1.55)	1.27	(1.02–1.57)	1.71	(1.26–2.33)	1.60	(1.28–2.01)	1.54	(1.20–1.98)
HS graduate	1.14	(1.03–1.26)	1.08	(0.92–1.27)	1.07	(0.87–1.31)	1.02	(0.82–1.27)	0.86	(0.73–1.00)	1.12	(1.05–1.19)	1.11	(0.99–1.24)	1.03	(0.96–1.12)	0.98	(0.90–1.07)
Post-secondary and graduate [reference]	1.00		1.00		1.00		1.00		1.00		1.00		1.00		1.00		1.00	
FPCEs																		
0–1 FPCEs [reference]	1.00		1.00		1.00		1.00		1.00		1.00		1.00		1.00		1.00	
2 FPCEs	0.99	(0.86–1.15)	0.99	(0.80–1.24)	0.77	(0.58–1.01)	0.76	(0.57–1.01)	0.86	(0.69–1.06)	1.00	(0.91–1.10)	0.79	(0.67–0.93)	0.75	(0.67–0.83)	0.68	(0.60–0.77)
3 FPCEs	0.83	(0.72–0.96)	0.68	(0.54–0.87)	0.49	(0.36–0.68)	0.36	(0.25–0.52)	0.67	(0.53–0.83)	0.94	(0.86–1.03)	0.61	(0.50–0.73)	0.55	(0.49–0.61)	0.53	(0.46–0.60)

AOR, adjusted odds ratio ACEs, adverse childhood experiences; CPCEs, community positive childhood; HS, high school; FPCEs, family positive childhood experiences

For peer review only

Table S7 Marginal effects with test of interaction effects

	**Marginal effects (95% CI)**		**Differences of two marginal effects (95% CI)**	P value*
	0–2 CPCEs	≥3 CPCEs		
Diabetes	0.018 (0.011 to 0.025)	0.010 (−0.003 to 0.022)	0.008 (−0.023 to 0.006)	0.248
Ischemic heart disease	0.013 (0.009 to 0.018)	−0.001 (−0.008 to 0.006)	0.015 (0.006 to 0.023)	0.001
Stroke	0.012 (0.008 to 0.016)	0.003 (−0.002 to 0.009)	0.009 (0.006 to 0.023)	0.011
COPD	0.012 (0.008 to 0.016)	−0.002 (−0.006 to 0.003)	0.014 (0.008 to 0.020)	0.000
Cancer	0.015 (0.010 to 0.020)	0.001 (−0.006 to 0.009)	0.013 (0.005 to 0.022)	0.002
Chronic pain	0.088 (0.077 to 0.099)	0.049 (0.030 to 0.068)	0.039 (0.017 to 0.061)	0.000
Depression	0.055 (0.049 to 0.062)	0.023 (0.012 to 0.033)	0.033 (0.020 to 0.046)	0.000
Suicidal ideation	0.098 (0.088 to 0.108)	0.041 (0.025 to 0.057)	0.057 (0.038 to 0.076)	0.000
Severe psychological distress	0.084 (0.075 to 0.092)	0.033 (0.019 to 0.046)	0.051 (0.035 to 0.067)	0.000

*P value for significant differences by test of the equality of marginal effects (Stata: margins, contrast).

For peer review only

STROBE Statement—Checklist of items that should be included in reports of ***cross-sectional studies***

	Item No	Recommendation	Page No
**Title and abstract**	1	(*a*) Indicate the study's design with a commonly used term in the title or the abstract	1-2
		(*b*) Provide in the abstract an informative and balanced summary of what was done and what was found	2
**Introduction**			
Background/rationale	2	Explain the scientific background and rationale for the investigation being reported	4
Objectives	3	State specific objectives, including any prespecified hypotheses	5
**Methods**			
Study design	4	Present key elements of study design early in the paper	4-5
Setting	5	Describe the setting, locations, and relevant dates, including periods of recruitment, exposure, follow-up, and data collection	6
Participants	6	(*a*) Give the eligibility criteria, and the sources and methods of selection of participants	6
Variables	7	Clearly define all outcomes, exposures, predictors, potential confounders, and effect modifiers. Give diagnostic criteria, if applicable	7-9
Data sources/ measurement	8*	For each variable of interest, give sources of data and details of methods of assessment (measurement). Describe comparability of assessment methods if there is more than one group	*7-9*
Bias	9	Describe any efforts to address potential sources of bias	6
Study size	10	Explain how the study size was arrived at	6
Quantitative variables	11	Explain how quantitative variables were handled in the analyses. If applicable, describe which groupings were chosen and why	7-9
Statistical methods	12	(*a*) Describe all statistical methods, including those used to control for confounding	9
		(*b*) Describe any methods used to examine subgroups and interactions	9
		(*c*) Explain how missing data were addressed	6
		(*d*) If applicable, describe analytical methods taking account of sampling strategy	9
		(*e*) Describe any sensitivity analyses	9
**Results**			
Participants	13*	(a) Report numbers of individuals at each stage of study—eg numbers potentially eligible, examined for eligibility, confirmed eligible, included in the study, completing follow-up, and analysed	6
		(b) Give reasons for non-participation at each stage	6
		(c) Consider use of a flow diagram	6
Descriptive data	14*	(a) Give characteristics of study participants (eg demographic, clinical, social) and information on exposures and potential confounders	9-11
		(b) Indicate number of participants with missing data for each variable of interest	9-11
Outcome data	15*	Report numbers of outcome events or summary measures	10
Main results	16	(*a*) Give unadjusted estimates and, if applicable, confounder-adjusted estimates and their precision (eg, 95% confidence interval). Make clear which confounders were adjusted for and why they were included	11-13

For peer review only - http://bmjopen.bmj.com/site/about/guidelines.xhtml

		(*b*) Report category boundaries when continuous variables were categorized	10-12
		(*c*) If relevant, consider translating estimates of relative risk into absolute risk for a meaningful time period	11-13
Other analyses	17	Report other analyses done—eg analyses of subgroups and interactions, and sensitivity analyses	12-13
**Discussion**			
Key results	18	Summarise key results with reference to study objectives	13
Limitations	19	Discuss limitations of the study, taking into account sources of potential bias or imprecision. Discuss both direction and magnitude of any potential bias	15
Interpretation	20	Give a cautious overall interpretation of results considering objectives, limitations, multiplicity of analyses, results from similar studies, and other relevant evidence	13-15
Generalisability	21	Discuss the generalisability (external validity) of the study results	13-15
**Other information**			
Funding	22	Give the source of funding and the role of the funders for the present study and, if applicable, for the original study on which the present article is based	17

*Give information separately for exposed and unexposed groups.

Note: An Explanation and Elaboration article discusses each checklist item and gives methodological background and published examples of transparent reporting. The STROBE checklist is best used in conjunction with this article (freely available on the Web sites of PLoS Medicine at http://www.plosmedicine.org/, Annals of Internal Medicine at http://www.annals.org/, and Epidemiology at http://www.epidem.com/). Information on the STROBE Initiative is available at www.strobe-statement.org.

For peer review only

For peer review only - http://bmjopen.bmj.com/site/about/guidelines.xhtml

BMJ Open

Promotive and protective effects of community-related positive childhood experiences on adult health outcomes in the context of adverse childhood experiences: a nationwide cross-sectional survey in Japan

Journal:	*BMJ Open*
Manuscript ID	bmjopen-2023-082134.R2
Article Type:	Original research
Date Submitted by the Author:	22-Mar-2024
Complete List of Authors:	Mitani, Haruyo; Osaka University, Graduate School of Human Sciences<br>Kondo, Naoki; Kyoto University, Graduate School of Medicine and School of Public Health, Department of Social Epidemiology<br>Amemiya, Airi; Kyoto University, Graduate School of Medicine and School of Public Health, Department of Social Epidemiology (former affiliation)<br>Tabuchi, Takahiro; Osaka International Cancer Institute, Cancer Control Center; Tohoku University, Graduate School of Medicine, School of Public Health, Division of Epidemiology
**Primary Subject Heading**:	Epidemiology
Secondary Subject Heading:	Communication, Epidemiology, Mental health, Sociology
Keywords:	Chronic Disease, EPIDEMIOLOGY, MENTAL HEALTH, Social Interaction, Stress, Psychological

SCHOLARONE™
Manuscripts

I, the Submitting Author has the right to grant and does grant on behalf of all authors of the Work (as defined in the below author licence), an exclusive licence and/or a non-exclusive licence for contributions from authors who are: i) UK Crown employees; ii) where BMJ has agreed a CC-BY licence shall apply, and/or iii) in accordance with the terms applicable for US Federal Government officers or employees acting as part of their official duties; on a worldwide, perpetual, irrevocable, royalty-free basis to BMJ Publishing Group Ltd ("BMJ") its licensees and where the relevant Journal is co-owned by BMJ to the co-owners of the Journal, to publish the Work in this journal and any other BMJ products and to exploit all rights, as set out in our licence.

The Submitting Author accepts and understands that any supply made under these terms is made by BMJ to the Submitting Author unless you are acting as an employee on behalf of your employer or a postgraduate student of an affiliated institution which is paying any applicable article publishing charge ("APC") for Open Access articles. Where the Submitting Author wishes to make the Work available on an Open Access basis (and intends to pay the relevant APC), the terms of reuse of such Open Access shall be governed by a Creative Commons licence – details of these licences and which Creative Commons licence will apply to this Work are set out in our licence referred to above.

Other than as permitted in any relevant BMJ Author's Self Archiving Policies, I confirm this Work has not been accepted for publication elsewhere, is not being considered for publication elsewhere and does not duplicate material already published. I confirm all authors consent to publication of this Work and authorise the granting of this licence.

For peer review only

Title

Promotive and protective effects of community-related positive childhood experiences on adult health outcomes in the context of adverse childhood experiences: a nationwide cross-sectional survey in Japan

Authors

Haruyo Mitani[1], Naoki Kondo[2], Airi Amemiya, Takahiro Tabuchi[3,4]

Author Affiliations

[1]Graduate School of Human Sciences, Osaka University, Osaka, Japan

[2]Department of Social Epidemiology, Graduate School of Medicine and School of Public Health, Kyoto University, Kyoto, Japan

[3]Cancer Control Center, Osaka International Cancer Institute, Osaka, Japan

[4]Division of Epidemiology, School of Public Health, Graduate School of Medicine, Tohoku University, Sendai, Japan

Correspondence to

Haruyo Mitani, Graduate School of Human Sciences, Osaka University, Osaka, Japan

haruyo.mitani@gmail.com

For peer review only

For peer review only - http://bmjopen.bmj.com/site/about/guidelines.xhtml

ABSTRACT

Objective: Although adverse childhood experiences (ACEs) are associated with poor health in adulthood, positive childhood experiences (PCEs) can reduce the risk of negative health outcomes. This study aimed to investigate whether PCEs in the community (CPCEs, i.e., trusted adults other than parents, supportive friends, belongingness to school, or community traditions) would have an independent effect on better health outcomes and moderate the association between ACEs and adult illnesses.

Design: Cross-sectional survey

Setting: Data were gathered from a nationwide, cross-sectional Internet survey conducted in Japan in 2022.

Participants: This study included 28,617 Japanese adults aged 18–82 years (51.1% female; mean age = 48.1 years).

Primary and secondary outcome measures: The associations among self-reported ACEs, CPCEs before the age of 18 years, and current chronic diseases (e.g., cancer and depression) were investigated using multivariable logistic regression models.

Results: CPCEs were associated with lower odds of adult diseases (such as stroke, chronic obstructive pulmonary disease [COPD], chronic pain, depression, suicidal ideation, and severe psychological distress) after adjusting for ACEs. More CPCEs weakened the association between ACEs and adult diseases. Specifically, among those with ACEs, ≥3 CPCEs (vs. 0–2 CPCEs) lowered the adjusted prevalence by ≥50% for stroke (2.4→1.2%), COPD (2.2→0.7%), and severe psychological distress (16.4→7.4%).

Conclusion: CPCEs could reduce ACE-related risk of poor physical and mental health in later

For peer review only - http://bmjopen.bmj.com/site/about/guidelines.xhtml

life. Early-life interventions that enhance PCEs in schools and/or neighborhoods are recommended.

Strengths and limitations of this study

- This study used a large and representative sample from a nationwide survey in Japan.
- Multivariable logistic regression models were used to adjust for possible confounders and to evaluate the associations among ACEs, CPCEs, and adult diseases.
- Due to the self-reported and retrospective nature of the survey data, the possibility of recall bias cannot be eliminated.
- A cross-sectional design limited our ability to determine causality.
- The CPCEs measure used in this study did not include all CPCEs.

For peer review only

For peer review only - http://bmjopen.bmj.com/site/about/guidelines.xhtml

INTRODUCTION

Over the last quarter-century, a growing body of research has demonstrated the harmful effects of adverse childhood experiences (ACEs), such as maltreatment and exposure to household dysfunction, on long-term physical and mental health[1–6]. Studies have emphasized a dose-response relationship between an individual's ACE score and their risk of having poor health; that is, the greater the exposure to ACEs, the greater the risk of physical and mental illness in later life. Chronic childhood stress exposure can lead to dysregulation of stress response systems, including the hypothalamic-pituitary-adrenocortical axis and the sympathetic-adrenomedullary system, resulting in permanent changes in multiple organ systems, particularly the brain[7]. Epigenetic modifications of DNA could play a role in these changes, and the biological disruptions of brain circuits, other organs, and metabolic systems during sensitive development are thought to lead to an increased risk of various chronic diseases in adulthood[8].

Meanwhile, recent evidence shows a potential role of positive childhood experiences (PCEs). For example, continual early assistance from a reliable adult[9], an adult who felt safe and protected as a child[10], or PCEs inside and outside the home (cumulative scale consisting of seven items such as "able to talk to family about feelings" and "felt supported by friends")[11], moderates the association between ACEs and negative consequences, represented by poor mental health. In other words, PCEs have a moderating effect as a protective factor; that is, the harmful effects of ACEs can be smaller among individuals who report more PCEs than among those who report fewer PCEs [9–15].

Resilience refers to the latent or apparent capacity to successfully respond to situations that threaten an individual's functioning, survival, or development[12]. Based on the Resilience Theory[12], the socio-ecological system suggests that multiple systems (e.g., individuals,

For peer review only - http://bmjopen.bmj.com/site/about/guidelines.xhtml

families, and communities) complementarily influence youth development. Moreover, factors promoting resilient outcomes can be divided into individual (e.g., cognition, coping style, and self-esteem), family (e.g., a safe and supportive relationship with a caregiver), and community factors (e.g., positive relationships with friends and neighbors)[12–15]. Notably, the concept of PCEs includes both family and community factors. Little is understood about the potential of PCEs in the community (CPCEs) for countering the negative lifetime effects of ACEs.

The present study focused on the role of CPCEs (PCEs in the community outside the home). Compared to those with fewer ACEs, children exposed to a higher number of ACEs are less likely to report having adults in their lives who made them feel safe or met their basic needs, highlighting a deficit in positive childhood experiences (PCEs)[10]. In other words, those with more ACEs are less likely to have experienced PCEs in the home. Hence, continuous family intervention by professionals to change how caregivers relate to children is desirable to enrich family PCEs (FPCEs)[16]. However, many countries lack the infrastructure (staff, funds, and legal structure) to make such professional intervention possible, and Japan is no exception[17]. However, community interventions in schools and neighborhoods can easily and inexpensively positively impact the development of children exposed to ACEs. Clarifying the role of CPCEs is essential for understanding the effectiveness of community interventions.

Therefore, this study explored the effect of CPCEs on the negative consequences of ACEs, focusing on four items of the PCE scale[11], referenced in many studies, that are considered particularly present in the community (i.e., trusted adults other than parents, supportive friends, belongingness to school, and community traditions). Similar to the original ACE study[1], this study used a broad range of physical and mental illnesses as primary outcomes. In Japan, while ACEs influence physical illnesses in old age[18] and mental disorders in adults[19], the interplay between PCEs and ACEs on health remains unexplored. Stress is identified as a

For peer review only - http://bmjopen.bmj.com/site/about/guidelines.xhtml

contributing factor to adult diseases[20]. ACEs are considered stressors originating within the childhood home, whereas CPCEs are viewed as external factors that mitigate the impact of these stressors.

This study explored the associations among ACEs, CPCEs, and adult diseases. First, the relationship between ACEs and adult diseases was ascertained. Then, the Resilience Theory's three main models (the Compensatory Model, the Protective Factors Model, and the Challenge Model [12,21–23]) were examined.

The Compensatory (Promotive Factors) Model assumes that promotive/compensatory factors directly impact developmental outcomes independent of risk factors. For example, even after controlling for ACEs, PCEs have been reported to predict less post-traumatic syndrome disorder symptoms and stress in pregnant women[24] and less depression, stress, and sleep difficulties in adults[25]. While ACEs could still have a significant effect on the outcome, CPCEs themselves are thought to enhance health in adulthood.

The Protective Factors Model assumes that protective factors mitigate the relationship between risk factors and bad outcomes. Protective factors do not act independently but can interact with risk factors. Previous studies have shown that adults who protect or meet basic needs[10] and high PCE scores[11] were associated with less poor mental health, especially among those with ACEs. It has also been found that in those with ≥4 ACEs, having all childhood community resilience assets such as given opportunity, supportive friends, and role model (vs. none) lowered the prevalence of self-rated poor childhood health from 59.8% to 21.3%[26]. In situations where more CPCEs are reported, the negative impact of ACEs on adult health is likely to be smaller.

The Challenge Model assumes that the modest levels of risk factors protect against additional

For peer review only - http://bmjopen.bmj.com/site/about/guidelines.xhtml

exposures that could render people prone to unfavorable consequences. Moderate levels of ACEs, particularly when accompanied by more protective factors, may promote improved adult health, akin to inoculation[22]. Some studies did not support this model[21], but it has also been reported that PCE score had greater positive effects on adult health (such as body mass index [BMI] and sleep difficulties) among those with <4 ACEs than among those with ≥4 ACEs[25]. Therefore, it can be speculated whether moderate ACEs can lower the likelihood of adult illness among individuals reporting more CPCEs.

Thus, this study tested three hypotheses: CPCEs directly affect adult health (H1), the association between ACEs and adult diseases is weaker among people with more CPCEs (H2), and ACEs are negatively associated with adult disease among people with more CPCEs (H3).

METHODS

Sample

The Japan COVID-19 and Society Internet Survey (JACSIS)[27] was conducted in 2022 by a major Internet research agency (Rakuten Insight, Inc.) with around 2.2 million registered qualified panelists. Of the panelists, 224,389 males and females aged 15–79 were invited to participate in the survey based on a random sample stratified by sex, age, and all 47 prefectures. Participants were rewarded with “E-Points” for online shopping. Informed consent was obtained from all participants before the survey. The survey began on September 12, 2022, and ended on October 19, 2022. The survey was terminated after the target of 32,000 participants was reached.

This study was authorized by the Research Ethics Committee of the Osaka International

For peer review only - http://bmjopen.bmj.com/site/about/guidelines.xhtml

For peer review only

Cancer Institute (approved on June 14, 2022; approval No. 20084-8) and followed the Helsinki guidelines.

Participants who provided contradictory answers to the screening questions were excluded from the study. These included the following criteria: (i) invalid answers to the screening question (participants were asked to choose the second-last option, but they chose other options), (ii) positive responses to all items on drug use, (iii) positive answers for having all 20 chronic diseases, and (iv) a total household size of over 15 individuals. There were 3,370 respondents who gave inconsistent answers, leaving 28,630 individuals. Thirteen participants aged <18 were also excluded since ACEs and PCEs are answered by recalling experiences up to age 18. Finally, 28,617 adults were included. There were no significant differences in the key variables (ACEs, PCEs, and adult health outcomes) between 28,630 and 28,617 adults. Due to the nature of the online survey, there were no missing values.

Patient and public involvement

Neither patients nor the public were involved in this study.

Measurements

ACEs

Questions on ACEs were derived from the ACEs scale for the Japanese context[28]. Although this study was based on the original ACE study[1], ACE items included parental death, except the incarceration of household members, to fit the Japanese context. Respondents were questioned whether any of the following adversities had occurred in their households by age

For peer review only - http://bmjopen.bmj.com/site/about/guidelines.xhtml

18: (i) death of either parent; (ii) parental divorce or separation; (iii) mental illness of either parent; (iv) substance abuse in the household (e.g., parental addiction to alcohol and gambling); (v) mother treated violently (the father was violent with the mother); (vi) emotional abuse (my parent said hurtful things or insulted me); (vii) physical abuse (my parent beat and hurt me); (viii) sexual abuse (an adult sexually touched me); (ix) emotional neglect (I always felt suffocated because my parent did not respect my opinion); and (x) physical neglect (I did not receive necessary care, such as eating and dressing). The response categories “yes” and “no” were coded as dummy variables, and these 10 items were summed into ACE scores (0–10). This score was classified into two categories (0 vs. ≥1) because of their effectiveness in determining poor health, following those of previous studies[18,29] and the clarity of the semantic difference between "without ACEs" and "with ACEs."

PCEs

PCEs were assessed using seven items adapted from the PCE scale[11]. Participants answered whether they had experienced any of the following by the time they were 18 years old: (i) felt able to talk to their family about feelings; (ii) felt their family stood by them during difficult times; (iii) felt safe and protected by an adult in their home; (iv) had at least two non-parental adults who took genuine interest in them; (v) felt supported by friends; (vi) felt a sense of belonging in middle and high school; and (vii) enjoyed participating in community traditions. Although these questions were asked in Japanese, and some were modified from the original questions (the addition of “middle” in item 6), these seven items can be considered almost identical to the PCEs items used by Bethell et al.[11] Of these items, the first three were positioned as components of FPCEs and the others as CPCEs (Supplementary Table S1). The applicable items were summed to obtain the FPCE (0–3) and CPCE (0–4) scores. As the PCE

For peer review only

For peer review only - http://bmjopen.bmj.com/site/about/guidelines.xhtml

scale[11], principal component analyses revealed a single-component structure for each FPCE and CPCE scale. Cronbach's alpha was 0.81 and 0.75 for FPCEs and CPCEs, respectively (Supplementary Table S2).

Adult health outcomes

Participants answered whether they currently had the following diseases using a dichotomous response variable (yes/no): (i) diabetes, (ii) ischemic heart disease (angina pectoris or myocardial infarction), (iii) stroke (cerebral infarction or hemorrhage), (iv) chronic obstructive pulmonary disease (COPD), (v) cancer, (vi) chronic pain (e.g., back pain and headache lasting ≥3 months), and (vii) depression. Additionally, (viii) suicidal ideation was determined as a "yes" response to the question, "Have you had times when you have wanted to die in the last year?" Psychological distress was measured with the Japanese version[30] of the Kessler 6-item psychological distress scale (K6)[31], assessing depressed mood and anxiety. The total score ranged from 0 to 24 on a five-point scale (0–4). A score of ≥13 was considered indicative of (ix) serious psychological distress[32].

Covariates

The following background variables reported by participants were included as covariates, in line with previous studies[1,33,34]: age (continuous by year), sex (0 "male" or 1 "female"), and educational attainment (three dummy variables for "less than high school," "high school," and "post-secondary and graduate").

For peer review only - http://bmjopen.bmj.com/site/about/guidelines.xhtml

Statistical analysis

Demographic characteristics and prevalence rates of all health outcomes in the sample were examined. For H1, multivariable logistic regression analyses were conducted to consider the direct effect of CPCEs on adult diseases after adjusting for covariates, ACEs, and FPCEs. For H2 and H3, multiple logistic regression analyses, stratified by CPCE exposure levels, were performed to examine whether CPCEs moderated the link between ACEs and adult diseases, adjusting for covariates and FPCEs. Interactive effects and marginal predicted probabilities were estimated to assess the statistical significance of the moderating effects of CPCEs on the association between ACEs and adult diseases[35]. The extent to which CPCEs reduced the risk of each disease was also estimated.

The survey data were weighted to represent the Japanese population. Considering the possibility of multicollinearity, the variance inflation factor (VIF) for each explanatory variable was checked to ensure that it did not exceed 4.0[36]. Stata 17.0 MP (StataCorp LLC, Texas, USA) was used for all analyses.

RESULTS

Participant characteristics

Table 1 shows that chronic pain and COPD were the most prevalent (18.6%) and least prevalent (1.4%) of the nine health outcomes, respectively. Approximately 39.8% of the participants had experienced ACEs. Furthermore, 52.8% and 56.0% of participants had experienced one or more FPCEs and CPCEs, respectively. When ACEs were divided into four exposure levels[9,11], the more exposure to ACEs, the higher the prevalence of adult diseases, the

For peer review only - http://bmjopen.bmj.com/site/about/guidelines.xhtml

lower the exposure rates to the two types of PCEs, and the higher the proportion of females and those with lower education levels (Supplementary Table S3). Finally, dose-response relationships between ACE exposure levels and all adult diseases were observed (Supplementary Table S4).

Table 1 Demographic characteristics and prevalence of health outcomes, ACEs, and PCEs among participants (N = 28617)

	**N**	**W %/M (SD)**
Health outcomes		
Diabetes	1945	7.7
Ischemic heart disease	686	3.0
Stroke	433	1.7
COPD	381	1.4
Cancer	705	2.6
Chronic pain	5062	18.6
Depression	1381	4.7
Suicidal ideation	3909	14.4
Severe psychological distress	2881	10.4
ACEs (0–11)	28617	0.81 (1.4)
0 ACEs	17889	60.2
≥1 ACEs	10728	39.8
FPCEs (0–3)	28617	1.14 (1.2)
0 FPCE	12003	47.2
1 FPCEs	4133	14.0
2 FPCEs	5025	16.2
3 FPCEs	7456	22.7
CPCEs (0–4)	28617	1.25 (1.4)
0 CPCE	11051	44.0
1 CPCEs	5543	19.4
2 CPCEs	4502	14.7
3 CPCEs	3922	11.5
4 CPCEs	3599	10.5
Age (18–82 years)	28617	48.1 (17.2)
Sex		
Male	13993	48.9

For peer review only - http://bmjopen.bmj.com/site/about/guidelines.xhtml

Female	14624	51.1
Educational attainment		
Less than HS	493	4.4
HS graduate	10516	60.3
Post-secondary and graduate	17397	33.8

W, weighted; SD, standard deviation; ACEs, adverse childhood experiences; CPCEs, community positive childhood experiences; FPCEs, family positive childhood experiences; HS, high school

Health outcomes according to ACE, FPCE, and CPCE exposure

Table 2 shows the adjusted odds ratios (AORs) for the poor health associated with CPCEs. In all models, VIF values did not exceed the criterion value (4.0). CPCEs had significant negative direct effects on six adult diseases: stroke, COPD, chronic pain, depression, suicidal ideation, and severe psychological distress, after adjusting for covariates, ACEs, and FPCEs. This result supported H1, which states that CPCEs directly affect adult health, derived from the Compensatory Model. For example, the adjusted odds of stroke were 15% lower for each additional CPCE. The same trend was found when CPCEs were treated as categorical variables (Supplementary Table S5). Overall, CPCEs were particularly associated with lower odds of mental illness.

Table 2 Adjusted odds ratios of health outcomes by ACEs (≥1 vs. 0), FPCEs, and CPCEs

	ACEs		FPCEs		CPCEs	
	**AOR***	**(95% CI)**	**AOR†**	**(95% CI)**	**AOR‡**	**(95% CI)**
Diabetes	**1.31**	(1.18–1.44)	**0.93**	(0.89–0.99)	0.97	(0.93–1.02)
Ischemic heart disease	**1.53**	(1.31–1.80)	**0.89**	(0.82–0.97)	0.93	(0.87–1.01)
Stroke	**1.96**	(1.61–2.39)	**0.83**	(0.75–0.93)	**0.85**	(0.77–0.93)
COPD	**2.00**	(1.62–2.47)	**0.77**	(0.69–0.86)	**0.89**	(0.81–0.98)
Cancer	**1.62**	(1.39–1.90)	**0.89**	(0.82–0.97)	0.95	(0.89–1.02)

For peer review only

For peer review only - http://bmjopen.bmj.com/site/about/guidelines.xhtml

Chronic pain	**1.71**	(1.60–1.82)	0.98	(0.95–1.02)	**0.91**	(0.89–0.94)
Depression	**2.86**	(2.55–3.22)	**0.89**	(0.83–0.95)	**0.81**	(0.76–0.86)
Suicidal ideation	**2.12**	(1.97–2.28)	**0.84**	(0.81–0.88)	**0.77**	(0.74–0.80)
Severe psychological distress	**2.29**	(2.10–2.48)	**0.86**	(0.81–0.90)	**0.72**	(0.69–0.75)

N = 28617; Significance level of data in bold font <0.05.

*Adjusted for age, sex, educational attainment, FPCEs, and CPCEs.

†Adjusted for age, sex, educational attainment, ACEs, and CPCEs.

‡Adjusted for age, sex, educational attainment, ACEs, and FPCEs.

ACEs, adverse childhood experiences; FPCEs, family positive childhood experiences; CPCEs, community positive childhood experiences; AOR, adjusted odds ratio; CI, confidence interval.

Health outcomes of ACEs across CPCE exposure levels

As shown in Figure 1, the AORs declined as PCE exposure levels rose; the cells become greener to the right. Particularly, the AORs tended to be smaller when the CPCE scores were ≥3 than when they were 0–2. This value of 3 CPCEs is also consistent with the Aiken and West[37] method, which recommends that the cutoff value is close to the mean plus one standard deviation (mean1.25+sd1.4 = 2.65). Therefore, the interaction effects of ACEs and CPCEs (≥3 vs. 0–2) on all nine outcomes were examined (Supplementary Table S6). Although the rightmost column of Figure 1 shows that the regression coefficients on the interaction terms between any ACE and ≥3 CPCEs were significant at the 10% level for seven diseases, it is a better way to examine the predicted probability themselves and perform the test of the equality of marginal effects to determine whether significant interaction effects exist[35].

Figure 2 shows the predicted values for each disease obtained from the regression equations. The results of the marginal effect difference test (the red values of Figure 2 and Supplementary Table S7) showed significant differences in the marginal effects (the amount of change in predicted probability when ACEs exceed ≥1 from 0) between those with 0–2 CPCEs and those

For peer review only - http://bmjopen.bmj.com/site/about/guidelines.xhtml

with ≥3 CPCEs for eight diseases except diabetes. CPCEs moderated the association between ACEs and eight adult diseases. This result supported H2, which states that the association between ACEs and adult diseases is weaker among people with more CPCEs, derived from the Protective Factors Model. Specifically, in ischemic heart disease, the difference between the marginal effect of 0–2 CPCEs (0.034−0.21 = 0.013) and one of ≥3 CPCEs (0.017−0.019 = −0.001) was 0.015, significant at the 1% level. According to each marginal effect (Supplementary Table S7), the marginal effect for CPCEs ≥3 was negative for ischemic heart disease (−0.001, 95% CI −0.008 to 0.006) and COPD (−0. 002, 95% CI −0.006 to 0.003). A negative association between ACEs and adult disease in the context of reporting more CPCEs was suggested, but H3 was not statistically supported, as suggested by the Challenge Model.

From Figure 2, the differences in predicted morbidity by CPCEs among those with ACEs can also be captured as relative risk reduction. For example, the predicted prevalence of ischemic heart disease did not differ according to CPCE exposure among those without ACEs; however, among those with ACEs, it differed from 3.4% in those with 0–2 CPCEs to 1.7% in those with ≥3 CPCEs (relative risk: 1.725/3.400*100 = 50.794%, relative risk reduction: 100.0 −50.8 = 49.2%). The adjusted prevalence was reduced by almost ≥50% for ischemic heart disease (49.2%), stroke (51.9%), COPD (67.1%), depression (49.1%), and severe psychological distress (54.9%).

DISCUSSION

This study examined the associations among ACEs, CPCEs, and adult diseases using a population-based sample from Japan. Specifically, ACEs were associated with increased odds of adult diseases, while CPCEs directly affected adult illnesses as a promotive factor and

For peer review only

mitigated the adverse health effects of ACEs as a protective factor. The protective effect of CPCEs was particularly pronounced in stroke, COPD, and severe psychological distress, with associated relative risk reductions of 50% or more by three or more CPCEs in those with ACEs.

Consistent with earlier research[1,4,6], the results showed a dose-response connection between ACEs and all adult diseases evaluated. Although an association between ACEs and diseases during old age among Japanese individuals was previously reported[18], this appeared robust across a wide age range (18–82 years). The ACE items are based on the original ACE study[1]. Although this measure does not include the variety of traumatic experiences that participants may have, it is noteworthy that a dose-response relationship with adult health was found, as in many studies.

H1, based on the Compensatory Model, was supported for many of the diseases assessed in this study. CPCEs, independent of ACEs and FPCEs, were directly associated with lower odds of adult-onset illnesses (i.e., stroke, COPD, chronic pain, depression, suicidal ideation, and severe psychological distress). While previous studies have found direct effects of PCEs primarily on mental health[11,25,34], the present study adds a novel finding that CPCEs independently function as promotive factors for physical and mental health.

More importantly, this study found analytical support for H2 based on the Protective Model. CPCEs moderated the relationships between ACEs and adult diseases (i.e., ischemic heart disease, stroke, COPD, cancer, chronic pain, depression, suicidal ideation, and severe psychological distress), even after adjusting for FPCEs. Although the possible protective function of PCEs has been noted[9–11], the buffering effect may be due to family factors. Notably, the present study observed that CPCEs can independently play a protective role. Furthermore, extending research focusing on community assets moderating the association between ACEs and poor child health[26], this study suggests that the moderating effects of

For peer review only - http://bmjopen.bmj.com/site/about/guidelines.xhtml

CPCEs may spread to adulthood, indicating that the protective effect may be stronger in cumulative situations where three or more CPCEs are aligned. The associated relative risk reductions of physical illness (i.e., ischemic heart disease, stroke, and COPD) and mental illness (i.e., depression and severe psychological distress) in ACE survivors by almost more than half due to ≥3 CPCEs would be a non-negligible value. Whether this cutoff point is appropriate requires follow-up testing; however, the harm caused by ACEs may be mitigated in multiple relationships.

Moreover, the analysis yielded the finding that, for several diseases (i.e., ischemic heart disease and COPD), those with ACEs had lower prevalence rates than those without ACEs in situations reporting more CPCEs (Figure 2). Although the negative marginal effects of ACEs on these illnesses were not statistically significant, as the Challenge Model[22] of the Resilience Theory implies, moderate ACEs promote health when sufficient protective factors exist; that is, they act as immunization. Future studies should examine the generalizability of this phenomenon.

Why might CPCEs contribute to developing children and youth into healthier adults? Prior research argues that supportive adults other than parents may provide opportunities for children to elude life stressors, establish positive experiences in social connections, and feel a sense of having a place and social association[38]. Additionally, supportive close friendships promote resilience by developing constructive coping styles, encouraging efforts, providing supportive peer networks, and reducing inappropriate coping[39]. In interactions with non-family members at school and in the neighborhood, children exposed to ACEs may gain physical refuge from toxic stress and develop psychological and instrumental coping strategies. The role of CPCEs as a promoting and protective factor found in this study may have been discovered only in the context of Japan, where many local communities have self-governing organizations

For peer review only - http://bmjopen.bmj.com/site/about/guidelines.xhtml

and traditional events, and violence among residents and students is relatively rare. Future studies should examine whether the findings are generalizable to other countries besides Japan.

Strengths and limitations

To the best of our knowledge, this is the first study to suggest that CPCEs could be promotive and protective factors of health system resilience. A strength of this study is the use of a large and representative sample. Moreover, various indicators elucidated the complex relationships among ACEs, PCEs, and adult diseases.

However, this study had some limitations. First, the data were self-reported and retrospective, which could have been influenced by recall bias. Individuals with a disease may be more likely to recall ACEs than healthy individuals. However, asking participants to reflect on their growing environment from a balanced perspective by asking about PCEs may have reduced this recall bias. Second, because of the study's cross-sectional nature, the possibility of reverse causation between ACEs/PCEs and adult illnesses cannot be eliminated. This study could not allow for the determination of causality. Third, the CPCEs measure used in this study did not include all CPCEs. Other types of CPCEs, such as participation in non-traditional activities and self-realization experiences, should be included in developing the CPCEs scale. Fourth, confounding variables (including psychological traits like optimism and hometown regional features, which may be linked to adult diseases, ACEs, and CPCEs) might have gone unnoticed. Fifth, the extent of the positive effects of the CPCEs differed for each health outcome. Particularly, the role of CPCEs in mental illness was found to be robust; however, the validity of these effects should be carefully investigated by including other health outcomes. Sixth, this study could not address a variety of traumatic experiences (e.g., homelessness, bullying

For peer review only - http://bmjopen.bmj.com/site/about/guidelines.xhtml

For peer review only

victimization, and childhood illness) other than typical ACEs.

Policy, practice, and research implications

The findings suggest that community-based interventions can be effective in helping children who have experienced adversity become resilient adults. Public health, social welfare, and educational practices that enforce CPCEs might as well be promoted. Additionally, the content of specific community-level approaches depends on social and local contexts. Such interventions are desirable to incorporate the main components of the Healthy Outcomes from Positive Experiences framework: (i) supportive and nurturing relationships, (ii) safe and stable environments, (iii) opportunities for constructive social involvement and connection, and (iv) social and emotional capacities[40]. For example, The National Resource Center at Tufts Medical Center[41] supports the above framework, providing tools and training to help healthcare providers, social service providers, educators, and members of community-based organizations acquire essential knowledge and skills to integrate these elements into their work.

In Japan, school counselors[42] and school social workers[43] in public elementary and junior high schools are expected to become resilience resources under compulsory education. Additionally, teachers might be a good way to get training in PCEs and improved working environments to build trusting relationships with their students. In local communities, traditional events such as festivals, nature experiences, multigenerational exchange programs, and the recently popularized "*Kodomo Shokudo*" (cafeterias for children)[44] could also contribute to the children's resilience. Further research and practice are expected to explore effective approaches to promoting CPCEs, including historical and new engagements.

For peer review only - http://bmjopen.bmj.com/site/about/guidelines.xhtml

Conclusion

This study showed that CPCEs positively impacted adult health in the general population regardless of ACE exposure and mitigated the adverse consequences. The study demonstrates that CPCEs may contribute to the lifelong health status of ACE survivors. Therefore, further research is needed to investigate the impact mechanisms of CPCEs and improve the scale's accuracy while capturing diverse childhood adversities, as well as interventions to strengthen supportive communities.

Contributors: HM and AA conceptualized the study. HM conducted the analysis and drafted the first manuscript. NK and AA contributed intellectual content to the study design and interpretation of the findings. TT collected data, managed the project, and obtained funding. All authors critically reviewed the manuscript and approved the final manuscript. HM is the guarantor for this study.

Funding: This study (JACSIS2022) was supported by the Japan Society for the Promotion of Science (JSPS) KAKENHI Grants (21H04856, 20K10467, 20K19633, 20K13721), the JST Grant (JPMJPF2017), the Health Labor Sciences Research Grant (21HA2016), the grant for 2021-2022 Strategic Research Promotion (SK202116) of Yokohama City University, and the research program on "Using Health Metrics to Monitor and Evaluate the Impact of Health Policies," conducted at the Tokyo Foundation for Policy Research. HM was also supported by the JSPS KAKENHI Grant (21K13454) and the Ministry of Education, Culture, Sports, Science and Technology (MEXT) KAKENHI Grant (20H05805).

Competing interests: None declared.

For peer review only - http://bmjopen.bmj.com/site/about/guidelines.xhtml

Data availability statement: All de-identified data from participants reported in this study are available to interested researchers who submit data-sharing requests to the last author with a summary of the secondary analysis plan.

ORCID iDs:

Haruyo Mitani https://orcid.org/0000-0001-8882-5098

Naoki Kondo https://orcid.org/0000-0002-6425-6844

Airi Amemiya https://orcid.org/0000-0001-5319-9541

Takahiro Tabuchi http://orcid.org/0000-0002-1050-3125

REFERENCES

1 Felitti VJ, Anda RF, Nordenberg D, *et al.* Relationship of childhood abuse and household dysfunction to many of the leading causes of death in adults. The Adverse Childhood Experiences (ACE) Study. *Am J Prev Med* 1998;**14**:245–58. doi:10.1016/s0749-3797(98)00017-8

2 Anda RF, Felitti VJ, Bremner JD, *et al.* The enduring effects of abuse and related adverse experiences in childhood: a convergence of evidence from neurobiology and epidemiology. *Eur Arch Psychiatry Clin Neurosci* 2006;**256**:174–86. doi:10.1007/s00406-005-0624-4

3 Bellis MA, Hughes K, Ford K, *et al.* Life course health consequences and associated annual costs of adverse childhood experiences across Europe and North America: a systematic review and meta-analysis. *Lancet Public Heal* 2019;**4**:e517–28. doi:10.1016/S2468-2667(19)30145-8

4 Hughes K, Bellis MA, Hardcastle, A K, *et al.* The effect of multiple adverse childhood experiences on health: a systematic review and meta-analysis. *Lancet Public Heal* 2017;**2**:e356–66.https://doi.org/10.1016/S2468-2667(17)30118-4.

5 Norman RE, Byambaa M, De R, *et al.* The long-term health consequences of child physical abuse, emotional abuse, and neglect: a systematic review and meta-analysis. *Plos Med* 2012;**9**:1–31. doi:10.1371/journal.pmed.1001349

For peer review only - http://bmjopen.bmj.com/site/about/guidelines.xhtml

6 Petruccelli K, Davis J, Berman T. Adverse childhood experiences and associated health outcomes: a systematic review and meta-analysis. *Child Abus Negl* 2019;**97**:104127. doi:10.1016/j.chiabu.2019.104127

7 Oral R, Ramirez M, Coohey C, *et al.* Adverse childhood experiences and trauma informed care: the future of health care. *Pediatr Res* 2016;**79**:227–33. doi:10.1038/pr.2015.197

8 Shonkoff JP, Garner AS, Siegel BS, *et al.* The lifelong effects of early childhood adversity and toxic stress. *Pediatrics* 2012;**129**:e232–46. doi:10.1542/PEDS.2011-2663

9 Bellis MA, Hardcastle K, Ford K, *et al.* Does continuous trusted adult support in childhood impart life-course resilience against adverse childhood experiences - a retrospective study on adult health-harming behaviours and mental well-being. *BMC Psychiatry* 2017;**17**:110. doi:10.1186/S12888-017-1260-Z/TABLES/3

10 Crouch E, Radcliff E, Strompolis M, *et al.* Safe, stable, and nurtured: protective factors against poor physical and mental health outcomes following exposure to adverse childhood experiences (ACEs). *J Child Adolesc Trauma* 2019;**12**:165–73. doi:10.1007/S40653-018-0217-9/METRICS

11 Bethell C, Jones J, Gombojav N, *et al.* Positive childhood experiences and adult mental and relational health in a statewide sample: associations across adverse childhood experiences levels. *JAMA Pediatr* 2019;**173**:e193007. doi:10.1001/jamapediatrics.2019.3007

12 Masten AS, Cicchetti D. Resilience in development: progress and transformation. In: D. Cicchetti, ed. *Developmental Psychopathology, Risk, Resilience, and Intervention*. John Wiley & Sons, Inc. 2016. 271–333. doi:https://doi.org/10.1002/9781119125556.devpsy406

13 Gartland D, Riggs E, Muyeen S, *et al.* What factors are associated with resilient outcomes in children exposed to social adversity? a systematic review. *BMJ Open* 2019;**9**:e024870. doi:10.1136/BMJOPEN-2018-024870

14 Henson M, Sabo S, Trujillo A, *et al.* Identifying protective factors to promote health in american indian and alaska native adolescents: a literature review. *J Prim Prev* 2017;**38**:5–26. doi:10.1007/S10935-016-0455-2/METRICS

15 Okwori G. Role of individual, family, and community resilience in moderating effects of adverse childhood experiences on mental health among children. *J Dev Behav Pediatr*

For peer review only - http://bmjopen.bmj.com/site/about/guidelines.xhtml

2022;**43**:E452–62. doi:10.1097/DBP.0000000000001076

16 Molloy C, Beatson R, Harrop C, *et al.* Systematic review: effects of sustained nurse home visiting programs for disadvantaged mothers and children. *J Adv Nurs* 2021;**77**:147–61. doi:10.1111/JAN.14576

17 Isumi A, Doi S, Ochi M, *et al.* School- and community-level protective factors for resilience among chronically maltreated children in Japan. *Soc Psychiatry Psychiatr Epidemiol* 2023;**58**:477–88. doi:10.1007/S00127-022-02322-X/TABLES/5

18 Amemiya A, Fujiwara T, Shirai K, *et al.* Association between adverse childhood experiences and adult diseases in older adults: a comparative cross-sectional study in Japan and Finland. *BMJ Open* 2019;**9**:e024609. doi:10.1136/bmjopen-2018-024609

19 Fujiwara T, Kawakami N. Association of childhood adversities with the first onset of mental disorders in Japan: results from the World Mental Health Japan, 2002-2004. *J Psychiatr Res* 2011;**45**:481–7. doi:10.1016/j.jpsychires.2010.08.002

20 Cohen S, Gianaros PJ, Manuck SB. A stage model of stress and disease. *Perspect Psychol Sci* 2016;**11**:456–63. doi:10.1177/1745691616646305

21 Mueller KC, Carey MT. How positive and negative childhood experiences interact with resiliency theory and the general theory of crime in juvenile probationers. *Youth Violence Juv Justice* 2022;**21**:130–48. doi:10.1177/15412040221131278

22 Zimmerman MA. Resiliency theory: a strengths-based approach to research and practice for adolescent health. *Heal Educ Behav* 2013;**40**:381–3. doi:10.1177/1090198113493782

23 Oshri A, Duprey EK, Liu S, *et al.* ACEs and resilience: methodological and conceptual issues. In: Asmundson GJG, Afifi TO, eds. *Adverse Childhood Experiences: Using Evidence to Advance Research, Practice, Policy, and Prevention*. Academic Press 2020. 287–306.

24 Narayan AJ, Rivera LM, Bernstein RE, *et al.* Positive childhood experiences predict less psychopathology and stress in pregnant women with childhood adversity: a pilot study of the benevolent childhood experiences (BCEs) scale. *Child Abuse Negl* 2018;**78**:19–30. doi:10.1016/J.CHIABU.2017.09.022

25 Crandall AA, Miller JR, Cheung A, *et al.* ACEs and counter-ACEs: how positive and negative childhood experiences influence adult health. *Child Abuse Negl*

For peer review only - http://bmjopen.bmj.com/site/about/guidelines.xhtml

2019;**96**:104089. doi:10.1016/J.CHIABU.2019.104089

26 Bellis MA, Hughes K, Ford K, *et al.* Adverse childhood experiences and sources of childhood resilience: a retrospective study of their combined relationships with child health and educational attendance. *BMC Public Health* 2018;**18**:1–12. doi:10.1186/S12889-018-5699-8/FIGURES/1

27 JACSIS study. The Japan COVID-19 and Society Internet Survey. 2021.https://jacsis-study.jp/

28 Fujiwara T. Impact of adverse childhood experience on physical and mental health: a life-course epidemiology perspective. *Psychiatry Clin Neurosci* 2022;**76**:544–51. doi:10.1111/PCN.13464

29 Cheong E Von, Sinnott C, Dahly D, *et al.* Adverse childhood experiences (ACEs) and later-life depression: perceived social support as a potential protective factor. *BMJ Open* 2017;**7**:e013228. doi:10.1136/BMJOPEN-2016-013228

30 Furukawa TA, Kawakami N, Saitoh M, *et al.* The performance of the Japanese version of the K6 and K10 in the World Mental Health Survey Japan. *Int J Methods Psychiatr Res* 2008;**17**:152–8. doi:10.1002/mpr.257

31 Kessler RC, Andrews G, Colpe LJ, *et al.* Short screening scales to monitor population prevalences and trends in non-specific psychological distress. *Psychol Med* 2002;**32**:959–76. doi:10.1017/S0033291702006074

32 Kessler RC, Green JG, Gruber MJ, *et al.* Screening for serious mental illness in the general population with the K6 screening scale: results from the WHO World Mental Health (WMH) survey initiative. *Int J Methods Psychiatr Res* 2010;**19**:4–22. doi:10.1002/mpr.310

33 Metzler M, Merrick MT, Klevens J, *et al.* Adverse childhood experiences and life opportunities: shifting the narrative. *Child Youth Serv Rev* 2017;**72**:141–9. doi:10.1016/j.childyouth.2016.10.021

34 Kuhar M, Zager Kocjan G. Associations of adverse and positive childhood experiences with adult physical and mental health and risk behaviours in Slovenia. *Eur J Psychotraumatol* 2021;**12**:1924953. doi:10.1080/20008198.2021.1924953

35 Mize TD. Best practices for estimating, interpreting, and presenting nonlinear interaction effects. *Sociol Sci* 2019;**6**:81–117. doi:10.15195/V6.A4

For peer review only

For peer review only - http://bmjopen.bmj.com/site/about/guidelines.xhtml

36 Hair JF, Black B, Babin BJ, *et al.* *Multivariate Data Analysis: Global Edition*. 7th ed. Pearson Education 2009.

37 Aiken LS, West SG. *Multiple Regression: Testing and Interpreting Interactions*. Newbury Park, CA: Sage Publications 1991.

38 Rhodes JE, Spencer R, Keller TE, *et al.* A model for the influence of mentoring relationships on youth development. *J Community Psychol* 2006;**34**:691–707. doi:10.1002/JCOP.20124

39 Graber R, Turner R, Madill A. Best friends and better coping: facilitating psychological resilience through boys' and girls' closest friendships. *Br J Psychol* 2016;**107**:338–58. doi:10.1111/BJOP.12135

40 Sege RD, Harper Browne C. Responding to ACEs with HOPE: Health Outcomes from Positive Experiences. *Acad Pediatr* 2017;**17**:S79–85. doi:10.1016/J.ACAP.2017.03.007

41 The National Resource Center at Tufts Medical Center. Tufts HOPE: Healthy Outcomes from Positive Experiences. 2023.https://positiveexperience.org/

42 Nishio A, Kakimoto M, Horita R, *et al.* Compulsory educational mental health support system in Japan. *Pediatr Int* 2020;**62**:529–34. doi:10.1111/PED.14205

43 Kanazawa M. Current issues of school social work in Japan : from the historical relations between school, social work, and counseling. *Japanese J Soc Serv* 2009;**5**:159–71. doi:10.24469/JJSWE.5.0_159

44 Murayama H, Kurotani K, Tabuchi T. Social capital and the spread of children's cafeterias (kodomo shokudo) in Japan: an ecological analysis. *Asia-Pacific J public Heal* 2022;**34**:817–20. doi:10.1177/10105395221119416

Figure legends

Figure 1

A heatmap of the adjusted odds ratios with 95% CIs for the health outcomes of ACEs (≥1 vs. 0) across CPCE exposure levels. All odds ratios are adjusted for age, sex, educational attainment, and FPCEs. The significance level of data in bold font is <0.05. *P value for the

For peer review only

For peer review only - http://bmjopen.bmj.com/site/about/guidelines.xhtml

interaction effect of ACEs (≥1 vs. 0) × CPCEs (cumulative variable). †P value for interaction effect of ACEs (≥1 vs. 0) × CPCEs (≥3+ vs. 0–2).

Figure 2

Adjusted predictions with 95% CIs for the health outcomes of ACEs (≥1 vs. 0) × CPCEs (≥3 vs. 0–2). Substitutions were performed on the multiple logistic regression models presented in Supplementary Table S6. *P value for significant differences of marginal effects between 0–2 and ≥3 CPCEs.

For peer review only

For peer review only - http://bmjopen.bmj.com/site/about/guidelines.xhtml

	0 CPCEs N=11051	1 CPCE N=5543	2 CPCEs N=4502	3 CPCEs N=3922	4 CPCEs N=3599	P value*	P value†
**Diabetes**	**1.35** (1.15–1.58)	**1.34** (1.09–1.65)	**1.35** (1.07–1.71)	1.27 (0.96–1.69)	0.93 (0.64–1.34)	0.487	0.341
**Ischemic heart disease**	**1.82** (1.40–2.37)	**1.62** (1.19–2.20)	**1.57** (1.11–2.23)	0.65 (0.38–1.13)	1.14 (0.61–2.15)	0.044	0.007
**Stroke**	**1.77** (1.29–2.43)	**2.01** (1.38–2.94)	**2.58** (1.67–4.01)	1.47 (0.75–2.88)	1.08 (0.42–2.81)	0.844	0.213
**COPD**	**1.88** (1.30–2.70)	**2.27** (1.51–3.41)	**2.75** (1.80–4.22)	0.82 (0.40–1.67)	0.47 (0.10–2.16)	0.406	0.003
**Cancer**	**1.61** (1.23–2.12)	**2.39** (1.75–3.27)	**1.50** (1.07–2.10)	0.99 (0.62–1.60)	1.01 (0.58–1.76)	0.052	0.012
**Chronic pain**	**2.03** (1.84–2.24)	**1.55** (1.35–1.79)	**1.51** (1.29–1.77)	**1.47** (1.22–1.78)	**1.40** (1.12–1.74)	0.000	0.012
**Depression**	**3.49** (2.96–4.12)	**2.60** (2.00–3.38)	**1.94** (1.44–2.62)	**2.13** (1.40–3.24)	**2.02** (1.22–3.37)	0.000	0.049
**Suicidal ideation**	**2.85** (2.62–3.09)	**2.06** (1.83–2.32)	**2.06** (1.80–2.36)	**1.82** (1.54–2.14)	**1.96** (1.60–2.40)	0.002	0.024
**Severe psychological distress**	**2.68** (2.39–3.00)	**1.89** (1.57–2.27)	**1.82** (1.45–2.30)	**1.74** (1.27–2.39)	**2.09** (1.40–3.12)	0.000	0.091

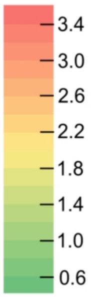

Figure 1

A heatmap of the adjusted odds ratios with 95% CIs for the health outcomes of ACEs (≥1 vs. 0) across CPCE exposure levels. All odds ratios are adjusted for age, sex, educational attainment, and FPCEs. The significance level of data in bold font is <0.05. *P value for the interaction effect of ACEs (≥1 vs. 0) × CPCEs (cumulative variable). †P value for interaction effect of ACEs (≥1 vs. 0) × CPCEs (≥3+ vs. 0–2).

130x97mm (1200 x 1200 DPI)

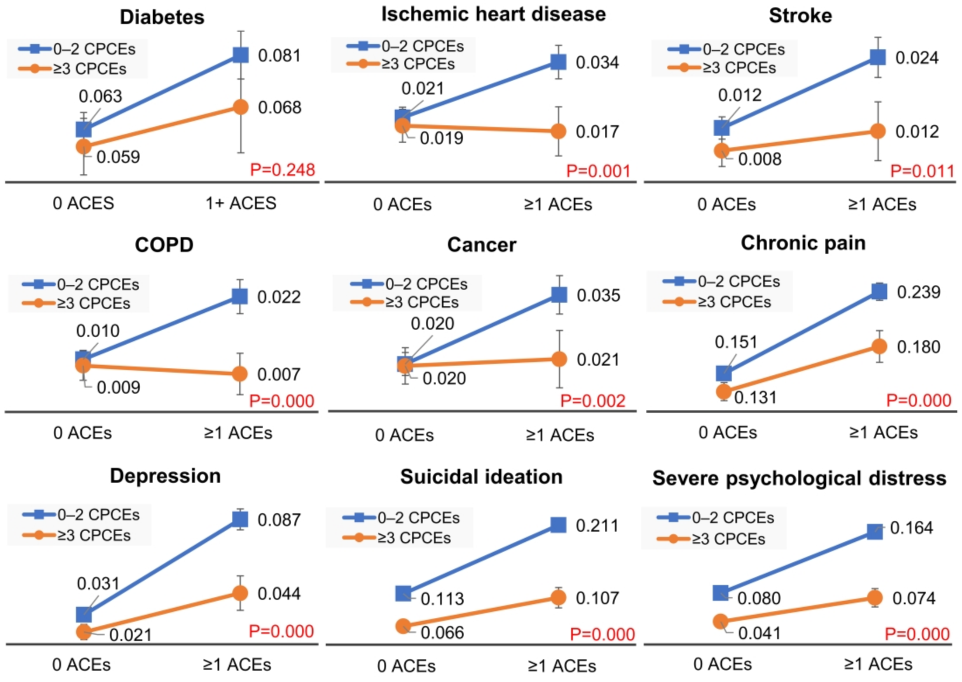

Figure 2

Adjusted predictions with 95% CIs for the health outcomes of ACEs (≥1 vs. 0) × CPCEs (≥3 vs. 0–2). Substitutions were performed on the multiple logistic regression models presented in Supplementary Table S6. *P value for significant differences of marginal effects between 0–2 and ≥3 CPCEs.

130x92mm (1200 x 1200 DPI)

Supplemental Material

Table S1 Prevalence of PCE single items among participants (N=28617)

	N	weighted %
FPCEs		
i) Felt able to talk to their family about feelings	9113	28.6
ii) Felt their family stood by them during difficult times	12738	40.0
iii) Felt safe and protected by an adult in their home	14700	45.8
CPCEs		
iv) Had at least 2 nonparent adults who took genuine interest in them	10732	32.8
v) Felt supported by friends	10563	32.1
vi) Felt a sense of belonging in middle and high school	12334	37.9
vii) Enjoyed participating in community traditions	7080	22.1

FPCEs, family positive childhood experiences; CPCEs, community positive childhood experiences

Table S2 Summary of results of psychometric analyses performed on FPCEs and CPCEs

	Internal Consistency (Cronbach's alpha)	Principle components factor analysis results	Factor loading across items
FPCEs	0.81	A single Eigen value greater than 1.0 (2.19) that explained 72.9% of variance	0.54 ("felt able to talk") to 0.61 ("felt safe and protected")
CPCEs	0.75	A single Eigen value greater than 1.0 (2.28) that explained 56.9% of variance	0.46 ("community traditions") to 0.52 ("felt supported by friends")

FPCEs, family positive childhood experiences; CPCEs, community positive childhood experiences

For peer review only

Table S3 Demographic characteristics and prevalence of health outcomes, PCEs by ACE exposure levels

	Prevalence of ACEs				
	**0 ACEs**	**1 ACE**	**2–3 ACEs**	**≥4 ACEs**	
	**N=17889**	**N=5547**	**N=3725**	**N=1456**	
	**W %/M**	**W %/M**	**W %/M**	**W %/M**	**P value***
Health outcomes					
Diabetes	6.9	9.1	7.6	10.5	<0.001
Ischemic heart disease	2.7	2.8	3.0	6.8	<0.001
Stroke	1.2	1.4	2.3	5.6	<0.001
COPD	1.1	0.8	2.2	5.7	<0.001
Cancer	2.0	2.8	3.3	6.7	<0.001
Chronic pain	14.7	20.9	26.7	33.1	<0.001
Depression	2.8	3.9	8.6	17.7	<0.001
Suicidal ideation	10.3	13.3	25.7	35.7	<0.001
Severe psychological distress	7.0	8.9	19.0	30.6	<0.001
FPCEs (0–3)	1.3	1.1	0.7	0.3	<0.001
0 FPCE	39.9	47.7	65.0	80.2	<0.001
1 FPCEs	14.4	13.6	13.9	10.7	0.095
2 FPCEs	17.9	17.1	11.8	5.4	<0.001
3 FPCEs	27.8	21.7	9.4	3.7	<0.001
CPCEs (0–4)	1.4	1.2	0.9	0.7	<0.001
0 CPCE	40.4	43.9	52.8	61.8	<0.001
1 CPCEs	18.3	21.1	21.8	18.7	<0.001
2 CPCEs	14.9	15.7	13.0	12.6	0.002
3 CPCEs	13.3	10.4	8.0	4.4	<0.001
4 CPCEs	13.1	8.9	4.5	2.6	<0.001
Age (19–79)	47.4	51.3	47.2	45.2	<0.001
Sex					
Male	52.8	46.2	39.8	38.5	<0.001
Female	47.2	53.8	60.2	61.5	<0.001
Educational attainment					
Less than HS	2.9	7.5	5.0	9.0	<0.001
HS graduate	57.8	62.9	64.8	66.8	<0.001
Post-secondary and graduate	37.8	28.6	28.7	23.1	<0.001

*P value for significant differences by Pearson's χ^2 test or ANOVA.

W, weighted; ACEs, adverse childhood experiences; FPCEs, family positive childhood experiences; CPCEs, community positive childhood experiences; HS, high school

For peer review only

Table S4 Adjusted odds ratios of health outcomes by ACE exposure levels

	Diabetes		Ischemic heart disease		Stroke		COPD		Cancer		Chronic pain		Depression		Suicidal ideation		Severe psychological distress	
	**AOR**	**(95% CI)**	**AOR**	**(95% CI)**	**AOR**	**(95% CI)**	**AOR**	**(95% CI)**	**AOR**	**(95% CI)**	**AOR**	**(95% CI)**	**AOR**	**(95% CI)**	**AOR**	**(95% CI)**	**AOR**	**(95% CI)**
ACEs																		
0 ACEs [reference]	1.00		1.00		1.00		1.00		1.00		1.00		1.00		1.00		1.00	
1 ACE	1.16	(1.02–1.31)	1.08	(0.88–1.33)	1.24	(0.95–1.63)	0.98	(0.71–1.35)	1.06	(0.86–1.30)	1.41	(1.30–1.53)	1.75	(1.49–2.04)	1.65	(1.51–1.82)	1.65	(1.48–1.84)
2–3 ACEs	1.43	(1.24–1.65)	1.78	(1.44–2.21)	2.28	(1.76–2.95)	2.62	(2.01–3.41)	1.90	(1.55–2.34)	1.94	(1.78–2.11)	3.81	(3.30–4.40)	3.16	(2.88–3.47)	3.25	(2.93–3.61)
4 ACEs	1.98	(1.62–2.42)	3.52	(2.73–4.54)	5.21	(3.94–6.89)	5.66	(4.24–7.56)	3.71	(2.90–4.73)	2.93	(2.60–3.30)	8.12	(6.90–9.56)	4.29	(3.78–4.86)	5.94	(5.20–6.78)
Age	1.04	(1.04–1.05)	1.02	(1.01–1.02)	0.99	(0.99–1.00)	0.98	(0.97–0.99)	1.02	(1.02–1.03)	1.01	(1.01–1.01)	0.98	(0.98–0.98)	0.96	(0.96–0.97)	0.96	(0.96–0.96)
Sex																		
Male [reference]	1.00		1.00		1.00		1.00		1.00		1.00		1.00		1.00		1.00	
Female	0.31	(0.28–0.35)	0.34	(0.29–0.40)	0.38	(0.31–0.47)	0.32	(0.26–0.40)	0.73	(0.63–0.85)	1.13	(1.06–1.20)	0.62	(0.55–0.69)	1.02	(0.95–1.09)	0.88	(0.81–0.95)
Educational attainment																		
Less than HS	1.64	(1.22–2.20)	1.29	(0.80–2.07)	0.99	(0.52–1.90)	1.24	(0.74–2.45)	0.75	(0.43–1.32)	1.22	(0.98–1.51)	1.60	(1.17–2.19)	1.66	(1.32–2.09)	1.55	(1.20–1.99)
HS graduate	1.13	(1.02–1.25)	1.05	(0.90–1.24)	1.04	(0.85–1.28)	0.99	(0.79–1.23)	0.82	(0.70–0.97)	1.11	(1.04–1.18)	1.11	(0.99–1.25)	1.08	(1.00–1.16)	1.02	(0.93–1.11)
Post-secondary and graduate [reference]	1.00		1.00		1.00		1.00		1.00		1.00		1.00		1.00		1.00	

AOR, adjusted odds ratio; ACEs, adverse childhood experiences; HS, high school

For peer review only

Table S5 Adjusted odds ratios of health outcomes by ACEs, FPCEs, and CPCEs

	Diabetes		Ischemic heart disease		Stroke		COPD		Cancer		Chronic pain		Depression		Suicidal ideation		Severe psychological distress	
	AOR	(95% CI)	AOR	(95% CI)	AOR	(95% CI)	AOR	(95% CI)	AOR	(95% CI)	AOR	(95% CI)	AOR	(95% CI)	AOR	(95% CI)	AOR	(95% CI)
ACEs																		
0 ACEs [reference]	1.00		1.00		1.00		1.00		1.00		1.00		1.00		1.00		1.00	
≥1 ACEs	1.30	(1.18–1.44)	1.52	(1.30–1.78)	1.92	(1.58–2.33)	1.95	(1.58–2.41)	1.60	(1.37–1.87)	1.70	(1.60–1.82)	2.85	(2.54–3.20)	2.12	(1.97–2.28)	2.28	(2.10–2.48)
FPCEs																		
0–1 FPCEs [reference]	1.00		1.00		1.00		1.00		1.00		1.00		1.00		1.00		1.00	
2 FPCEs	0.99	(0.86–1.15)	0.99	(0.80–1.24)	0.77	(0.59–1.01)	0.77	(0.58–1.02)	0.86	(0.69–1.06)	1.00	(0.91–1.10)	0.88	(0.74–1.06)	0.83	(0.74–0.93)	0.81	(0.71–0.93)
3 FPCEs	0.81	(0.73–0.98)	0.70	(0.55–0.90)	0.50	(0.37–0.70)	0.40	(0.28–0.58)	0.68	(0.54–0.85)	0.95	(0.87–1.05)	0.69	(0.56–0.84)	0.62	(0.55–0.70)	0.64	(0.56–0.74)
CPCEs																		
0–2 CPCEs [reference]	1.00		1.00		1.00		1.00		1.00		1.00		1.00		1.00		1.00	
3 CPCEs	0.96	(0.83–1.12)	0.80	(0.62–1.03)	0.63	(0.44–0.89)	0.76	(0.54–1.07)	0.82	(0.84–1.05)	0.82	(0.74–0.91)	0.62	(0.50–0.78)	0.56	(0.49–0.64)	0.52	(0.45–0.62)
4 CPCEs	0.79	(0.66–0.95)	0.65	(0.47–0.89)	0.52	(0.34–0.82)	0.38	(0.22–0.66)	0.80	(0.61–1.06)	0.75	(0.67–0.84)	0.55	(0.42–0.72)	0.45	(0.38–0.53)	0.40	(0.32–0.49)
Age	1.04	(1.04–1.04)	1.02	(1.01–1.02)	0.99	(0.98–1.00)	0.98	(0.97–0.98)	1.02	(1.01–1.02)	1.01	(1.01–1.01)	0.98	(0.98–0.98)	0.96	(0.96–0.96)	0.96	(0.95–0.96)
Sex																		
Male [reference]	1.00		1.00		1.00		1.00		1.00		1.00		1.00		1.00		1.00	
Female	0.32	(0.29–0.35)	0.36	(0.30–0.42)	0.41	(0.34–0.51)	0.35	(0.28–0.44)	0.75	(0.64–0.87)	1.17	(1.10–1.24)	0.70	(0.63–0.78)	1.17	(1.09–1.26)	1.02	(0.94–1.11)
Educational attainment																		
Less than HS	1.70	(1.27–2.28)	1.45	(0.90–2.33)	1.17	(0.62–2.23)	1.69	(0.93–3.06)	0.90	(0.51–1.57)	1.27	(1.03–1.58)	1.67	(1.23–2.28)	1.57	(1.25–1.97)	1.49	(1.16–1.91)
HS graduate	1.14	(1.03–1.26)	1.08	(0.92–1.27)	1.07	(0.87–1.31)	1.03	(0.82–1.28)	0.85	(0.73–1.00)	1.11	(1.05–1.19)	1.10	(0.98–1.23)	1.03	(0.95–1.11)	0.97	(0.89–1.06)
Post-secondary and graduate [reference]	1.00		1.00		1.00		1.00		1.00		1.00		1.00		1.00		1.00	

AOR, adjusted odds ratio; ACEs, adverse childhood experiences; FPCEs, family positive childhood experiences; CPCEs, community positive childhood; HS, high school

For peer review only - http://bmjopen.bmj.com/site/about/guidelines.xhtml

Table S6 Adjusted odds ratios of health outcomes by ACEs, CPCEs, and these interaction terms

	**Diabetes**		**Ischemic heart disease**		**Stroke**		**COPD**		**Cancer**		**Chronic pain**		**Depression**		**Suicidal ideation**		**Severe psychological distress**	
	**AOR**	**(95% CI)**	**AOR**	**(95% CI)**	**AOR**	**(95% CI)**	**AOR**	**(95% CI)**	**AOR**	**(95% CI)**	**AOR**	**(95% CI)**	**AOR**	**(95% CI)**	**AOR**	**(95% CI)**	**AOR**	**(95% CI)**
ACEs																		
0 ACEs [reference]	1.00		1.00		1.00		1.00		1.00		1.00		1.00		1.00		1.00	
≥1 ACEs	1.34	(1.20–1.49)	1.68	(1.42–2.00)	2.02	(1.63–2.49)	2.24	(1.78–2.81)	1.77	(1.49–2.11)	1.78	(1.65–1.91)	2.99	(2.63–3.38)	2.20	(2.03–2.37)	2.35	(2.15–2.56)
CPCEs																		
0–2 CPCEs [reference]	1.00		1.00		1.00		1.00		1.00		1.00		1.00		1.00		1.00	
≥3 CPCEs	0.93	(0.79–1.08)	0.55	(0.70–1.16)	0.68	(0.47–0.98)	0.88	(0.61–1.27)	0.98	(0.77–1.25)	0.85	(0.77–0.94)	0.67	(0.52–0.85)	0.55	(0.47–0.63)	0.48	(0.41–0.57)
Interaction																		
≥1 ACEs × ≥3 CPCEs	0.89	(0.70–1.13)	0.50	(0.36–0.85)	0.70	(0.39–1.23)	0.37	(0.19–0.74)	0.61	(0.41–0.90)	0.82	(0.70–0.96)	0.71	(0.51–1.00)	0.79	(0.64–0.97)	0.80	(0.62–1.04)
Age	1.04	(1.04–1.05)	1.02	(1.01–1.02)	0.99	(0.98–1.00)	0.98	(0.97–0.99)	1.02	(1.01–1.02)	1.01	(1.01–1.01)	0.98	(0.98–0.98)	0.96	(0.96–0.96)	0.96	(0.95–0.96)
Sex																		
Male [reference]	1.00		1.00		1.00		1.00		1.00		1.00		1.00		1.00		1.00	
Female	0.32	(0.29–0.35)	0.35	(0.30–0.42)	0.41	(0.33–0.51)	0.35	(0.27–0.43)	0.74	(0.64–0.87)	1.17	(1.09–1.24)	0.69	(0.62–0.77)	1.15	(1.07–1.24)	1.01	(0.93–1.09)
Educational attainment																		
Less than HS	1.70	(1.27–2.27)	1.43	(0.89–2.30)	1.16	(0.61–2.22)	1.66	(0.91–3.01)	0.88	(0.50–1.55)	1.27	(1.02–1.57)	1.71	(1.26–2.33)	1.60	(1.28–2.01)	1.54	(1.20–1.98)
HS graduate	1.14	(1.03–1.26)	1.08	(0.92–1.27)	1.07	(0.87–1.31)	1.02	(0.82–1.27)	0.86	(0.73–1.00)	1.12	(1.05–1.19)	1.11	(0.99–1.24)	1.03	(0.96–1.12)	0.98	(0.90–1.07)
Post-secondary and graduate [reference]	1.00		1.00		1.00		1.00		1.00		1.00		1.00		1.00		1.00	
FPCEs																		
0–1 FPCEs [reference]	1.00		1.00		1.00		1.00		1.00		1.00		1.00		1.00		1.00	
2 FPCEs	0.99	(0.86–1.15)	0.99	(0.80–1.24)	0.77	(0.58–1.01)	0.76	(0.57–1.01)	0.86	(0.69–1.06)	1.00	(0.91–1.10)	0.79	(0.67–0.93)	0.75	(0.67–0.83)	0.68	(0.60–0.77)
3 FPCEs	0.83	(0.72–0.96)	0.68	(0.54–0.87)	0.49	(0.36–0.68)	0.36	(0.25–0.52)	0.67	(0.53–0.83)	0.94	(0.86–1.03)	0.61	(0.50–0.73)	0.55	(0.49–0.61)	0.53	(0.46–0.60)

AOR, adjusted odds ratio ACEs, adverse childhood experiences; CPCEs, community positive childhood; HS, high school; FPCEs, family positive childhood experiences

For peer review only

For peer review only - http://bmjopen.bmj.com/site/about/guidelines.xhtml

Table S7 Marginal effects with test of interaction effects

	**Marginal effects (95% CI)**		**Differences of two marginal effects (95% CI)**	P value*
	0–2 CPCEs	≥3 CPCEs		
Diabetes	0.018 (0.011 to 0.025)	0.010 (−0.003 to 0.022)	0.008 (−0.023 to 0.006)	0.248
Ischemic heart disease	0.013 (0.009 to 0.018)	−0.001 (−0.008 to 0.006)	0.015 (0.006 to 0.023)	0.001
Stroke	0.012 (0.008 to 0.016)	0.003 (−0.002 to 0.009)	0.009 (0.006 to 0.023)	0.011
COPD	0.012 (0.008 to 0.016)	−0.002 (−0.006 to 0.003)	0.014 (0.008 to 0.020)	0.000
Cancer	0.015 (0.010 to 0.020)	0.001 (−0.006 to 0.009)	0.013 (0.005 to 0.022)	0.002
Chronic pain	0.088 (0.077 to 0.099)	0.049 (0.030 to 0.068)	0.039 (0.017 to 0.061)	0.000
Depression	0.055 (0.049 to 0.062)	0.023 (0.012 to 0.033)	0.033 (0.020 to 0.046)	0.000
Suicidal ideation	0.098 (0.088 to 0.108)	0.041 (0.025 to 0.057)	0.057 (0.038 to 0.076)	0.000
Severe psychological distress	0.084 (0.075 to 0.092)	0.033 (0.019 to 0.046)	0.051 (0.035 to 0.067)	0.000

*P value for significant differences by test of the equality of marginal effects (Stata: margins, contrast).

For peer review only

STROBE Statement—Checklist of items that should be included in reports of ***cross-sectional studies***

	Item No	Recommendation	Page No
**Title and abstract**	1	(*a*) Indicate the study's design with a commonly used term in the title or the abstract	1-2
		(*b*) Provide in the abstract an informative and balanced summary of what was done and what was found	2
**Introduction**			
Background/rationale	2	Explain the scientific background and rationale for the investigation being reported	4
Objectives	3	State specific objectives, including any prespecified hypotheses	5
**Methods**			
Study design	4	Present key elements of study design early in the paper	4-5
Setting	5	Describe the setting, locations, and relevant dates, including periods of recruitment, exposure, follow-up, and data collection	6
Participants	6	(*a*) Give the eligibility criteria, and the sources and methods of selection of participants	6
Variables	7	Clearly define all outcomes, exposures, predictors, potential confounders, and effect modifiers. Give diagnostic criteria, if applicable	7-9
Data sources/ measurement	8*	For each variable of interest, give sources of data and details of methods of assessment (measurement). Describe comparability of assessment methods if there is more than one group	*7-9*
Bias	9	Describe any efforts to address potential sources of bias	6
Study size	10	Explain how the study size was arrived at	6
Quantitative variables	11	Explain how quantitative variables were handled in the analyses. If applicable, describe which groupings were chosen and why	7-9
Statistical methods	12	(*a*) Describe all statistical methods, including those used to control for confounding	9
		(*b*) Describe any methods used to examine subgroups and interactions	9
		(*c*) Explain how missing data were addressed	6
		(*d*) If applicable, describe analytical methods taking account of sampling strategy	9
		(*e*) Describe any sensitivity analyses	9
**Results**			
Participants	13*	(a) Report numbers of individuals at each stage of study—eg numbers potentially eligible, examined for eligibility, confirmed eligible, included in the study, completing follow-up, and analysed	6
		(b) Give reasons for non-participation at each stage	6
		(c) Consider use of a flow diagram	6
Descriptive data	14*	(a) Give characteristics of study participants (eg demographic, clinical, social) and information on exposures and potential confounders	9-11
		(b) Indicate number of participants with missing data for each variable of interest	9-11
Outcome data	15*	Report numbers of outcome events or summary measures	10
Main results	16	(*a*) Give unadjusted estimates and, if applicable, confounder-adjusted estimates and their precision (eg, 95% confidence interval). Make clear which confounders were adjusted for and why they were included	11-13

For peer review only - http://bmjopen.bmj.com/site/about/guidelines.xhtml

		(*b*) Report category boundaries when continuous variables were categorized	10-12
		(*c*) If relevant, consider translating estimates of relative risk into absolute risk for a meaningful time period	11-13
Other analyses	17	Report other analyses done—eg analyses of subgroups and interactions, and sensitivity analyses	12-13
**Discussion**			
Key results	18	Summarise key results with reference to study objectives	13
Limitations	19	Discuss limitations of the study, taking into account sources of potential bias or imprecision. Discuss both direction and magnitude of any potential bias	15
Interpretation	20	Give a cautious overall interpretation of results considering objectives, limitations, multiplicity of analyses, results from similar studies, and other relevant evidence	13-15
Generalisability	21	Discuss the generalisability (external validity) of the study results	13-15
**Other information**			
Funding	22	Give the source of funding and the role of the funders for the present study and, if applicable, for the original study on which the present article is based	17

*Give information separately for exposed and unexposed groups.

Note: An Explanation and Elaboration article discusses each checklist item and gives methodological background and published examples of transparent reporting. The STROBE checklist is best used in conjunction with this article (freely available on the Web sites of PLoS Medicine at http://www.plosmedicine.org/, Annals of Internal Medicine at http://www.annals.org/, and Epidemiology at http://www.epidem.com/). Information on the STROBE Initiative is available at www.strobe-statement.org.

For peer review only

For peer review only - http://bmjopen.bmj.com/site/about/guidelines.xhtml

BMJ Open

Promotive and protective effects of community-related positive childhood experiences on adult health outcomes in the context of adverse childhood experiences: a nationwide cross-sectional survey in Japan

Journal:	*BMJ Open*
Manuscript ID	bmjopen-2023-082134.R3
Article Type:	Original research
Date Submitted by the Author:	24-Apr-2024
Complete List of Authors:	Mitani, Haruyo; Osaka University, Graduate School of Human Sciences<br>Kondo, Naoki; Kyoto University, Graduate School of Medicine and School of Public Health, Department of Social Epidemiology<br>Amemiya, Airi; Kyoto University, Graduate School of Medicine and School of Public Health, Department of Social Epidemiology (former affiliation)<br>Tabuchi, Takahiro; Osaka International Cancer Institute, Cancer Control Center; Tohoku University, Graduate School of Medicine, School of Public Health, Division of Epidemiology
**Primary Subject Heading**:	Epidemiology
Secondary Subject Heading:	Communication, Epidemiology, Mental health, Sociology
Keywords:	Chronic Disease, EPIDEMIOLOGY, MENTAL HEALTH, Social Interaction, Stress, Psychological

SCHOLARONE™
Manuscripts

I, the Submitting Author has the right to grant and does grant on behalf of all authors of the Work (as defined in the below author licence), an exclusive licence and/or a non-exclusive licence for contributions from authors who are: i) UK Crown employees; ii) where BMJ has agreed a CC-BY licence shall apply, and/or iii) in accordance with the terms applicable for US Federal Government officers or employees acting as part of their official duties; on a worldwide, perpetual, irrevocable, royalty-free basis to BMJ Publishing Group Ltd ("BMJ") its licensees and where the relevant Journal is co-owned by BMJ to the co-owners of the Journal, to publish the Work in this journal and any other BMJ products and to exploit all rights, as set out in our licence.

The Submitting Author accepts and understands that any supply made under these terms is made by BMJ to the Submitting Author unless you are acting as an employee on behalf of your employer or a postgraduate student of an affiliated institution which is paying any applicable article publishing charge ("APC") for Open Access articles. Where the Submitting Author wishes to make the Work available on an Open Access basis (and intends to pay the relevant APC), the terms of reuse of such Open Access shall be governed by a Creative Commons licence – details of these licences and which Creative Commons licence will apply to this Work are set out in our licence referred to above.

Other than as permitted in any relevant BMJ Author's Self Archiving Policies, I confirm this Work has not been accepted for publication elsewhere, is not being considered for publication elsewhere and does not duplicate material already published. I confirm all authors consent to publication of this Work and authorise the granting of this licence.

For peer review only

Title

Promotive and protective effects of community-related positive childhood experiences on adult health outcomes in the context of adverse childhood experiences: a nationwide cross-sectional survey in Japan

Authors

Haruyo Mitani[1], Naoki Kondo[2], Airi Amemiya, Takahiro Tabuchi[3,4]

Author Affiliations

[1]Graduate School of Human Sciences, Osaka University, Osaka, Japan

[2]Department of Social Epidemiology, Graduate School of Medicine and School of Public Health, Kyoto University, Kyoto, Japan

[3]Cancer Control Center, Osaka International Cancer Institute, Osaka, Japan

[4]Division of Epidemiology, School of Public Health, Graduate School of Medicine, Tohoku University, Sendai, Japan

Correspondence to

Haruyo Mitani, Graduate School of Human Sciences, Osaka University, Osaka, Japan

mitani.haruyo.hus@osaka-u.ac.jp

For peer review only - http://bmjopen.bmj.com/site/about/guidelines.xhtml

ABSTRACT

Objective: Although adverse childhood experiences (ACEs) are associated with poor health in adulthood, positive childhood experiences (PCEs) can reduce the risk of negative health outcomes. This study aimed to investigate whether PCEs in the community (CPCEs, i.e., trusted adults other than parents, supportive friends, belongingness to school, or community traditions) would have an independent effect on better health outcomes and moderate the association between ACEs and adult illnesses.

Design: Cross-sectional survey

Setting: Data were gathered from a nationwide, cross-sectional Internet survey conducted in Japan in 2022.

Participants: This study included 28,617 Japanese adults aged 18–82 years (51.1% female; mean age = 48.1 years).

Primary and secondary outcome measures: The associations among self-reported ACEs, CPCEs before the age of 18 years, and current chronic diseases (e.g., cancer and depression) were investigated using multivariable logistic regression models.

Results: CPCEs were associated with lower odds of adult diseases (such as stroke, chronic obstructive pulmonary disease [COPD], chronic pain, depression, suicidal ideation, and severe psychological distress) after adjusting for ACEs. More CPCEs weakened the association between ACEs and adult diseases. Specifically, among those with ACEs, ≥3 CPCEs (vs. 0–2 CPCEs) lowered the adjusted prevalence by ≥50% for stroke (2.4→1.2%), COPD (2.2→0.7%), and severe psychological distress (16.4→7.4%).

Conclusion: CPCEs could reduce ACE-related risk of poor physical and mental health in later

For peer review only - http://bmjopen.bmj.com/site/about/guidelines.xhtml

life. Early-life interventions that enhance PCEs in schools and/or neighborhoods are recommended.

Strengths and limitations of this study

- This study used a large and representative sample from a nationwide survey in Japan.
- Multivariable logistic regression models were used to adjust for possible confounders and to evaluate the associations among ACEs, CPCEs, and adult diseases.
- Due to the self-reported and retrospective nature of the survey data, the possibility of recall bias cannot be eliminated.
- A cross-sectional design limited our ability to determine causality.
- The CPCEs measure used in this study did not include all CPCEs.

For peer review only

INTRODUCTION

Over the last quarter-century, a growing body of research has demonstrated the harmful effects of adverse childhood experiences (ACEs), such as maltreatment and exposure to household dysfunction, on long-term physical and mental health[1–6]. Studies have emphasized a dose-response relationship between an individual's ACE score and their risk of having poor health; that is, the greater the exposure to ACEs, the greater the risk of physical and mental illness in later life. Chronic childhood stress exposure can lead to dysregulation of stress response systems, including the hypothalamic-pituitary-adrenocortical axis and the sympathetic-adrenomedullary system, resulting in permanent changes in multiple organ systems, particularly the brain[7]. Epigenetic modifications of DNA could play a role in these changes, and the biological disruptions of brain circuits, other organs, and metabolic systems during sensitive development are thought to lead to an increased risk of various chronic diseases in adulthood[8].

Meanwhile, recent evidence shows a potential role of positive childhood experiences (PCEs). For example, continual early assistance from a reliable adult[9], an adult who felt safe and protected as a child[10], or PCEs inside and outside the home (cumulative scale consisting of seven items such as "able to talk to family about feelings" and "felt supported by friends")[11], moderates the association between ACEs and negative consequences, represented by poor mental health. In other words, PCEs have a moderating effect as a protective factor; that is, the harmful effects of ACEs can be smaller among individuals who report more PCEs than among those who report fewer PCEs [9–15].

Resilience refers to the latent or apparent capacity to successfully respond to situations that threaten an individual's functioning, survival, or development[12]. Based on the Resilience Theory[12], the socio-ecological system suggests that multiple systems (e.g., individuals,

For peer review only - http://bmjopen.bmj.com/site/about/guidelines.xhtml

families, and communities) complementarily influence youth development. Moreover, factors promoting resilient outcomes can be divided into individual (e.g., cognition, coping style, and self-esteem), family (e.g., a safe and supportive relationship with a caregiver), and community factors (e.g., positive relationships with friends and neighbors)[12–15]. Notably, the concept of PCEs includes both family and community factors. Little is understood about the potential of PCEs in the community (CPCEs) for countering the negative lifetime effects of ACEs.

The present study focused on the role of CPCEs (PCEs in the community outside the home). Compared to those with fewer ACEs, children exposed to a higher number of ACEs are less likely to report having adults in their lives who made them feel safe or met their basic needs, highlighting a deficit in positive childhood experiences (PCEs)[10]. In other words, those with more ACEs are less likely to have experienced PCEs in the home. Hence, continuous family intervention by professionals to change how caregivers relate to children is desirable to enrich family PCEs (FPCEs)[16]. However, many countries lack the infrastructure (staff, funds, and legal structure) to make such professional intervention possible, and Japan is no exception[17]. However, community interventions in schools and neighborhoods can easily and inexpensively positively impact the development of children exposed to ACEs. Clarifying the role of CPCEs is essential for understanding the effectiveness of community interventions.

Therefore, this study explored the effect of CPCEs on the negative consequences of ACEs, focusing on four items of the PCE scale[11], referenced in many studies, that are considered particularly present in the community (i.e., trusted adults other than parents, supportive friends, belongingness to school, and community traditions). Similar to the original ACE study[1], this study used a broad range of physical and mental illnesses as primary outcomes. In Japan, while ACEs influence physical illnesses in old age[18] and mental disorders in adults[19], the interplay between PCEs and ACEs on health remains unexplored. Stress is identified as a

For peer review only - http://bmjopen.bmj.com/site/about/guidelines.xhtml

contributing factor to adult diseases[20]. ACEs are considered stressors originating within the childhood home, whereas CPCEs are viewed as external factors that mitigate the impact of these stressors.

This study explored the associations among ACEs, CPCEs, and adult diseases. First, the relationship between ACEs and adult diseases was ascertained. Then, the Resilience Theory's three main models (the Compensatory Model, the Protective Factors Model, and the Challenge Model [12,21–23]) were examined.

The Compensatory (Promotive Factors) Model assumes that promotive/compensatory factors directly impact developmental outcomes independent of risk factors. For example, even after controlling for ACEs, PCEs have been reported to predict less post-traumatic syndrome disorder symptoms and stress in pregnant women[24] and less depression, stress, and sleep difficulties in adults[25]. While ACEs could still have a significant effect on the outcome, CPCEs themselves are thought to enhance health in adulthood.

The Protective Factors Model assumes that protective factors mitigate the relationship between risk factors and bad outcomes. Protective factors do not act independently but can interact with risk factors. Previous studies have shown that adults who protect or meet basic needs[10] and high PCE scores[11] were associated with less poor mental health, especially among those with ACEs. It has also been found that in those with ≥4 ACEs, having all childhood community resilience assets such as given opportunity, supportive friends, and role model (vs. none) lowered the prevalence of self-rated poor childhood health from 59.8% to 21.3%[26]. In situations where more CPCEs are reported, the negative impact of ACEs on adult health is likely to be smaller.

The Challenge Model assumes that the modest levels of risk factors protect against additional

For peer review only - http://bmjopen.bmj.com/site/about/guidelines.xhtml

exposures that could render people prone to unfavorable consequences. Moderate levels of ACEs, particularly when accompanied by more protective factors, may promote improved adult health, akin to inoculation[22]. Some studies did not support this model[21], but it has also been reported that PCE score had greater positive effects on adult health (such as body mass index [BMI] and sleep difficulties) among those with <4 ACEs than among those with ≥4 ACEs[25]. Therefore, it can be speculated whether moderate ACEs can lower the likelihood of adult illness among individuals reporting more CPCEs.

Thus, this study tested three hypotheses: CPCEs directly affect adult health (H1), the association between ACEs and adult diseases is weaker among people with more CPCEs (H2), and ACEs are negatively associated with adult disease among people with more CPCEs (H3).

METHODS

Sample

The Japan COVID-19 and Society Internet Survey (JACSIS)[27] was conducted in 2022 by a major Internet research agency (Rakuten Insight, Inc.) with around 2.2 million registered qualified panelists. Of the panelists, 224,389 males and females aged 15–79 were invited to participate in the survey based on a random sample stratified by sex, age, and all 47 prefectures. Participants were rewarded with "E-Points" for online shopping. Informed consent was obtained from all participants before the survey. The survey began on September 12, 2022, and ended on October 19, 2022. The survey was terminated after the target of 32,000 participants was reached.

This study was authorized by the Research Ethics Committee of the Osaka International

For peer review only - http://bmjopen.bmj.com/site/about/guidelines.xhtml

Cancer Institute (approved on June 14, 2022; approval No. 20084-8) and followed the Helsinki guidelines.

Participants who provided contradictory answers to the screening questions were excluded from the study. These included the following criteria: (i) invalid answers to the screening question (participants were asked to choose the second-last option, but they chose other options), (ii) positive responses to all items on drug use, (iii) positive answers for having all 20 chronic diseases, and (iv) a total household size of over 15 individuals. There were 3,370 respondents who gave inconsistent answers, leaving 28,630 individuals. Thirteen participants aged <18 were also excluded since ACEs and PCEs are answered by recalling experiences up to age 18. Finally, 28,617 adults were included. There were no significant differences in the key variables (ACEs, PCEs, and adult health outcomes) between 28,617 adults included in the analysis and 3,370 adults not included in the study. Due to the nature of the online survey, there were no missing values.

Patient and public involvement

Neither patients nor the public were involved in this study.

Measurements

ACEs

Questions on ACEs were derived from the ACEs scale for the Japanese context[28]. Although this study was based on the original ACE study[1], ACE items included parental death, except the incarceration of household members, to fit the Japanese context. Respondents were

For peer review only - http://bmjopen.bmj.com/site/about/guidelines.xhtml

questioned whether any of the following adversities had occurred in their households by age 18: (i) death of either parent; (ii) parental divorce or separation; (iii) mental illness of either parent; (iv) substance abuse in the household (e.g., parental addiction to alcohol and gambling); (v) mother treated violently (the father was violent with the mother); (vi) emotional abuse (my parent said hurtful things or insulted me); (vii) physical abuse (my parent beat and hurt me); (viii) sexual abuse (an adult sexually touched me); (ix) emotional neglect (I always felt suffocated because my parent did not respect my opinion); and (x) physical neglect (I did not receive necessary care, such as eating and dressing). The response categories "yes" and "no" were coded as dummy variables, and these 10 items were summed into ACE scores (0–10). This score was classified into two categories (0 vs. ≥1) because of their effectiveness in determining poor health, following those of previous studies[18,29] and the clarity of the semantic difference between "without ACEs" and "with ACEs."

PCEs

PCEs were assessed using seven items adapted from the PCE scale[11]. Participants answered whether they had experienced any of the following by the time they were 18 years old: (i) felt able to talk to their family about feelings; (ii) felt their family stood by them during difficult times; (iii) felt safe and protected by an adult in their home; (iv) had at least two non-parental adults who took genuine interest in them; (v) felt supported by friends; (vi) felt a sense of belonging in middle and high school; and (vii) enjoyed participating in community traditions. Although these questions were asked in Japanese, and some were modified from the original questions (the addition of "middle" in item 6), these seven items can be considered almost identical to the PCEs items used by Bethell et al.[11] Of these items, the first three were positioned as components of FPCEs and the others as CPCEs (Supplementary Table S1). The

For peer review only - http://bmjopen.bmj.com/site/about/guidelines.xhtml

applicable items were summed to obtain the FPCE (0–3) and CPCE (0–4) scores. As the PCE scale[11], principal component analyses revealed a single-component structure for each FPCE and CPCE scale. Cronbach's alpha was 0.81 and 0.75 for FPCEs and CPCEs, respectively (Supplementary Table S2).

Adult health outcomes

Participants answered whether they currently had the following diseases using a dichotomous response variable (yes/no): (i) diabetes, (ii) ischemic heart disease (angina pectoris or myocardial infarction), (iii) stroke (cerebral infarction or hemorrhage), (iv) chronic obstructive pulmonary disease (COPD), (v) cancer, (vi) chronic pain (e.g., back pain and headache lasting ≥3 months), and (vii) depression. Additionally, (viii) suicidal ideation was determined as a "yes" response to the question, "Have you had times when you have wanted to die in the last year?" Psychological distress was measured with the Japanese version[30] of the Kessler 6-item psychological distress scale (K6)[31], assessing depressed mood and anxiety. The total score ranged from 0 to 24 on a five-point scale (0–4). A score of ≥13 was considered indicative of (ix) serious psychological distress[32].

Covariates

The following background variables reported by participants were included as covariates, in line with previous studies[1,33,34]: age (continuous by year), sex (0 "male" or 1 "female"), and educational attainment (three dummy variables for "less than high school," "high school," and "post-secondary and graduate").

For peer review only

Statistical analysis

Demographic characteristics and prevalence rates of all health outcomes in the sample were examined. For H1, multivariable logistic regression analyses were conducted to consider the direct effect of CPCEs on adult diseases after adjusting for covariates, ACEs, and FPCEs. For H2 and H3, multiple logistic regression analyses, stratified by CPCE exposure levels, were performed to examine whether CPCEs moderated the link between ACEs and adult diseases, adjusting for covariates and FPCEs. Interactive effects and marginal predicted probabilities were estimated to assess the statistical significance of the moderating effects of CPCEs on the association between ACEs and adult diseases[35]. The extent to which CPCEs reduced the risk of each disease was also estimated.

The survey data were weighted to represent the Japanese population. Considering the possibility of multicollinearity, the variance inflation factor (VIF) for each explanatory variable was checked to ensure that it did not exceed 4.0[36]. Stata 17.0 MP (StataCorp LLC, Texas, USA) was used for all analyses.

RESULTS

Participant characteristics

Table 1 shows that chronic pain and COPD were the most prevalent (18.6%) and least prevalent (1.4%) of the nine health outcomes, respectively. Approximately 39.8% of the participants had experienced ACEs. Furthermore, 52.8% and 56.0% of participants had experienced one or more FPCEs and CPCEs, respectively. When ACEs were divided into four exposure

For peer review only

For peer review only - http://bmjopen.bmj.com/site/about/guidelines.xhtml

levels[9,11], the more exposure to ACEs, the higher the prevalence of adult diseases, the lower the exposure rates to the two types of PCEs, and the higher the proportion of females and those with lower education levels (Supplementary Table S3). Finally, dose-response relationships between ACE exposure levels and all adult diseases were observed (Supplementary Table S4).

Table 1 Demographic characteristics and prevalence of health outcomes, ACEs, and PCEs among participants (N = 28617)

	N	W %/M (SD)
Health outcomes		
Diabetes	1945	7.7
Ischemic heart disease	686	3.0
Stroke	433	1.7
COPD	381	1.4
Cancer	705	2.6
Chronic pain	5062	18.6
Depression	1381	4.7
Suicidal ideation	3909	14.4
Severe psychological distress	2881	10.4
ACEs (0–11)	28617	0.81 (1.4)
0 ACEs	17889	60.2
≥1 ACEs	10728	39.8
FPCEs (0–3)	28617	1.14 (1.2)
0 FPCE	12003	47.2
1 FPCEs	4133	14.0
2 FPCEs	5025	16.2
3 FPCEs	7456	22.7
CPCEs (0–4)	28617	1.25 (1.4)
0 CPCE	11051	44.0
1 CPCEs	5543	19.4
2 CPCEs	4502	14.7
3 CPCEs	3922	11.5
4 CPCEs	3599	10.5
Age (18–82 years)	28617	48.1 (17.2)

For peer review only - http://bmjopen.bmj.com/site/about/guidelines.xhtml

Sex		
Male	13993	48.9
Female	14624	51.1
Educational attainment		
Less than HS	493	4.4
HS graduate	10516	60.3
Post-secondary and graduate	17397	33.8

W, weighted; SD, standard deviation; ACEs, adverse childhood experiences; CPCEs, community positive childhood experiences; FPCEs, family positive childhood experiences; HS, high school

For peer review only

Health outcomes according to ACE, FPCE, and CPCE exposure

Table 2 shows the adjusted odds ratios (AORs) for the poor health associated with CPCEs. In all models, VIF values did not exceed the criterion value (4.0). CPCEs had significant negative direct effects on six adult diseases: stroke, COPD, chronic pain, depression, suicidal ideation, and severe psychological distress, after adjusting for covariates, ACEs, and FPCEs. This result supported H1, which states that CPCEs directly affect adult health, derived from the Compensatory Model. For example, the adjusted odds of stroke were 15% lower for each additional CPCE. The same trend was found when CPCEs were treated as categorical variables (Supplementary Table S5). Overall, CPCEs were particularly associated with lower odds of mental illness.

Table 2 Adjusted odds ratios of health outcomes by ACEs (≥1 vs. 0), FPCEs, and CPCEs

	ACEs		FPCEs		CPCEs	
	**AOR***	**(95% CI)**	**AOR†**	**(95% CI)**	**AOR‡**	**(95% CI)**
Diabetes	**1.31**	(1.18–1.44)	**0.93**	(0.89–0.99)	0.97	(0.93–1.02)
Ischemic heart disease	**1.53**	(1.31–1.80)	**0.89**	(0.82–0.97)	0.93	(0.87–1.01)
Stroke	**1.96**	(1.61–2.39)	**0.83**	(0.75–0.93)	**0.85**	(0.77–0.93)

For peer review only - http://bmjopen.bmj.com/site/about/guidelines.xhtml

COPD	**2.00**	(1.62–2.47)	**0.77**	(0.69–0.86)	**0.89**	(0.81–0.98)
Cancer	**1.62**	(1.39–1.90)	**0.89**	(0.82–0.97)	0.95	(0.89–1.02)
Chronic pain	**1.71**	(1.60–1.82)	0.98	(0.95–1.02)	**0.91**	(0.89–0.94)
Depression	**2.86**	(2.55–3.22)	**0.89**	(0.83–0.95)	**0.81**	(0.76–0.86)
Suicidal ideation	**2.12**	(1.97–2.28)	**0.84**	(0.81–0.88)	**0.77**	(0.74–0.80)
Severe psychological distress	**2.29**	(2.10–2.48)	**0.86**	(0.81–0.90)	**0.72**	(0.69–0.75)

N = 28617; Significance level of data in bold font <0.05.

*Adjusted for age, sex, educational attainment, FPCEs, and CPCEs.

†Adjusted for age, sex, educational attainment, ACEs, and CPCEs.

‡Adjusted for age, sex, educational attainment, ACEs, and FPCEs.

ACEs, adverse childhood experiences; FPCEs, family positive childhood experiences; CPCEs, community positive childhood experiences; AOR, adjusted odds ratio; CI, confidence interval.

For peer review only

Health outcomes of ACEs across CPCE exposure levels

As shown in Figure 1, the AORs declined as PCE exposure levels rose; the cells become greener to the right. Particularly, the AORs tended to be smaller when the CPCE scores were ≥3 than when they were 0–2. This value of 3 CPCEs is also consistent with the Aiken and West[37] method, which recommends that the cutoff value is close to the mean plus one standard deviation (mean1.25+sd1.4 = 2.65). Therefore, the interaction effects of ACEs and CPCEs (≥3 vs. 0–2) on all nine outcomes were examined (Supplementary Table S6). Although the rightmost column of Figure 1 shows that the regression coefficients on the interaction terms between any ACE and ≥3 CPCEs were significant at the 10% level for seven diseases, it is a better way to examine the predicted probability themselves and perform the test of the equality of marginal effects to determine whether significant interaction effects exist[35].

Figure 2 shows the predicted values for each disease obtained from the regression equations. The results of the marginal effect difference test (the red values of Figure 2 and Supplementary Table S7) showed significant differences in the marginal effects (the amount of change in

For peer review only - http://bmjopen.bmj.com/site/about/guidelines.xhtml

predicted probability when ACEs exceed ≥1 from 0) between those with 0–2 CPCEs and those with ≥3 CPCEs for eight diseases except diabetes. CPCEs moderated the association between ACEs and eight adult diseases. This result supported H2, which states that the association between ACEs and adult diseases is weaker among people with more CPCEs, derived from the Protective Factors Model. Specifically, in ischemic heart disease, the difference between the marginal effect of 0–2 CPCEs (0.034−0.21 = 0.013) and one of ≥3 CPCEs (0.017−0.019 = −0.001) was 0.015, significant at the 1% level. According to each marginal effect (Supplementary Table S7), the marginal effect for CPCEs ≥3 was negative for ischemic heart disease (−0.001, 95% CI −0.008 to 0.006) and COPD (−0. 002, 95% CI −0.006 to 0.003). A negative association between ACEs and adult disease in the context of reporting more CPCEs was suggested, but H3 was not statistically supported, as suggested by the Challenge Model.

From Figure 2, the differences in predicted morbidity by CPCEs among those with ACEs can also be captured as relative risk reduction. For example, the predicted prevalence of ischemic heart disease did not differ according to CPCE exposure among those without ACEs; however, among those with ACEs, it differed from 3.4% in those with 0–2 CPCEs to 1.7% in those with ≥3 CPCEs (relative risk: 1.725/3.400*100 = 50.794%, relative risk reduction: 100.0 −50.8 = 49.2%). The adjusted prevalence was reduced by almost ≥50% for ischemic heart disease (49.2%), stroke (51.9%), COPD (67.1%), depression (49.1%), and severe psychological distress (54.9%).

DISCUSSION

This study examined the associations among ACEs, CPCEs, and adult diseases using a population-based sample from Japan. Specifically, ACEs were associated with increased odds

For peer review only - http://bmjopen.bmj.com/site/about/guidelines.xhtml

of adult diseases, while CPCEs directly affected adult illnesses as a promotive factor and mitigated the adverse health effects of ACEs as a protective factor. The protective effect of CPCEs was particularly pronounced in stroke, COPD, and severe psychological distress, with associated relative risk reductions of 50% or more by three or more CPCEs in those with ACEs.

Consistent with earlier research[1,4,6], the results showed a dose-response connection between ACEs and all adult diseases evaluated. Although an association between ACEs and diseases during old age among Japanese individuals was previously reported[18], this appeared robust across a wide age range (18–82 years). The ACE items are based on the original ACE study[1]. Although this measure does not include the variety of traumatic experiences that participants may have, it is noteworthy that a dose-response relationship with adult health was found, as in many studies.

H1, based on the Compensatory Model, was supported for many of the diseases assessed in this study. CPCEs, independent of ACEs and FPCEs, were directly associated with lower odds of adult-onset illnesses (i.e., stroke, COPD, chronic pain, depression, suicidal ideation, and severe psychological distress). While previous studies have found direct effects of PCEs primarily on mental health[11,25,34], the present study adds a novel finding that CPCEs independently function as promotive factors for physical and mental health.

More importantly, this study found analytical support for H2 based on the Protective Model. CPCEs moderated the relationships between ACEs and adult diseases (i.e., ischemic heart disease, stroke, COPD, cancer, chronic pain, depression, suicidal ideation, and severe psychological distress), even after adjusting for FPCEs. Although the possible protective function of PCEs has been noted[9–11], the buffering effect may be due to family factors. Notably, the present study observed that CPCEs can independently play a protective role. Furthermore, extending research focusing on community assets moderating the association

For peer review only - http://bmjopen.bmj.com/site/about/guidelines.xhtml

between ACEs and poor child health[26], this study suggests that the moderating effects of CPCEs may spread to adulthood, indicating that the protective effect may be stronger in cumulative situations where three or more CPCEs are aligned. The associated relative risk reductions of physical illness (i.e., ischemic heart disease, stroke, and COPD) and mental illness (i.e., depression and severe psychological distress) in ACE survivors by almost more than half due to ≥3 CPCEs would be a non-negligible value. Whether this cutoff point is appropriate requires follow-up testing; however, the harm caused by ACEs may be mitigated in multiple relationships.

Moreover, the analysis yielded the finding that, for several diseases (i.e., ischemic heart disease and COPD), those with ACEs had lower prevalence rates than those without ACEs in situations reporting more CPCEs (Figure 2). Although the negative marginal effects of ACEs on these illnesses were not statistically significant, as the Challenge Model[22] of the Resilience Theory implies, moderate ACEs promote health when sufficient protective factors exist; that is, they act as immunization. Future studies should examine the generalizability of this phenomenon.

Why might CPCEs contribute to developing children and youth into healthier adults? Prior research argues that supportive adults other than parents may provide opportunities for children to elude life stressors, establish positive experiences in social connections, and feel a sense of having a place and social association[38]. Additionally, supportive close friendships promote resilience by developing constructive coping styles, encouraging efforts, providing supportive peer networks, and reducing inappropriate coping[39]. In interactions with non-family members at school and in the neighborhood, children exposed to ACEs may gain physical refuge from toxic stress and develop psychological and instrumental coping strategies. The role of CPCEs as a promoting and protective factor found in this study may have been discovered

For peer review only - http://bmjopen.bmj.com/site/about/guidelines.xhtml

only in the context of Japan, where many local communities have self-governing organizations and traditional events, and violence among residents and students is relatively rare. Future studies should examine whether the findings are generalizable to other countries besides Japan.

Strengths and limitations

To the best of our knowledge, this is the first study to suggest that CPCEs could be promotive and protective factors of health system resilience. A strength of this study is the use of a large and representative sample. Moreover, various indicators elucidated the complex relationships among ACEs, PCEs, and adult diseases.

However, this study had some limitations. First, the data were self-reported and retrospective, which could have been influenced by recall bias. Individuals with a disease may be more likely to recall ACEs than healthy individuals. However, asking participants to reflect on their growing environment from a balanced perspective by asking about PCEs may have reduced this recall bias. Second, because of the study's cross-sectional nature, the possibility of reverse causation between ACEs/PCEs and adult illnesses cannot be eliminated. This study could not allow for the determination of causality. Third, the CPCEs measure used in this study did not include all CPCEs. Other types of CPCEs, such as participation in non-traditional activities and self-realization experiences, should be included in developing the CPCEs scale. Fourth, confounding variables (including psychological traits like optimism and hometown regional features, which may be linked to adult diseases, ACEs, and CPCEs) might have gone unnoticed. Fifth, the extent of the positive effects of the CPCEs differed for each health outcome. Particularly, the role of CPCEs in mental illness was found to be robust; however, the validity of these effects should be carefully investigated by including other health outcomes. Sixth, this

For peer review only - http://bmjopen.bmj.com/site/about/guidelines.xhtml

study could not address a variety of traumatic experiences (e.g., homelessness, bullying victimization, and childhood illness) other than typical ACEs.

Policy, practice, and research implications

The findings suggest that community-based interventions can be effective in helping children who have experienced adversity become resilient adults. Public health, social welfare, and educational practices that enforce CPCEs might as well be promoted. Additionally, the content of specific community-level approaches depends on social and local contexts. Such interventions are desirable to incorporate the main components of the Healthy Outcomes from Positive Experiences framework: (i) supportive and nurturing relationships, (ii) safe and stable environments, (iii) opportunities for constructive social involvement and connection, and (iv) social and emotional capacities[40]. For example, The National Resource Center at Tufts Medical Center[41] supports the above framework, providing tools and training to help healthcare providers, social service providers, educators, and members of community-based organizations acquire essential knowledge and skills to integrate these elements into their work.

In Japan, school counselors[42] and school social workers[43] in public elementary and junior high schools are expected to become resilience resources under compulsory education. Additionally, teachers might be a good way to get training in PCEs and improved working environments to build trusting relationships with their students. In local communities, traditional events such as festivals, nature experiences, multigenerational exchange programs, and the recently popularized "*Kodomo Shokudo*" (cafeterias for children)[44] could also contribute to the children's resilience. Further research and practice are expected to explore effective approaches to promoting CPCEs, including historical and new engagements.

For peer review only - http://bmjopen.bmj.com/site/about/guidelines.xhtml

Conclusion

This study showed that CPCEs positively impacted adult health in the general population regardless of ACE exposure and mitigated the adverse consequences. The study demonstrates that CPCEs may contribute to the lifelong health status of ACE survivors. Therefore, further research is needed to investigate the impact mechanisms of CPCEs and improve the scale's accuracy while capturing diverse childhood adversities, as well as interventions to strengthen supportive communities.

Contributors: HM and AA conceptualized the study. HM conducted the analysis and drafted the first manuscript. NK and AA contributed intellectual content to the study design and interpretation of the findings. TT collected data, managed the project, and obtained funding. All authors critically reviewed the manuscript and approved the final manuscript. HM is the guarantor for this study.

Funding: This study (JACSIS2022) was supported by the Japan Society for the Promotion of Science (JSPS) KAKENHI Grants (21H04856, 20K10467, 20K19633, 20K13721), the JST Grant (JPMJPF2017), the Health Labor Sciences Research Grant (21HA2016), the grant for 2021-2022 Strategic Research Promotion (SK202116) of Yokohama City University, and the research program on "Using Health Metrics to Monitor and Evaluate the Impact of Health Policies," conducted at the Tokyo Foundation for Policy Research. HM was also supported by the JSPS KAKENHI Grant (21K13454) and the Ministry of Education, Culture, Sports,

For peer review only - http://bmjopen.bmj.com/site/about/guidelines.xhtml

Science and Technology (MEXT) KAKENHI Grant (20H05805).

Competing interests: None declared.

Data availability statement: All de-identified data from participants reported in this study are available to interested researchers who submit data-sharing requests to the last author with a summary of the secondary analysis plan.

ORCID iDs:

Haruyo Mitani https://orcid.org/0000-0001-8882-5098

Naoki Kondo https://orcid.org/0000-0002-6425-6844

Airi Amemiya https://orcid.org/0000-0001-5319-9541

Takahiro Tabuchi http://orcid.org/0000-0002-1050-3125

REFERENCES

1 Felitti VJ, Anda RF, Nordenberg D, *et al.* Relationship of childhood abuse and household dysfunction to many of the leading causes of death in adults. The Adverse Childhood Experiences (ACE) Study. *Am J Prev Med* 1998;**14**:245–58. doi:10.1016/s0749-3797(98)00017-8

2 Anda RF, Felitti VJ, Bremner JD, *et al.* The enduring effects of abuse and related adverse experiences in childhood: a convergence of evidence from neurobiology and epidemiology. *Eur Arch Psychiatry Clin Neurosci* 2006;**256**:174–86. doi:10.1007/s00406-005-0624-4

3 Bellis MA, Hughes K, Ford K, *et al.* Life course health consequences and associated annual costs of adverse childhood experiences across Europe and North America: a systematic review and meta-analysis. *Lancet Public Heal* 2019;**4**:e517–28. doi:10.1016/S2468-2667(19)30145-8

4 Hughes K, Bellis MA, Hardcastle, A K, *et al.* The effect of multiple adverse childhood experiences on health: a systematic review and meta-analysis. *Lancet Public Heal* 2017;**2**:e356–66.https://doi.org/10.1016/S2468-2667(17)30118-4.

For peer review only - http://bmjopen.bmj.com/site/about/guidelines.xhtml

5 Norman RE, Byambaa M, De R, *et al.* The long-term health consequences of child physical abuse, emotional abuse, and neglect: a systematic review and meta-analysis. *Plos Med* 2012;**9**:1–31. doi:10.1371/journal.pmed.1001349

6 Petruccelli K, Davis J, Berman T. Adverse childhood experiences and associated health outcomes: a systematic review and meta-analysis. *Child Abus Negl* 2019;**97**:104127. doi:10.1016/j.chiabu.2019.104127

7 Oral R, Ramirez M, Coohey C, *et al.* Adverse childhood experiences and trauma informed care: the future of health care. *Pediatr Res* 2016;**79**:227–33. doi:10.1038/pr.2015.197

8 Shonkoff JP, Garner AS, Siegel BS, *et al.* The lifelong effects of early childhood adversity and toxic stress. *Pediatrics* 2012;**129**:e232–46. doi:10.1542/PEDS.2011-2663

9 Bellis MA, Hardcastle K, Ford K, *et al.* Does continuous trusted adult support in childhood impart life-course resilience against adverse childhood experiences - a retrospective study on adult health-harming behaviours and mental well-being. *BMC Psychiatry* 2017;**17**:110. doi:10.1186/S12888-017-1260-Z/TABLES/3

10 Crouch E, Radcliff E, Strompolis M, *et al.* Safe, stable, and nurtured: protective factors against poor physical and mental health outcomes following exposure to adverse childhood experiences (ACEs). *J Child Adolesc Trauma* 2019;**12**:165–73. doi:10.1007/S40653-018-0217-9/METRICS

11 Bethell C, Jones J, Gombojav N, *et al.* Positive childhood experiences and adult mental and relational health in a statewide sample: associations across adverse childhood experiences levels. *JAMA Pediatr* 2019;**173**:e193007. doi:10.1001/jamapediatrics.2019.3007

12 Masten AS, Cicchetti D. Resilience in development: progress and transformation. In: D. Cicchetti, ed. *Developmental Psychopathology, Risk, Resilience, and Intervention*. John Wiley & Sons, Inc. 2016. 271–333. doi:https://doi.org/10.1002/9781119125556.devpsy406

13 Gartland D, Riggs E, Muyeen S, *et al.* What factors are associated with resilient outcomes in children exposed to social adversity? a systematic review. *BMJ Open* 2019;**9**:e024870. doi:10.1136/BMJOPEN-2018-024870

14 Henson M, Sabo S, Trujillo A, *et al.* Identifying protective factors to promote health in american indian and alaska native adolescents: a literature review. *J Prim Prev*

For peer review only

For peer review only - http://bmjopen.bmj.com/site/about/guidelines.xhtml

2017;**38**:5–26. doi:10.1007/S10935-016-0455-2/METRICS

15 Okwori G. Role of individual, family, and community resilience in moderating effects of adverse childhood experiences on mental health among children. *J Dev Behav Pediatr* 2022;**43**:E452–62. doi:10.1097/DBP.0000000000001076

16 Molloy C, Beatson R, Harrop C, *et al.* Systematic review: effects of sustained nurse home visiting programs for disadvantaged mothers and children. *J Adv Nurs* 2021;**77**:147–61. doi:10.1111/JAN.14576

17 Isumi A, Doi S, Ochi M, *et al.* School- and community-level protective factors for resilience among chronically maltreated children in Japan. *Soc Psychiatry Psychiatr Epidemiol* 2023;**58**:477–88. doi:10.1007/S00127-022-02322-X/TABLES/5

18 Amemiya A, Fujiwara T, Shirai K, *et al.* Association between adverse childhood experiences and adult diseases in older adults: a comparative cross-sectional study in Japan and Finland. *BMJ Open* 2019;**9**:e024609. doi:10.1136/bmjopen-2018-024609

19 Fujiwara T, Kawakami N. Association of childhood adversities with the first onset of mental disorders in Japan: results from the World Mental Health Japan, 2002-2004. *J Psychiatr Res* 2011;**45**:481–7. doi:10.1016/j.jpsychires.2010.08.002

20 Cohen S, Gianaros PJ, Manuck SB. A stage model of stress and disease. *Perspect Psychol Sci* 2016;**11**:456–63. doi:10.1177/1745691616646305

21 Mueller KC, Carey MT. How positive and negative childhood experiences interact with resiliency theory and the general theory of crime in juvenile probationers. *Youth Violence Juv Justice* 2022;**21**:130–48. doi:10.1177/15412040221131278

22 Zimmerman MA. Resiliency theory: a strengths-based approach to research and practice for adolescent health. *Heal Educ Behav* 2013;**40**:381–3. doi:10.1177/1090198113493782

23 Oshri A, Duprey EK, Liu S, *et al.* ACEs and resilience: methodological and conceptual issues. In: Asmundson GJG, Afifi TO, eds. *Adverse Childhood Experiences: Using Evidence to Advance Research, Practice, Policy, and Prevention*. Academic Press 2020. 287–306.

24 Narayan AJ, Rivera LM, Bernstein RE, *et al.* Positive childhood experiences predict less psychopathology and stress in pregnant women with childhood adversity: a pilot study of the benevolent childhood experiences (BCEs) scale. *Child Abuse Negl* 2018;**78**:19–

For peer review only

For peer review only - http://bmjopen.bmj.com/site/about/guidelines.xhtml

30. doi:10.1016/J.CHIABU.2017.09.022

25 Crandall AA, Miller JR, Cheung A, *et al.* ACEs and counter-ACEs: how positive and negative childhood experiences influence adult health. *Child Abuse Negl* 2019;**96**:104089. doi:10.1016/J.CHIABU.2019.104089

26 Bellis MA, Hughes K, Ford K, *et al.* Adverse childhood experiences and sources of childhood resilience: a retrospective study of their combined relationships with child health and educational attendance. *BMC Public Health* 2018;**18**:1–12. doi:10.1186/S12889-018-5699-8/FIGURES/1

27 JACSIS study. The Japan COVID-19 and Society Internet Survey. 2021.https://jacsis-study.jp/

28 Fujiwara T. Impact of adverse childhood experience on physical and mental health: a life-course epidemiology perspective. *Psychiatry Clin Neurosci* 2022;**76**:544–51. doi:10.1111/PCN.13464

29 Cheong E Von, Sinnott C, Dahly D, *et al.* Adverse childhood experiences (ACEs) and later-life depression: perceived social support as a potential protective factor. *BMJ Open* 2017;**7**:e013228. doi:10.1136/BMJOPEN-2016-013228

30 Furukawa TA, Kawakami N, Saitoh M, *et al.* The performance of the Japanese version of the K6 and K10 in the World Mental Health Survey Japan. *Int J Methods Psychiatr Res* 2008;**17**:152–8. doi:10.1002/mpr.257

31 Kessler RC, Andrews G, Colpe LJ, *et al.* Short screening scales to monitor population prevalences and trends in non-specific psychological distress. *Psychol Med* 2002;**32**:959–76. doi:10.1017/S0033291702006074

32 Kessler RC, Green JG, Gruber MJ, *et al.* Screening for serious mental illness in the general population with the K6 screening scale: results from the WHO World Mental Health (WMH) survey initiative. *Int J Methods Psychiatr Res* 2010;**19**:4–22. doi:10.1002/mpr.310

33 Metzler M, Merrick MT, Klevens J, *et al.* Adverse childhood experiences and life opportunities: shifting the narrative. *Child Youth Serv Rev* 2017;**72**:141–9. doi:10.1016/j.childyouth.2016.10.021

34 Kuhar M, Zager Kocjan G. Associations of adverse and positive childhood experiences with adult physical and mental health and risk behaviours in Slovenia. *Eur J*

For peer review only

For peer review only - http://bmjopen.bmj.com/site/about/guidelines.xhtml

Psychotraumatol 2021;**12**:1924953. doi:10.1080/20008198.2021.1924953

35 Mize TD. Best practices for estimating, interpreting, and presenting nonlinear interaction effects. *Sociol Sci* 2019;**6**:81–117. doi:10.15195/V6.A4

36 Hair JF, Black B, Babin BJ, *et al. Multivariate Data Analysis: Global Edition*. 7th ed. Pearson Education 2009.

37 Aiken LS, West SG. *Multiple Regression: Testing and Interpreting Interactions*. Newbury Park, CA: Sage Publications 1991.

38 Rhodes JE, Spencer R, Keller TE, *et al.* A model for the influence of mentoring relationships on youth development. *J Community Psychol* 2006;**34**:691–707. doi:10.1002/JCOP.20124

39 Graber R, Turner R, Madill A. Best friends and better coping: facilitating psychological resilience through boys' and girls' closest friendships. *Br J Psychol* 2016;**107**:338–58. doi:10.1111/BJOP.12135

40 Sege RD, Harper Browne C. Responding to ACEs with HOPE: Health Outcomes from Positive Experiences. *Acad Pediatr* 2017;**17**:S79–85. doi:10.1016/J.ACAP.2017.03.007

41 The National Resource Center at Tufts Medical Center. Tufts HOPE: Healthy Outcomes from Positive Experiences. 2023.https://positiveexperience.org/

42 Nishio A, Kakimoto M, Horita R, *et al.* Compulsory educational mental health support system in Japan. *Pediatr Int* 2020;**62**:529–34. doi:10.1111/PED.14205

43 Kanazawa M. Current issues of school social work in Japan : from the historical relations between school, social work, and counseling. *Japanese J Soc Serv* 2009;**5**:159–71. doi:10.24469/JJSWE.5.0_159

44 Murayama H, Kurotani K, Tabuchi T. Social capital and the spread of children's cafeterias (kodomo shokudo) in Japan: an ecological analysis. *Asia-Pacific J public Heal* 2022;**34**:817–20. doi:10.1177/10105395221119416

Figure legends

Figure 1

For peer review only - http://bmjopen.bmj.com/site/about/guidelines.xhtml

A heatmap of the adjusted odds ratios with 95% CIs for the health outcomes of ACEs (≥1 vs. 0) across CPCE exposure levels. All odds ratios are adjusted for age, sex, educational attainment, and FPCEs. The significance level of data in bold font is <0.05. *P value for the interaction effect of ACEs (≥1 vs. 0) × CPCEs (cumulative variable). †P value for interaction effect of ACEs (≥1 vs. 0) × CPCEs (≥3+ vs. 0–2).

Figure 2

Adjusted predictions with 95% CIs for the health outcomes of ACEs (≥1 vs. 0) × CPCEs (≥3 vs. 0–2). Substitutions were performed on the multiple logistic regression models presented in Supplementary Table S6. *P value for significant differences of marginal effects between 0–2 and ≥3 CPCEs.

For peer review only - http://bmjopen.bmj.com/site/about/guidelines.xhtml

	0 CPCEs N=11051	1 CPCE N=5543	2 CPCEs N=4502	3 CPCEs N=3922	4 CPCEs N=3599	P value*	P value†
**Diabetes**	**1.35** (1.15–1.58)	**1.34** (1.09–1.65)	**1.35** (1.07–1.71)	1.27 (0.96–1.69)	0.93 (0.64–1.34)	0.487	0.341
**Ischemic heart disease**	**1.82** (1.40–2.37)	**1.62** (1.19–2.20)	**1.57** (1.11–2.23)	0.65 (0.38–1.13)	1.14 (0.61–2.15)	0.044	0.007
**Stroke**	**1.77** (1.29–2.43)	**2.01** (1.38–2.94)	**2.58** (1.67–4.01)	1.47 (0.75–2.88)	1.08 (0.42–2.81)	0.844	0.213
**COPD**	**1.88** (1.30–2.70)	**2.27** (1.51–3.41)	**2.75** (1.80–4.22)	0.82 (0.40–1.67)	0.47 (0.10–2.16)	0.406	0.003
**Cancer**	**1.61** (1.23–2.12)	**2.39** (1.75–3.27)	**1.50** (1.07–2.10)	0.99 (0.62–1.60)	1.01 (0.58–1.76)	0.052	0.012
**Chronic pain**	**2.03** (1.84–2.24)	**1.55** (1.35–1.79)	**1.51** (1.29–1.77)	**1.47** (1.22–1.78)	**1.40** (1.12–1.74)	0.000	0.012
**Depression**	**3.49** (2.96–4.12)	**2.60** (2.00–3.38)	**1.94** (1.44–2.62)	**2.13** (1.40–3.24)	**2.02** (1.22–3.37)	0.000	0.049
**Suicidal ideation**	**2.85** (2.62–3.09)	**2.06** (1.83–2.32)	**2.06** (1.80–2.36)	**1.82** (1.54–2.14)	**1.96** (1.60–2.40)	0.002	0.024
**Severe psychological distress**	**2.68** (2.39–3.00)	**1.89** (1.57–2.27)	**1.82** (1.45–2.30)	**1.74** (1.27–2.39)	**2.09** (1.40–3.12)	0.000	0.091

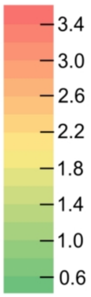

Figure 1

A heatmap of the adjusted odds ratios with 95% CIs for the health outcomes of ACEs (≥1 vs. 0) across CPCE exposure levels. All odds ratios are adjusted for age, sex, educational attainment, and FPCEs. The significance level of data in bold font is <0.05. *P value for the interaction effect of ACEs (≥1 vs. 0) × CPCEs (cumulative variable). †P value for interaction effect of ACEs (≥1 vs. 0) × CPCEs (≥3+ vs. 0–2).

130x97mm (1200 x 1200 DPI)

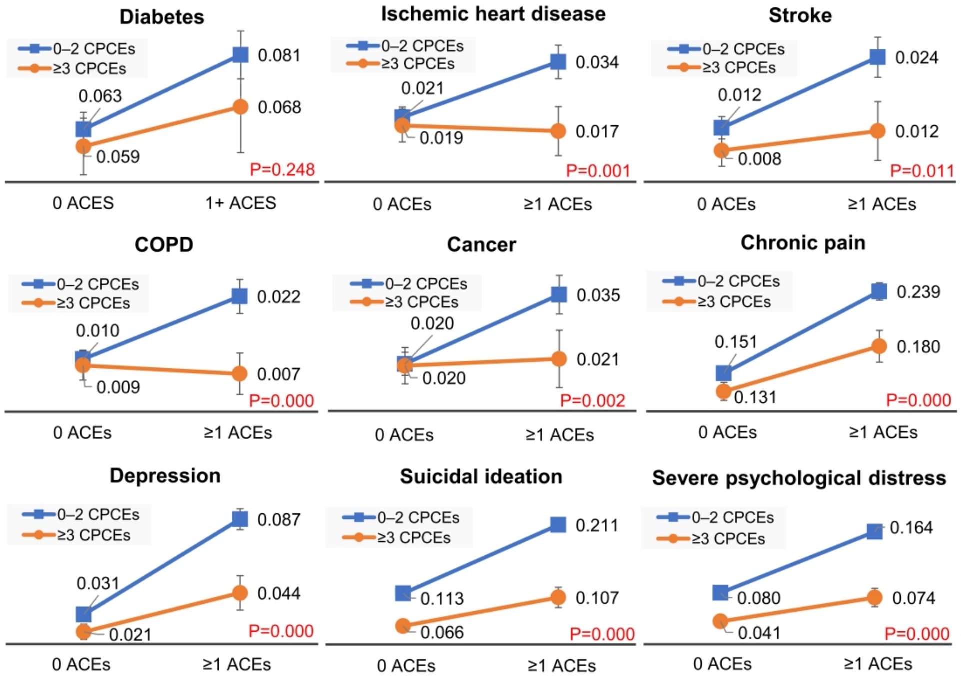

Figure 2

Adjusted predictions with 95% CIs for the health outcomes of ACEs (≥1 vs. 0) × CPCEs (≥3 vs. 0–2). Substitutions were performed on the multiple logistic regression models presented in Supplementary Table S6. *P value for significant differences of marginal effects between 0–2 and ≥3 CPCEs.

130x92mm (1200 x 1200 DPI)

Supplemental Material

Table S1 Prevalence of PCE single items among participants (N=28617)

	N	weighted %
FPCEs		
i) Felt able to talk to their family about feelings	9113	28.6
ii) Felt their family stood by them during difficult times	12738	40.0
iii) Felt safe and protected by an adult in their home	14700	45.8
CPCEs		
iv) Had at least 2 nonparent adults who took genuine interest in them	10732	32.8
v) Felt supported by friends	10563	32.1
vi) Felt a sense of belonging in middle and high school	12334	37.9
vii) Enjoyed participating in community traditions	7080	22.1

FPCEs, family positive childhood experiences; CPCEs, community positive childhood experiences

Table S2 Summary of results of psychometric analyses performed on FPCEs and CPCEs

	Internal Consistency (Cronbach's alpha)	Principle components factor analysis results	Factor loading across items
FPCEs	0.81	A single Eigen value greater than 1.0 (2.19) that explained 72.9% of variance	0.54 ("felt able to talk") to 0.61 ("felt safe and protected")
CPCEs	0.75	A single Eigen value greater than 1.0 (2.28) that explained 56.9% of variance	0.46 ("community traditions") to 0.52 ("felt supported by friends")

FPCEs, family positive childhood experiences; CPCEs, community positive childhood experiences

For peer review only

Table S3 Demographic characteristics and prevalence of health outcomes, PCEs by ACE exposure levels

	Prevalence of ACEs				
	0 ACEs	1 ACE	2–3 ACEs	≥4 ACEs	
	N=17889	N=5547	N=3725	N=1456	
	W %/M	W %/M	W %/M	W %/M	P value*
Health outcomes					
Diabetes	6.9	9.1	7.6	10.5	<0.001
Ischemic heart disease	2.7	2.8	3.0	6.8	<0.001
Stroke	1.2	1.4	2.3	5.6	<0.001
COPD	1.1	0.8	2.2	5.7	<0.001
Cancer	2.0	2.8	3.3	6.7	<0.001
Chronic pain	14.7	20.9	26.7	33.1	<0.001
Depression	2.8	3.9	8.6	17.7	<0.001
Suicidal ideation	10.3	13.3	25.7	35.7	<0.001
Severe psychological distress	7.0	8.9	19.0	30.6	<0.001
FPCEs (0–3)	1.3	1.1	0.7	0.3	<0.001
0 FPCE	39.9	47.7	65.0	80.2	<0.001
1 FPCEs	14.4	13.6	13.9	10.7	0.095
2 FPCEs	17.9	17.1	11.8	5.4	<0.001
3 FPCEs	27.8	21.7	9.4	3.7	<0.001
CPCEs (0–4)	1.4	1.2	0.9	0.7	<0.001
0 CPCE	40.4	43.9	52.8	61.8	<0.001
1 CPCEs	18.3	21.1	21.8	18.7	<0.001
2 CPCEs	14.9	15.7	13.0	12.6	0.002
3 CPCEs	13.3	10.4	8.0	4.4	<0.001
4 CPCEs	13.1	8.9	4.5	2.6	<0.001
Age (19–79)	47.4	51.3	47.2	45.2	<0.001
Sex					
Male	52.8	46.2	39.8	38.5	<0.001
Female	47.2	53.8	60.2	61.5	<0.001
Educational attainment					
Less than HS	2.9	7.5	5.0	9.0	<0.001
HS graduate	57.8	62.9	64.8	66.8	<0.001
Post-secondary and graduate	37.8	28.6	28.7	23.1	<0.001

*P value for significant differences by Pearson's χ^2 test or ANOVA.

W, weighted; ACEs, adverse childhood experiences; FPCEs, family positive childhood experiences; CPCEs, community positive childhood experiences; HS, high school

For peer review only

Table S4 Adjusted odds ratios of health outcomes by ACE exposure levels

	Diabetes		Ischemic heart disease		Stroke		COPD		Cancer		Chronic pain		Depression		Suicidal ideation		Severe psychological distress	
	AOR	(95% CI)	AOR	(95% CI)	AOR	(95% CI)	AOR	(95% CI)	AOR	(95% CI)	AOR	(95% CI)	AOR	(95% CI)	AOR	(95% CI)	AOR	(95% CI)
ACEs																		
0 ACEs [reference]	1.00		1.00		1.00		1.00		1.00		1.00		1.00		1.00		1.00	
1 ACE	1.16	(1.02–1.31)	1.08	(0.88–1.33)	1.24	(0.95–1.63)	0.98	(0.71–1.35)	1.06	(0.86–1.30)	1.41	(1.30–1.53)	1.75	(1.49–2.04)	1.65	(1.51–1.82)	1.65	(1.48–1.84)
2–3 ACEs	1.43	(1.24–1.65)	1.78	(1.44–2.21)	2.28	(1.76–2.95)	2.62	(2.01–3.41)	1.90	(1.55–2.34)	1.94	(1.78–2.11)	3.81	(3.30–4.40)	3.16	(2.88–3.47)	3.25	(2.93–3.61)
4 ACEs	1.98	(1.62–2.42)	3.52	(2.73–4.54)	5.21	(3.94–6.89)	5.66	(4.24–7.56)	3.71	(2.90–4.73)	2.93	(2.60–3.30)	8.12	(6.90–9.56)	4.29	(3.78–4.86)	5.94	(5.20–6.78)
Age	1.04	(1.04–1.05)	1.02	(1.01–1.02)	0.99	(0.99–1.00)	0.98	(0.97–0.99)	1.02	(1.02–1.03)	1.01	(1.01–1.01)	0.98	(0.98–0.98)	0.96	(0.96–0.97)	0.96	(0.96–0.96)
Sex																		
Male [reference]	1.00		1.00		1.00		1.00		1.00		1.00		1.00		1.00		1.00	
Female	0.31	(0.28–0.35)	0.34	(0.29–0.40)	0.38	(0.31–0.47)	0.32	(0.26–0.40)	0.73	(0.63–0.85)	1.13	(1.06–1.20)	0.62	(0.55–0.69)	1.02	(0.95–1.09)	0.88	(0.81–0.95)
Educational attainment																		
Less than HS	1.64	(1.22–2.20)	1.29	(0.80–2.07)	0.99	(0.52–1.90)	1.24	(0.74–2.45)	0.75	(0.43–1.32)	1.22	(0.98–1.51)	1.60	(1.17–2.19)	1.66	(1.32–2.09)	1.55	(1.20–1.99)
HS graduate	1.13	(1.02–1.25)	1.05	(0.90–1.24)	1.04	(0.85–1.28)	0.99	(0.79–1.23)	0.82	(0.70–0.97)	1.11	(1.04–1.18)	1.11	(0.99–1.25)	1.08	(1.00–1.16)	1.02	(0.93–1.11)
Post-secondary and graduate [reference]	1.00		1.00		1.00		1.00		1.00		1.00		1.00		1.00		1.00	

AOR, adjusted odds ratio; ACEs, adverse childhood experiences; HS, high school

For peer review only

Table S5 Adjusted odds ratios of health outcomes by ACEs, FPCEs, and CPCEs

	Diabetes		Ischemic heart disease		Stroke		COPD		Cancer		Chronic pain		Depression		Suicidal ideation		Severe psychological distress	
	**AOR**	**(95% CI)**	**AOR**	**(95% CI)**	**AOR**	**(95% CI)**	**AOR**	**(95% CI)**	**AOR**	**(95% CI)**	**AOR**	**(95% CI)**	**AOR**	**(95% CI)**	**AOR**	**(95% CI)**	**AOR**	**(95% CI)**
ACEs																		
0 ACEs [reference]	1.00		1.00		1.00		1.00		1.00		1.00		1.00		1.00		1.00	
≥1 ACEs	1.30	(1.18–1.44)	1.52	(1.30–1.78)	1.92	(1.58–2.33)	1.95	(1.58–2.41)	1.60	(1.37–1.87)	1.70	(1.60–1.82)	2.85	(2.54–3.20)	2.12	(1.97–2.28)	2.28	(2.10–2.48)
FPCEs																		
0–1 FPCEs [reference]	1.00		1.00		1.00		1.00		1.00		1.00		1.00		1.00		1.00	
2 FPCEs	0.99	(0.86–1.15)	0.99	(0.80–1.24)	0.77	(0.59–1.01)	0.77	(0.58–1.02)	0.86	(0.69–1.06)	1.00	(0.91–1.10)	0.88	(0.74–1.06)	0.83	(0.74–0.93)	0.81	(0.71–0.93)
3 FPCEs	0.81	(0.73–0.98)	0.70	(0.55–0.90)	0.50	(0.37–0.70)	0.40	(0.28–0.58)	0.68	(0.54–0.85)	0.95	(0.87–1.05)	0.69	(0.56–0.84)	0.62	(0.55–0.70)	0.64	(0.56–0.74)
CPCEs																		
0–2 CPCEs [reference]	1.00		1.00		1.00		1.00		1.00		1.00		1.00		1.00		1.00	
3 CPCEs	0.96	(0.83–1.12)	0.80	(0.62–1.03)	0.63	(0.44–0.89)	0.76	(0.54–1.07)	0.82	(0.84–1.05)	0.82	(0.74–0.91)	0.62	(0.50–0.78)	0.56	(0.49–0.64)	0.52	(0.45–0.62)
4 CPCEs	0.79	(0.66–0.95)	0.65	(0.47–0.89)	0.52	(0.34–0.82)	0.38	(0.22–0.66)	0.80	(0.61–1.06)	0.75	(0.67–0.84)	0.55	(0.42–0.72)	0.45	(0.38–0.53)	0.40	(0.32–0.49)
Age	1.04	(1.04–1.04)	1.02	(1.01–1.02)	0.99	(0.98–1.00)	0.98	(0.97–0.98)	1.02	(1.01–1.02)	1.01	(1.01–1.01)	0.98	(0.98–0.98)	0.96	(0.96–0.96)	0.96	(0.95–0.96)
Sex																		
Male [reference]	1.00		1.00		1.00		1.00		1.00		1.00		1.00		1.00		1.00	
Female	0.32	(0.29–0.35)	0.36	(0.30–0.42)	0.41	(0.34–0.51)	0.35	(0.28–0.44)	0.75	(0.64–0.87)	1.17	(1.10–1.24)	0.70	(0.63–0.78)	1.17	(1.09–1.26)	1.02	(0.94–1.11)
Educational attainment																		
Less than HS	1.70	(1.27–2.28)	1.45	(0.90–2.33)	1.17	(0.62–2.23)	1.69	(0.93–3.06)	0.90	(0.51–1.57)	1.27	(1.03–1.58)	1.67	(1.23–2.28)	1.57	(1.25–1.97)	1.49	(1.16–1.91)
HS graduate	1.14	(1.03–1.26)	1.08	(0.92–1.27)	1.07	(0.87–1.31)	1.03	(0.82–1.28)	0.85	(0.73–1.00)	1.11	(1.05–1.19)	1.10	(0.98–1.23)	1.03	(0.95–1.11)	0.97	(0.89–1.06)
Post-secondary and graduate [reference]	1.00		1.00		1.00		1.00		1.00		1.00		1.00		1.00		1.00	

AOR, adjusted odds ratio; ACEs, adverse childhood experiences; FPCEs, family positive childhood experiences; CPCEs, community positive childhood; HS, high school

For peer review only

Table S6 Adjusted odds ratios of health outcomes by ACEs, CPCEs, and these interaction terms

	Diabetes		Ischemic heart disease		Stroke		COPD		Cancer		Chronic pain		Depression		Suicidal ideation		Severe psychological distress	
	AOR	(95% CI)	AOR	(95% CI)	AOR	(95% CI)	AOR	(95% CI)	AOR	(95% CI)	AOR	(95% CI)	AOR	(95% CI)	AOR	(95% CI)	AOR	(95% CI)
ACEs																		
0 ACEs [reference]	1.00		1.00		1.00		1.00		1.00		1.00		1.00		1.00		1.00	
≥1 ACEs	1.34	(1.20–1.49)	1.68	(1.42–2.00)	2.02	(1.63–2.49)	2.24	(1.78–2.81)	1.77	(1.49–2.11)	1.78	(1.65–1.91)	2.99	(2.63–3.38)	2.20	(2.03–2.37)	2.35	(2.15–2.56)
CPCEs																		
0–2 CPCEs [reference]	1.00		1.00		1.00		1.00		1.00		1.00		1.00		1.00		1.00	
≥3 CPCEs	0.93	(0.79–1.08)	0.55	(0.70–1.16)	0.68	(0.47–0.98)	0.88	(0.61–1.27)	0.98	(0.77–1.25)	0.85	(0.77–0.94)	0.67	(0.52–0.85)	0.55	(0.47–0.63)	0.48	(0.41–0.57)
Interaction																		
≥1 ACEs × ≥3 CPCEs	0.89	(0.70–1.13)	0.50	(0.36–0.85)	0.70	(0.39–1.23)	0.37	(0.19–0.74)	0.61	(0.41–0.90)	0.82	(0.70–0.96)	0.71	(0.51–1.00)	0.79	(0.64–0.97)	0.80	(0.62–1.04)
Age	1.04	(1.04–1.05)	1.02	(1.01–1.02)	0.99	(0.98–1.00)	0.98	(0.97–0.99)	1.02	(1.01–1.02)	1.01	(1.01–1.01)	0.98	(0.98–0.98)	0.96	(0.96–0.96)	0.96	(0.95–0.96)
Sex																		
Male [reference]	1.00		1.00		1.00		1.00		1.00		1.00		1.00		1.00		1.00	
Female	0.32	(0.29–0.35)	0.35	(0.30–0.42)	0.41	(0.33–0.51)	0.35	(0.27–0.43)	0.74	(0.64–0.87)	1.17	(1.09–1.24)	0.69	(0.62–0.77)	1.15	(1.07–1.24)	1.01	(0.93–1.09)
Educational attainment																		
Less than HS	1.70	(1.27–2.27)	1.43	(0.89–2.30)	1.16	(0.61–2.22)	1.66	(0.91–3.01)	0.88	(0.50–1.55)	1.27	(1.02–1.57)	1.71	(1.26–2.33)	1.60	(1.28–2.01)	1.54	(1.20–1.98)
HS graduate	1.14	(1.03–1.26)	1.08	(0.92–1.27)	1.07	(0.87–1.31)	1.02	(0.82–1.27)	0.86	(0.73–1.00)	1.12	(1.05–1.19)	1.11	(0.99–1.24)	1.03	(0.96–1.12)	0.98	(0.90–1.07)
Post-secondary and graduate [reference]	1.00		1.00		1.00		1.00		1.00		1.00		1.00		1.00		1.00	
FPCEs																		
0–1 FPCEs [reference]	1.00		1.00		1.00		1.00		1.00		1.00		1.00		1.00		1.00	
2 FPCEs	0.99	(0.86–1.15)	0.99	(0.80–1.24)	0.77	(0.58–1.01)	0.76	(0.57–1.01)	0.86	(0.69–1.06)	1.00	(0.91–1.10)	0.79	(0.67–0.93)	0.75	(0.67–0.83)	0.68	(0.60–0.77)
3 FPCEs	0.83	(0.72–0.96)	0.68	(0.54–0.87)	0.49	(0.36–0.68)	0.36	(0.25–0.52)	0.67	(0.53–0.83)	0.94	(0.86–1.03)	0.61	(0.50–0.73)	0.55	(0.49–0.61)	0.53	(0.46–0.60)

AOR, adjusted odds ratio ACEs, adverse childhood experiences; CPCEs, community positive childhood; HS, high school; FPCEs, family positive childhood experiences

For peer review only

Table S7 Marginal effects with test of interaction effects

	**Marginal effects (95% CI)**		**Differences of two marginal effects (95% CI)**	P value*
	0–2 CPCEs	≥3 CPCEs		
Diabetes	0.018 (0.011 to 0.025)	0.010 (−0.003 to 0.022)	0.008 (−0.023 to 0.006)	0.248
Ischemic heart disease	0.013 (0.009 to 0.018)	−0.001 (−0.008 to 0.006)	0.015 (0.006 to 0.023)	0.001
Stroke	0.012 (0.008 to 0.016)	0.003 (−0.002 to 0.009)	0.009 (0.006 to 0.023)	0.011
COPD	0.012 (0.008 to 0.016)	−0.002 (−0.006 to 0.003)	0.014 (0.008 to 0.020)	0.000
Cancer	0.015 (0.010 to 0.020)	0.001 (−0.006 to 0.009)	0.013 (0.005 to 0.022)	0.002
Chronic pain	0.088 (0.077 to 0.099)	0.049 (0.030 to 0.068)	0.039 (0.017 to 0.061)	0.000
Depression	0.055 (0.049 to 0.062)	0.023 (0.012 to 0.033)	0.033 (0.020 to 0.046)	0.000
Suicidal ideation	0.098 (0.088 to 0.108)	0.041 (0.025 to 0.057)	0.057 (0.038 to 0.076)	0.000
Severe psychological distress	0.084 (0.075 to 0.092)	0.033 (0.019 to 0.046)	0.051 (0.035 to 0.067)	0.000

*P value for significant differences by test of the equality of marginal effects (Stata: margins, contrast).

For peer review only

STROBE Statement—Checklist of items that should be included in reports of ***cross-sectional studies***

	Item No	Recommendation	Page No
**Title and abstract**	1	(*a*) Indicate the study's design with a commonly used term in the title or the abstract	1-2
		(*b*) Provide in the abstract an informative and balanced summary of what was done and what was found	2
**Introduction**			
Background/rationale	2	Explain the scientific background and rationale for the investigation being reported	4
Objectives	3	State specific objectives, including any prespecified hypotheses	5
**Methods**			
Study design	4	Present key elements of study design early in the paper	4-5
Setting	5	Describe the setting, locations, and relevant dates, including periods of recruitment, exposure, follow-up, and data collection	6
Participants	6	(*a*) Give the eligibility criteria, and the sources and methods of selection of participants	6
Variables	7	Clearly define all outcomes, exposures, predictors, potential confounders, and effect modifiers. Give diagnostic criteria, if applicable	7-9
Data sources/ measurement	8*	For each variable of interest, give sources of data and details of methods of assessment (measurement). Describe comparability of assessment methods if there is more than one group	*7-9*
Bias	9	Describe any efforts to address potential sources of bias	6
Study size	10	Explain how the study size was arrived at	6
Quantitative variables	11	Explain how quantitative variables were handled in the analyses. If applicable, describe which groupings were chosen and why	7-9
Statistical methods	12	(*a*) Describe all statistical methods, including those used to control for confounding	9
		(*b*) Describe any methods used to examine subgroups and interactions	9
		(*c*) Explain how missing data were addressed	6
		(*d*) If applicable, describe analytical methods taking account of sampling strategy	9
		(*e*) Describe any sensitivity analyses	9
**Results**			
Participants	13*	(a) Report numbers of individuals at each stage of study—eg numbers potentially eligible, examined for eligibility, confirmed eligible, included in the study, completing follow-up, and analysed	6
		(b) Give reasons for non-participation at each stage	6
		(c) Consider use of a flow diagram	6
Descriptive data	14*	(a) Give characteristics of study participants (eg demographic, clinical, social) and information on exposures and potential confounders	9-11
		(b) Indicate number of participants with missing data for each variable of interest	9-11
Outcome data	15*	Report numbers of outcome events or summary measures	10
Main results	16	(*a*) Give unadjusted estimates and, if applicable, confounder-adjusted estimates and their precision (eg, 95% confidence interval). Make clear which confounders were adjusted for and why they were included	11-13

For peer review only - http://bmjopen.bmj.com/site/about/guidelines.xhtml

		(*b*) Report category boundaries when continuous variables were categorized	10-12
		(*c*) If relevant, consider translating estimates of relative risk into absolute risk for a meaningful time period	11-13
Other analyses	17	Report other analyses done—eg analyses of subgroups and interactions, and sensitivity analyses	12-13
**Discussion**			
Key results	18	Summarise key results with reference to study objectives	13
Limitations	19	Discuss limitations of the study, taking into account sources of potential bias or imprecision. Discuss both direction and magnitude of any potential bias	15
Interpretation	20	Give a cautious overall interpretation of results considering objectives, limitations, multiplicity of analyses, results from similar studies, and other relevant evidence	13-15
Generalisability	21	Discuss the generalisability (external validity) of the study results	13-15
**Other information**			
Funding	22	Give the source of funding and the role of the funders for the present study and, if applicable, for the original study on which the present article is based	17

*Give information separately for exposed and unexposed groups.

Note: An Explanation and Elaboration article discusses each checklist item and gives methodological background and published examples of transparent reporting. The STROBE checklist is best used in conjunction with this article (freely available on the Web sites of PLoS Medicine at http://www.plosmedicine.org/, Annals of Internal Medicine at http://www.annals.org/, and Epidemiology at http://www.epidem.com/). Information on the STROBE Initiative is available at www.strobe-statement.org.

For peer review only

For peer review only - http://bmjopen.bmj.com/site/about/guidelines.xhtml
